# Cooperative coding of continuous variables in networks with sparsity constraint

**DOI:** 10.1371/journal.pcbi.1012156

**Published:** 2025-07-03

**Authors:** Paul Züge, Natalie Schieferstein, Raoul-Martin Memmesheimer

**Affiliations:** Institute for Genetics, University of Bonn, Bonn, Germany; Universitatsklinikum Hamburg-Eppendorf, GERMANY

## Abstract

A hallmark of biological and artificial neural networks is that neurons tile the range of continuous sensory inputs and intrinsic variables with overlapping responses. It is characteristic for the underlying recurrent connectivity in the cortex that neurons with similar tuning predominantly excite each other. The reason for such an architecture is not clear. Using an analytically tractable model as well as spiking neural networks, we show that it can naturally arise from a cooperative coding scheme. In this scheme neurons with similar responses specifically support each other by sharing their computations to obtain the desired population code. This sharing allows each neuron to effectively respond to a broad variety of inputs, while only receiving few feedforward and recurrent connections. Few strong, specific recurrent connections then replace many feedforward and less specific recurrent connections, such that the resulting connectivity optimizes the number of required synapses. This suggests that the number of required synapses may be a crucial constraining factor in biological neural networks. Synaptic savings increase with the dimensionality of the encoded variables. We find a trade-off between saving synapses and response speed. The response speed improves by orders of magnitude when utilizing the window of opportunity between excitatory and delayed inhibitory currents that arises if, as found in experiments, spike frequency adaptation is present or strong recurrent excitation is balanced by strong, shortly-lagged inhibition.

## Author summary

Neurons represent continuous sensory or intrinsic variables in their joint activity, with rather broad and overlapping individual response profiles. In particular there are often many neurons with highly similar tuning. In the cortex, these neurons predominantly excite each other. We provide a new explanation for this type of recurrent excitation, showing that it can arise in a novel cooperative coding scheme that minimizes the number of required synapses. This suggests the number of required synapses as a crucial constraining factor in biological neural networks. In our cooperative coding scheme, neurons use few strong and specific excitatory connections to share their computations with those neurons that also need it. This way, neurons can generate a large part of their response by leveraging inputs from neurons with similar responses, requiring fewer feedforward inputs. We find a trade-off between saving synapses and response speed. Theoretical estimates and numerical simulations show that specific features of biological single neurons and neural networks can drastically increase the response speed, improving the trade-off.

## Introduction

The brain encodes continuous sensory or intrinsic variables in the coordinated activity of populations of neurons. The tuning curves (response profiles) of individual neurons in such populations are rather broad, leading to large overlaps between them [1,2]. Further, there are often many neurons with highly similar tuning. Neuron populations with such features include simple cells in the primary visual cortex (V1) [3,4], head direction cells in the anterior thalamic nucleus [5], tactile neurons in primary somatosensory cortex [6], place cells in the hippocampus [7] and grid cells in the medial entorhinal cortex [8,9]. In machine learning, convolutional networks have overlapping receptive fields (RFs) that tile the input space [10]. RFs similar to those in visual cortex emerge by learning a sparse code for natural images [11], and RFs similar to grid cells emerge through training on navigation tasks [12,13].

Neurobiological data show that neurons with strongly overlapping RFs are predominantly excitatorily coupled: Synaptic connections between similarly tuned excitatory principal neurons are more likely [14], stronger and more often bidirectional [15,16]. In line with this, the strongest incoming synapses provide excitation that matches a neuron’s RF [16,17]. Furthermore, highly similarly tuned principal neurons have overall, i.e., including indirect, polysynaptic connections, a net excitatory effect on each other [18,19]. In contrast, if the tuning is barely similar or dissimilar, the net effect is inhibitory.

Such recurrent excitatory connectivity may seem unintuitive from a normative standpoint, as it amplifies noise [20] and can increase response times [21,22]. Previous studies suggested that it may support persistent activity and thus working memory [23,24] or sampling-based inference [25].

Neural networks, however, evolved subject to physiological and physical constraints [26–29], including metabolic cost and available space. Optimizing for specific features can largely determine the neural network and lead to solutions that are in other aspects sub-optimal. A prominent example for this is a recent version of the efficient coding hypothesis [30–34]. It posits that neural networks greedily minimize the number of used spikes or the rate activity, which contribute to metabolic cost. The network connectivity obtained from the optimization is, however, very dense, which is not found in experiments. Further, the coding scheme is “competitive”, in the sense that similarly tuned neurons compete for the opportunity to generate spikes. In other words, such neurons take away spikes and activity from each other. This predicts inhibitory couplings between very similarly tuned neurons, contrary to the experimentally observed physiological and effective excitatory interconnectivity between them.

Here we explore the implications of “cooperative coding” in a neural network. In this newly proposed scheme, neurons avoid replicating computations through feedforward weights whose results are already accessible from the activity of other feature neurons. Instead, each feature neuron performs only a non-redundant feedforward computation. It then achieves the required response by additionally incorporating the results obtained by similarly tuned feature neurons through recurrent connections. In other words, feature neurons do not independently replicate shared parts of the computations through feedforward weights, but they transmit them through recurrent connections to each other. The resulting connectivity is like-to-like, i.e., strong and effectively excitatory between similarly tuned principal neurons, as observed in experiments. Interestingly the scheme can optimize the number of synapses in a network, while maintaining the required neural network dynamics. Such an optimization differs from the common focus on saving dynamical quantities such as spikes and may be imposed by space restrictions and the cost of maintaining synapses [28,35].

## Results

To demonstrate the concept of cooperative coding, we consider a layer of feature neurons (output neurons), which receive feedforward input from an input layer as well as recurrent input. The task of the feature neurons is to generate a weighted sum of the inputs with weight strengths that decay exponentially with the distance of an input from the preferred input. We assume that the functionally relevant network response, representing the desired features (outputs), is the steady state activity. The desired outputs are linear functions of the inputs. Neural responses can hence be characterized by linear RFs and implemented by feedforward connectivity alone. Importantly, they can also be implemented using mixtures of feedforward and recurrent input.

We will compare the different network implementations in terms of the space requirement, approximated by the number of required synapses, and in terms of the metabolic cost to keep up the stationary state. Furthermore, we will compare the response times and demonstrate how they can be substantially decreased in networks with spike frequency adaptation (SFA) or balancing inhibition. Finally we will verify that our findings translate to cooperative coding in spiking neural networks.

We will analyze three concrete examples of cooperative coding: (i) encoding a one-dimensional stimulus, (ii) simultaneously encoding two one-dimensional stimuli with linear mixed selectivity (MS) and (iii) encoding a two-dimensional stimulus. For ease of description, we focus on translationally invariant RFs. (Approximate) translational invariance, meaning that offset RFs have similar shapes, is a common characteristic of experimentally encountered RFs [2,4,5,8,9]. Further, it is a common characteristic of RFs that emerge in machine learning [10,11]. Although the RFs that we consider do not have the precise shape of measured RFs, for example those of simple cells in V1 [36], they share the key properties of localized, overlapping and broadening RFs that tile the represented space. Indeed, RFs of neurons in hierarchically higher layers are often broader and constructed from those in lower layers [37,38].

### Encoding a 1D stimulus

**Fig 1:**
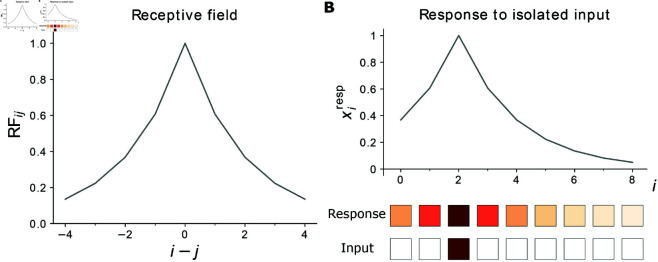
Receptive field and response of 1D network. **(A)** The RF of neuron *i* is peaked at *j* = *i* and decays exponentially with |i−j|. The RF width parameter is here *d* = 2. **(B)** Top: The network response to an isolated unit input, here located at *j*_0_ = 2, has the same shape and amplitude as a neuron’s RF. It peaks at *i* = *j*_0_. Bottom: Input and feature neurons are shown with color-coded activities *r*_*j*_ and *x*_*i*_, respectively. Increasingly dark red color represents higher activity; white squares indicate inactive neurons.

As a concrete, analytically tractable model that illustrates how cooperative coding works and can save synapses, we consider RFs that tile the one-dimensional parameter space of a stimulus (see Fig 1A). An input neuron *j*, j=1,...,N, signals the presence and strength of a stimulus with a specific parameter *j* by nonzero activity *r*_*j*_ > 0. The task of the feature layer is to generate a response that is maximal at the preferred stimulus parameter and then decays exponentially the more different the stimulus becomes from the preferred one. This behavior is qualitatively similar to commonly observed tuning curves such as orientation tuning curves or place fields. We further assume that if multiple stimuli are present, the feature layer responses to their different parameters superpose linearly.

As an example, the inputs may be interpreted as a simple model for the representation of the orientation of a bar in the early visual system. *r*_*j*_ > 0 then means that the orientation is within the *j*th bin of the total orientation range $\left[0,180^{\circ }\right]$. The transformation from input to features in our model describes the combination of responses from hierarchically lower visual areas to hierarchically higher ones [39]. As another example, the neurons may model the activity of place cells on a periodic, closed track. The transformation then models the transformation from input neurons with smaller place fields to neurons with larger place fields. Such a transformation may take place from the hippocampal dentate gyrus to the downstream area CA3 [40]. In our model, the input generates a simple encoding of the current location, where input neuron *j* is active if the animal is in the *j*th location.

The desired stationary feature layer activity can be expressed as

$$x_{i}^{resp}=\sum_{j=1}^{N}{RF}_{ij}r_{j},\;\;\;{RF}_{ij}=e^{-\frac{\left|i-j\right|}{d}}=\gamma^{\left|i-j\right|}.$$ (1)

Here *r*_*j*_ is the activity of the *j*th input neuron, γ=exp(−1/d) and *d* defines the width of the RF. We use periodic boundary conditions. For computations with neuron indices, this means that |i−j| means $\min_{n\in \{-1,0,1\}}|i-j+nN|$. There are as many input as feature neurons. We note that, because of the symmetry ${RF}_{ij}={RF}_{ji}$, the vector ${RF}_{k\cdot }$, describing the RF of feature neuron *k*, is the same as ${RF}_{\cdot k}$, the network response when only input neuron *k* is active, compare Fig 1A and 1B. Summarized in a formula, we have ${x_{k}^{resp}|}_{r_{j}=\delta_{jl}}={RF}_{kl}={RF}_{lk}={x_{l}^{resp}|}_{r_{j}=\delta_{jk}}$, where *k* is fixed and *l* variable.

#### Feedforward implementation.

To model the temporal dynamics of the neurons, we choose a standard simple linear rate network model [41,42]. The purely feedforward network that generates the response Eq 1 as stationary state is then given by

$$\tau \dot{x_{i}}(t)=-x_{i}(t)+\sum_{j=1}^{N}W_{ij}^{ff}r_{j}(t),$$ (2)

with $W_{ij}^{ff}={RF}_{ij}$ and a time constant τ. In the stationary state, we have ${\dot{x}}_{i}=0$ for all *i* and thus

$$x_{i}=\sum_{j=1}^{N}W_{ij}^{ff}r_{j}=\sum_{j=1}^{N}{RF}_{ij}r_{j}=x_{i}^{resp}.$$ (3)

This state is asymptotically stable and globally attracting; the flow is a contraction to it. These properties follow immediately from the fact that the system is linear and has a unique fixed point, which is asymptotically stable because all eigenvalues of the matrix specifying the homogeneous differential equation are negative, equal to −1/τ [43,44]. To approximate the network with a characteristic number of feedforward weights that is smaller than *N*, we require synapses $W_{ij}^{ff}=\gamma^{\left|i-j\right|}$ only where |i−j|≤d. This defines the RF size $n_{RF}=2d+1$ as the number of feedforward synapses per neuron needed to implement the RF within a distance *d* around its center.

#### Cooperative implementation.

**Fig 2:**
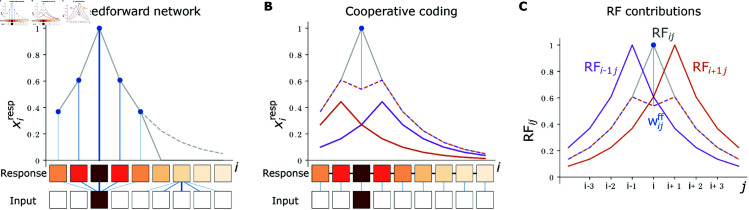
Schematics of feedforward and cooperatively coding networks. **(A)** Top: In the feedforward network, the response $x_{i}^{resp}$ (gray solid curve) to an isolated input is fully generated by the neurons’ feedforward inputs (blue lines and dots, line thickness represents input strength). For the displayed RF width *d* = 2, five neurons receive feedforward input, so that the network response (gray solid curve) represents ≈63% of the summed target response (gray dashed curve). Bottom: Feature and input neuron activities as in Fig 1. Outgoing feedforward synapses from the active input neuron *j* = 2 and incoming feedforward synapses to feature neuron *i* = 6 are shown in blue. **(B)** Top: In the cooperatively coding network model, the network response (gray solid curve) is the sum of feedforward input (blue line and dot) and recurrent input (brown-purple dashed curve). For the displayed case of an isolated input, only one neuron receives feedforward input, which induces a part of the stationary response of the most active feature neuron. The rest of the response and all other responses are induced by recurrent input from neighboring neurons. The total recurrent input that each feature neuron receives is the sum of recurrent input from the right (brown solid curve) and left neighbor (purple solid curve). Bottom: Each feature neuron receives one feedforward synapse (blue lines) and two recurrent synapses (black lines, all recurrent connections are bidirectional). **(C)** The RF of feature neuron *i* (${RF}_{ij}$ for varying *j*, gray solid curve) is the weighted sum (brown-purple dashed curve) of the RFs of its left (${RF}_{i-1\;j}$, purple) and right neighbors (${RF}_{i+1\;j}$, brown) plus a contribution from feedforward input ($W_{ij}^{ff}$, blue line and dot). All shown RFs have width *d* = 2.

The same stationary neuronal responses can be obtained as the steady state of a recurrent network that uses cooperative coding. It requires only three synapses per feature neuron, two recurrent and one feedforward synapse. This network’s dynamics are given by

$$\tau \dot{x_{i}}(t)=-x_{i}(t)+\sum_{j=1}^{N}W_{ij}^{rec}x_{j}(t)+\sum_{j=1}^{N}W_{ij}^{ff}r_{j}(t)$$ (4)

$$=-x_{i}(t)+w^{rec}(x_{i+1}(t)+x_{i-1}(t))+w^{ff}r_{i}(t),$$ (5)

with weights $w^{rec}=\frac{1}{\gamma +\gamma^{-1}}=\frac{\gamma }{1+\gamma^{2}}$ and $w^{ff}=1-2\gamma w^{rec}=\frac{1-\gamma^{2}}{1+\gamma^{2}}$. If the RFs are not narrow (*d* is not small against 1), the two recurrent connections are strong, in the sense that $w^{rec}$ is not small against 1. Thus the network features strong like-to-like excitation and is driven by feedforward input. One can straightforwardly verify that $x_{i}=x_{i}^{resp}$ is indeed a stationary state of the network, by inserting Eq 1 into Eq 5, see Eq S2 in S1 Appendix. The reason for this is ultimately that the desired response of a neuron *i* can be largely generated by summing the responses of the two neurons *i* ± 1 neighboring *i*, see Eq 11 and Fig 2B. This is achieved by the recurrent connections. The missing part is contributed by the feedforward input. This state is asymptotically stable as all real parts of the eigenvalues of the matrix defining the homogeneous system are negative, see Eq S12 in S1 Appendix. For broad RFs (where γ≲1), the recurrent connections are nearly as strong as possible: their sum $2w^{rec}$ is close to 1, the value beyond which the network becomes unstable. The stationary state is also the only stationary state. Since the system is linear, the state is therefore a global attractor as for the feedforward network [43,44]. Thus, for constant input the network forms this stable response pattern.

#### Cooperative coding.

Cooperative coding can be understood as sharing of the information that an individual neuron obtains from external input specifically with those neurons that also need it. This allows to generate most of the neuronal responses from sparse recurrent connectivity. Especially very similarly tuned neurons will project strongly excitatorily onto each other; oppositely tuned neurons would inhibit each other.

As a concrete example, we introduced the networks Eq 5, where it suffices that each neuron receives input from only one input and two feature neurons. Still, each neuron effectively responds to 𝒪(d) input neurons. This is possible because the feature neurons recurrently share their activity, and hence their access to feedforward input, with their neighbors. These in turn share it with their neighbors, thus propagating it through the network. The network response then forms dynamically through the interplay of feedforward input and recurrent interactions.

The coding harnesses the fact that despite very few feedforward and recurrent synapses, poly-synaptic connectivity can still be far-reaching [45,46]: To show this, we consider the formal steady-state solution of Eq 4 for constant input, which provides the network’s desired response if a solution exists. It can be obtained by setting $\dot{x}=0$ and solving for *x* by multiplying with the inverse of $𝟙-W^{rec}$. The solution reads

$$x=(𝟙-W^{rec}{)}^{-1}W^{ff}r.$$ (6)

For the considered excitatory weight matrix with a spectral radius smaller than 1, the inverse exists and can be expanded into a Neumann series, yielding

$$x=\left(𝟙+W^{rec}+(W^{rec}{)}^{2}+\cdots \right)W^{ff}r.$$ (7)

The network response is thus determined by $W^{rec}$ and its higher powers, which reflect the redistribution of feedforward input via poly-synaptic recurrent pathways. As the higher-order terms correspond to longer pathways, they will shape the response at later times. This can be well seen from the approximate, discretized dynamics [47,48]

$$x_{i}((n+1)\tau )\approx \sum_{j=1}^{N}W_{ij}^{rec}x_{j}(n\tau )+\sum_{j=1}^{N}W_{ij}^{ff}r_{j},$$ (8)

which lead to the same steady state as the time-continuous dynamics. The response to a constant input *r* after *n* time constants is

$$x(n\tau )=(𝟙+W^{rec}+\cdots +(W^{rec}{)}^{n-1})W^{ff}r;$$ (9)

higher-power, poly-synaptic terms add to it at successively later times. Viewed differently, a recurrent neural network can be equivalently described by a deep feedforward network that is “unrolled in time” [49], with higher layers generating the results of later computations, through a higher stack of copies of the recurrent weight matrix.

The coding scheme can also be understood as feedforward inputs providing a correction to the response that is mainly constructed from the sparse recurrent input. To clarify this we focus on elementary stationary responses, namely those that are driven by a single unit input from neuron *j*; the input activity is $r_{k}=\delta_{kj}$. Responses to more complicated input patterns are weighted linear sums of such elementary responses. Consider feature neuron *i* and assume that all other neurons already respond correctly. The desired stationary activity of neuron *i* in response to a single unit input from neuron *j* is then ${RF}_{ij}$, while the responses of the other network neurons *k* are ${RF}_{kj}$. Eq 4 with ${\dot{x}}_{i}=0$ implies that ${RF}_{ij}$ is the sum of the RFs of its presynaptic feature neurons and its feedforward connectivity,

$${RF}_{ij}=\sum_{k}W_{ik}^{rec}{RF}_{kj}+W_{ij}^{ff}.$$ (10)

For the specific network Eq 5 we have

$${RF}_{ij}=w^{rec}({RF}_{i-1,j}+{RF}_{i+1,j})+w^{ff}\delta_{ij},$$ (11)

illustrated in Fig 2C. This implies that the desired response of feature neuron *i* is fully generated by recurrent inputs, unless the unit input comes from input neuron *i* and there is also a feedforward contribution. The weighted and summed responses of neuron *i*’s nearest neighbors are thus already very close to neuron *i*’s target response. This is enabled by the specific exponential shape of the RFs. Only for the preferred input of a feature neuron, the response is too low. The neuron corrects for this by recruiting the missing input through a feedforward connection. Such an “explanatory gap” that is left by the recurrent inputs and can be filled by external input is important for cooperative coding, because the output must depend on external input.

#### Spatial demand and metabolic cost.

To compare the efficiency of the introduced implementations, we focus on two cost dimensions: the space needed to implement the network and the metabolic cost of generating the stationary dynamics. As measure for the space needed for the network we take the number of synaptic connections, or, in other words, the L0 norm of the synaptic weight matrix. In the feedforward network Eq 2, it increases linearly with the width *d* of the RF if small responses can be neglected. This holds in particular when using our convention that the number of relevant synapses equals 2*d*  +  1 (see section “Feedforward implementation”). In the recurrent network Eq 5 three synapses per neuron suffice to generate the desired stationary response regardless of the RF size. We show after Eq S18 in S1 Appendix that the recurrent network Eq 5 therefore minimizes the L0 norm.

For the metabolic cost of generating the stationary dynamics, we may focus on the cost of generating the postsynaptic currents, which is proportional to their L1 norm (see S1 Appendix). This is because all other contributors, such as the neuronal activity, are identical between both network architectures. Since in both implementations all modeled synaptic currents are excitatory, the L1 norm of synaptic currents equals the total synaptic current. In the stationary state this current is the same in both implementations, because neurons have the same stationary activity; therefore also the cost is the same. We conclude that the metabolic cost for maintaining the stationary network state is the same in both the feedforward and the recurrent network implementation.

#### Response speed.

**Fig 3:**
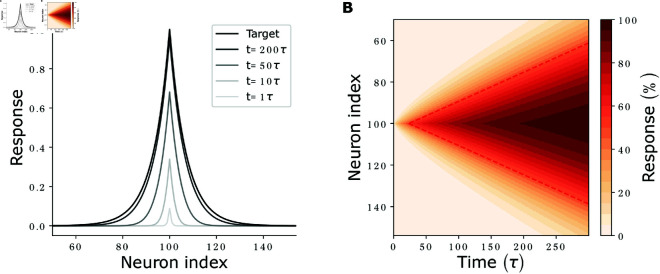
Response formation and activity propagation. **(A)** Network activity at different times (shaded curves) after *r*_100_ has been set from 0 to 1. For long times, network activity approaches the target response (black curve). **(B)** Development of the activity of neurons (y axis) with time (x axis), measured relative to their target activities. The diagonal fronts of equal relative activities indicate propagation of activity with constant propagation speed. The points where neurons reach 50% of their final activity are connected by a red dashed line. Parameters: $w^{rec}=0.5\cdot 1/(1-1/100)$, such that $\tau_{resp}=100\tau$ (see Eq 13), *N* = 200 neurons.

In the feedforward network, activity converges with the intrinsic time constant τ, which we define as its response time. In the cooperatively coding network, the excitatory recurrent connectivity increases the response time: Fig 3 shows the dynamics of the response formation.

**Fig 4:**
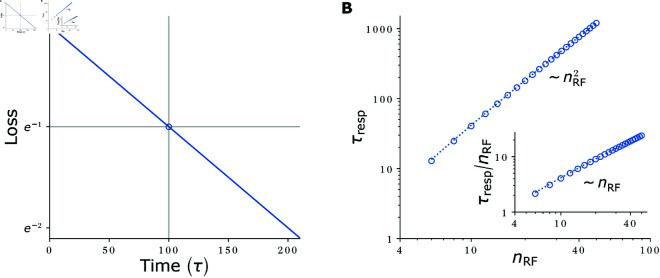
Loss evolution and response speed - synapse number trade-off. **(A)** Exemplary loss evolution for a network with $w^{rec}=0.5\cdot (1-1/100)$ so that $\tau_{resp}=100\tau$. Experimentally, $\tau_{resp}$ is determined as the time (gray vertical line) at which the loss drops to $e^{-1}$ (gray horizontal line, blue open circle). **(B)** Response times $\tau_{resp}$ (circles: simulation results; dotted line: analytical solution Eq 14) for target RFs of different widths $n_{RF}$. Data was created by scanning $n_{RF}$, setting $w_{sum}^{rec}$ to yield an RF of size $n_{RF}$ and determining $\tau_{resp}$ from the loss dynamics.

The network activity splits into independently evolving, orthogonal eigenmodes that approach their steady state values at different speeds, see Eq S8 in S1 Appendix. We use the L1-norm of the deviation of the response from the steady state,

$$L(t)=|x(t)-x^{steady}{|}_{1},$$ (12)

as a loss measure. The linearized loss ($\sum_{i}x_{i}^{\mathrm{steady}}$ − *x*_*i*_ ( *t* ) ) equals a constant offset minus the projection of the activity *x*(*t*) onto the vector of ones (1,⋯,1), which coincides with the slowliest-decaying eigenmode. The linearized loss, as well as the full loss for the constant-zero activity initialization used here, thus decays exponentially. We define the response time $\tau_{resp}$ of the network as the time constant of this decay,

$$\tau_{resp}\equiv \frac{\tau }{1-w_{sum}^{rec}},$$ (13)

(see Fig 4), where $w_{sum}^{rec}=\sum_{j}W_{ij}^{rec}$ is the sum of recurrent weights arriving at (or, equivalently, originating from) a neuron. For generic initial conditions, this provides the time constant of the slowliest-converging activity mode and thus the time constant that dominates the long-term convergence of the full loss; the linearized loss decays with time constant $\tau_{resp}$ during the entire time evolution. $\tau_{resp}$ scales inversely with the difference of the largest eigenvalue of the network, which is equal to $w_{sum}^{rec}$ (cf. Eq S12 in S1 Appendix), from 1. In particular, it depends only on the summed recurrent weights.

Eq 13 holds generally, for networks of the type Eq 4 with purely excitatory circulant recurrent weight matrix and convergent dynamics. We now specialize the result to networks with nearest-neighbor coupling Eq 5 that generate the RFs Eq 1. In these networks, $w_{sum}^{rec}=2w^{rec}$. Inserting $w^{rec}=1/(\gamma +\gamma^{-1})$ and γ=exp(−1/d) relates the response time to the RF width. By approximating $\exp(\pm 1/d)\approx 1\pm 1/d+1/(2d^{2})$ for large *d*, we obtain $w^{rec}\approx 1/(2+1/d^{2})$ and, inserting this into Eq 13,

$$\tau_{resp}\approx \frac{\tau }{1-\frac{2}{2+\frac{1}{d^{2}}}}=(1+2d^{2})\;\tau \approx 2d^{2}\tau \approx \frac{1}{2}n_{RF}^{2}\tau .$$ (14)

In the last part of the equation we used that $n_{RF}=1$  +  2d≈2d for large *d*. Eq 14 shows that wide RFs require long equilibration time. This is because they need strong recurrent weights with a largest eigenvalue close to 1. Further the equation reveals the trade-off between response time and number of employed synapses: The feedforward implementation Eq 2 needs $n_{RF}$ synapses and has a response time τ. The recurrent implementation thus saves $n_{RF}-3\approx n_{RF}$ synapses per feature neuron. Eq 14 shows that the response time increases quadratically in the number of saved synapses, see also Fig 4B.

The quadratic dependence of the response time on *d* reflects that, as the RF becomes wider, not only does activity have to spread further, it also spreads more slowly: this is consistent with the idea that the settling of a neuron depends (indirectly) more on activity propagating back from more distant neurons that settle after it.

#### Faster response with spike frequency adaptation.

For activity to rapidly spread through the network, neurons need to be able to cause a large activity change in their neighbors within a short period of time. To achieve this, they need strong recurrent weights. However, recurrent weights are restricted to $w_{sum}^{rec}<1$ to not cause runaway activity. We now show how spike frequency adaptation (SFA) can help ease this conflict and speed up network dynamics. SFA is typical for excitatory principal neurons and induces a reduction of their response to constant inputs in the long run [42,50,51].

We model SFA through a negative-feedback adaptation current *u*(*t*), which is triggered by neuronal activity *x*(*t*) and characterized by its scale $a_{SFA}$ and time constant $\tau_{SFA}$,

$$\tau {\dot{x}}_{i}(t)=-x_{i}(t)+\sum_{j=1}^{N}W_{ij}^{rec}x_{j}(t)+\sum_{j=1}^{N}W_{ij}^{ff}r_{j}(t)-a_{SFA}u_{i}(t),$$ (15)

$$\tau_{SFA}{\dot{u}}_{i}(t)=-u_{i}(t)+x_{i}(t).$$ (16)

This model is a slightly simplified version of that in [52] and the same as in [53,54]. Setting ${\dot{x}}_{i}(t)=0$ and ${\dot{u}}_{i}(t)=0$ yields the steady state. We see immediately that it implies $u_{i}=x_{i}$. Inserting this into Eq 15 shows that in the stationary state the spike frequency adaptation results in a stronger leak current, $-(1+a_{SFA})x_{i}$. Dividing by $1+a_{SFA}$ yields

$$0=-x_{i}+\sum_{j=1}^{N}\frac{W_{ij}^{rec}}{1+a_{SFA}}x_{j}+\sum_{j=1}^{N}\frac{W_{ij}^{ff}}{1+a_{SFA}}r_{j}.$$ (17)

Consequently, in order to implement the same response as a network without SFA ($a_{SFA}\;=\;0$, cf. Eq 4), the recurrent and feedforward weights have to be scaled up by a factor of $1+a_{SFA}$. The additional excitatory synaptic input compensates in the steady state the added inhibitory adaptation current.

To understand the network dynamics, it is instructive to consider the limit $\tau_{SFA}\to 0$ where $u_{i}(t)\to x_{i}(t)$ as in the steady state. Inserting this into Eq 15 and again dividing by 1  +  $a_{SFA}$ yields an equation equivalent to Eq 4 with smaller neuronal time constant and smaller weights,

$$\frac{\tau }{1+a_{SFA}}{\dot{x}}_{i}(t)\overset{\tau_{SFA}\to 0}{=}-x_{i}(t)+\sum_{j=1}^{N}\frac{W_{ij}^{rec}}{1+a_{SFA}}x_{j}(t)+\sum_{j=1}^{N}\frac{W_{ij}^{ff}}{1+a_{SFA}}r_{j}(t).$$ (18)

We see that a network with arbitrarily fast SFA and appropriately upscaled weights has the same dynamics as a network without SFA, but with its time constant reduced by $1+a_{SFA}$. This factor only depends on $a_{SFA}$ and is independent of the RF width that the network implements. We might thus expect that introducing SFA with a given $a_{SFA}$ and small $\tau_{SFA}$ causes a constant speedup, but still results in a quadratic dependence of $\tau_{resp}^{SFA}$ on $n_{RF}$ (see Eq 14).

There is, however, an additional possibility: SFA might yield faster dynamics for finite, nonzero $\tau_{SFA}$. This is because then *u*_*i*_(*t*) lags behind *x*_*i*_(*t*), which creates a temporal “window of opportunity”. Within this window, the up-scaled weights can mediate strong interactions that are not yet cancelled by the retarded adaptation currents of the receiving neurons. In our networks, this leads to the following concept to exploit SFA: During the initial response phase, strong weights should cause a fast response while SFA keeps the steady state before and after an input change at the desired activity values as well as dynamically stable. In particular, the modified recurrent synaptic weights may then be (and to optimally exploit SFA: should be) so strong that without the SFA current the network dynamics are unstable.

To incorporate SFA in a cooperatively coding network, we modify the weights in Eq 5 as described above and add the SFA current. For the neuron activities, this yields the dynamical equation

$$\tau \dot{x_{i}}(t)=-x_{i}(t)+(1+a_{SFA})w^{rec}(x_{i+1}(t)+x_{i-1}(t))\;\;+(1+a_{SFA})w^{ff}r_{i}(t)-a_{SFA}u_{i}(t),$$ (19)

where the adaptation current obeys Eq 16. We find that the second of the above-described possibilities applies to such networks: Measuring the response time as a function of $\tau_{SFA}$, we observe that it first decreases when increasing $\tau_{SFA}$ from zero and reaches a minimum at a nonzero, optimal value of the SFA time scale (Fig A in S1 Appendix). Increasing $\tau_{SFA}$ further eventually causes diverging activity, because the retarded adaptation current *u*(*t*) becomes so slow that it never compensates the stronger input due to the upscaled weights. We note that also when keeping $\tau_{SFA}$ at a fixed value, there is an optimal nonzero value of the inhibitory feedback strength $a_{SFA}$, which we define to minimize the integrated loss (Fig A in S1 Appendix). Importantly, we find that introducing SFA with finite $\tau_{SFA}$ and optimal $a_{SFA}$ improves the scaling of $\tau_{resp}^{SFA}$ with $n_{RF}$ from quadratic as without or with arbitrarily fast SFA to linear. The accelerating effect is a form of “balanced amplification” [21], where the matrix governing a dynamical system is non-normal, featuring a hidden feedforward structure from difference modes (here: high neuronal activity but still low adaptation currents) to sum modes (here: high neuronal activity and adaptation currents).

We now further analyze the balanced amplification [21] in cooperative coding networks with SFA, by reducing the dynamics Eq 19 to effective single neuron dynamics with feedback. For this we assume that all neuronal activities receive the same inputs *r*_*i*_(*t*) = *r*(*t*) and follow the same time course *x*_*i*_(*t*) = *x*(*t*), so that we can replace $x_{i-1}(t)+x_{i+1}(t)=2x(t)$. We note that we thereby study the eigenmode related to the L1 loss, see Eq S13 in Appendix S7. The resulting effective single neuron dynamics read

$$(\begin{array}{c} \dot{x}(t)\\ \dot{u}(t) \end{array})=(\begin{array}{cc} \frac{2(1+a_{SFA})w^{rec}-1}{\tau } & -\frac{a_{SFA}}{\tau }\\ \frac{1}{\tau_{SFA}} & -\frac{1}{\tau_{SFA}} \end{array})(\begin{array}{c} x(t)\\ u(t) \end{array})+(1+a_{SFA})w^{ff}(\begin{array}{c} r(t)\\ 0 \end{array}).$$ (20)

A complex Schur decomposition of the matrix defining the effective neuron’s intrinsic 2D linear dynamics (homogeneous part of Eq 20) reveals a strong feedforward coupling from a difference mode (real parts of the eigenvector components have opposite sign) to a sum mode (real parts of the eigenvector components have the same sign). In our numerical evaluations we consider networks where the inhibitory feedback strength $a_{SFA}$ is optimized such that the integrated loss is minimal. For these networks 2(1  +  $a_{SFA})w^{rec}-1$ is positive (as the network is unstable without adaptation); furthermore, both the sum and difference mode are oscillatory (as oscillations help to reduce the integrated error), i.e., the matrix in Eq 20 has complex eigenvalues. This is similar to the two-population networks in Ref [21], which, however, have mostly real, non-positive eigenvalues and thus non-oscillatory modes without Hebbian amplification.

Concerning the use of resources, SFA does not require additional synaptic connections, so the spatial demand of the cooperatively coding network is the same as in the original model Eq 5. The increased weights, however, lead to stronger synaptic currents. Together with the added adaptation currents, this increases the energetic cost of maintaining the stationary state.

### Balanced networks

The networks we studied so far had only excitatory synapses, while biological neural networks also have recurrent inhibition, which balances the excitation [32,55]. These are likely required for a range of reasons, such as ensuring network stability and maintaining irregular spiking activity [56–60]. Given their existence, we here show how inhibition can be used to speed up the network response, in an architecture that still relies on few synapses.

Experiments show that individual excitatory and inhibitory currents can be much larger than their sum and precisely temporally balanced with a lag smaller than the neuronal time constant [61,62]. Further, many inhibitory neurons are rather sharply tuned [62–64], sometimes similarly sharply as excitatory ones. To incorporate inhibition consistent with these findings, we add inhibitory neurons to the existing network of excitatory feature neurons. Specifically, we assume that there are as many inhibitory neurons as feature neurons and that each inhibitory neuron follows the activity of one feature neuron with a small delay, $\tau_{lag}$, such that we do not need to introduce a separate dynamical equation for it. Eq 4 thus becomes

$$\tau {\dot{x}}_{i}(t)=-x_{i}(t)+\sum_{j=1}^{N}W_{ij}^{rec,E}x_{j}(t)+\sum_{j=1}^{N}W_{ij}^{rec,I}x_{j}^{\text{I}}(t)+\sum_{j=1}^{N}W_{ij}^{ff}r_{j}(t)$$ (21)

$$=-x_{i}(t)+\sum_{j=1}^{N}W_{ij}^{rec,E}x_{j}(t)+\sum_{j=1}^{N}W_{ij}^{rec,I}x_{j}(t-\tau_{lag})+\sum_{j=1}^{N}W_{ij}^{ff}r_{j}(t),$$ (22)

where $x_{i}^{I}(t)$ is the inhibitory activity, which equals the delayed excitatory feature neuron activity *x*_*i*_(*t* − $\tau_{lag})$. $W_{ij}^{rec,I}\leq 0$ is the coupling from inhibitory neuron *j* to feature neuron *i*. We note that an alternative, more common choice is to model inhibitory activity as low-pass filtered version of excitatory activity, $\tau_{I}{\dot{x}}_{j}^{I}(t)=-x_{j}^{I}(t)$  +  $x_{j}^{E}(t)$ with $\tau_{I}=\tau_{lag}$. We exemplarily checked that this leads to qualitatively similar results (Fig D in S1 Appendix).

We now introduce the state change of feature neuron *i* between $t-\tau_{lag}$ and *t*,

$$\Delta x_{i}(t)=x_{i}(t)-x_{i}(t-\tau_{lag}).$$ (23)

To rewrite the network dynamics in terms of a net interaction and a balanced interaction, we define the new weights

$$W_{ij}^{rec,net}=W_{ij}^{rec,E}+W_{ij}^{rec,I},$$ (24)

$$W_{ij}^{rec,bal}=-W_{ij}^{rec,I},$$ (25)

which we call net and balanced weights, respectively. The balanced weights $W_{ij}^{rec,bal}\geq 0$ describe the part of recurrent excitation that is in the stationary regime balanced (canceled) by inhibition; the net weights describe the unbalanced remainder of the recurrent interaction, which could in principle also be inhibitory. These definitions allow to rewrite Eq 22 as

$$\tau {\dot{x}}_{i}(t)=-x_{i}(t)+\sum_{j=1}^{N}W_{ij}^{rec,net}x_{j}(t)+\sum_{j=1}^{N}W_{ij}^{rec,bal}\Delta x_{j}(t)+\sum_{j=1}^{N}W_{ij}^{ff}r_{j}(t).$$ (26)

We now insert the values of the cooperatively coding network Eq 5 and further assume that the inhibitory neurons inhibit and balance the same sets of neurons that their driving feature neurons excited, i.e., $W_{ij}^{rec,E}=w^{rec,E}\left(\delta_{i+1,j}+\delta_{i-1,j}\right)$ and $W_{ij}^{rec,I}=w^{rec,I}\left(\delta_{i+1,j}+\delta_{i-1,j}\right).$ Eq 22 then becomes

τx˙i(t)=−xi(t)+wrec,E(xi+1(t)+xi−1(t))=−xi(t) +wrec,I(xi+1(t−τlag)+xi−1(t−τlag))+wffri(t). (27)

Further, defining the net weights $w^{rec,net}=w^{rec,E}+w^{rec,I}$ and the balanced weights $w^{rec,bal}=-w^{rec,I}$, Eq 27 becomes

$$\tau {\dot{x}}_{i}(t)=-x_{i}(t)+w^{rec,net}(x_{i+1}(t)+x_{i-1}(t))\;\;+w^{rec,bal}(\Delta x_{i+1}(t)+\Delta x_{i-1}(t))+w^{ff}r_{i}(t).$$ (28)

**Fig 5:**
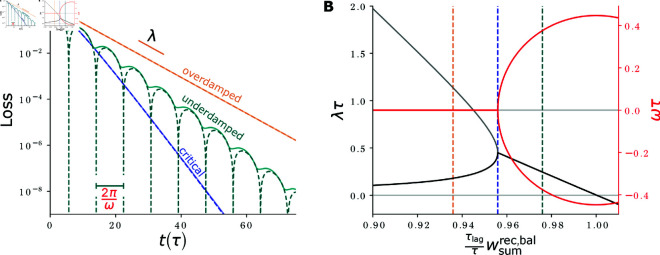
Loss evolution for different strengths of EI-balance. **(A)** Loss evolution (dashed: analytical approximation (cf. Eq S30 and S41 in S1 Appendix, partly occluded; solid: network simulation) for balance strengths that are slightly weaker (orange), equal (blue) or slightly stronger (teal) than the critical balance, on a logarithmic scale. The slope of the decay is given by λ (see (B)), explicitly highlighted for the overdamped dynamics. The oscillation period of the underdamped dynamics is $T_{osci}=2\pi /\omega$. In case of oscillations, the analytic approximation briefly reaches zero loss once in a period (sharp dips in dashed curve). In the network simulation there is also a pronounced oscillation, but there always remains a finite error. **(B)** Real part (decay rate λ, black/gray) and imaginary part (oscillation frequency ω times ±1, red) of the complex frequency of the exponential loss evolution, scaled by τ. For weak EI-balance, measured by $w_{sum}^{rec,bal}$, there are two exponentially decaying modes (λ, black and gray curve). At the critical balance $w_{sum,c}^{rec,bal}$ (blue dashed vertical line), there is only a single decay rate and no oscillation; the decay rate (in the overdamped case: of the relevant slower-decaying mode) is maximized. For stronger balance, network activity begins to oscillate (nonzero ω, red), and diverges once λ becomes negative. This happens approximately at $\frac{\tau_{lag}}{\tau }w_{sum}^{rec,bal}\approx 1+\frac{\tau_{lag}}{3\tau_{resp}}$, which is slightly larger than 1 because of the stabilizing effect of the contracting dynamics of the unbalanced network. Dashed vertical lines show the balance strengths scaled by $\tau_{lag}/\tau$ for the curves in (A) ($(\tau_{lag}/\tau )w_{sum,c}^{rec,bal}-0.02,(\tau_{lag}/\tau )w_{sum,c}^{rec,bal},(\tau_{lag}/\tau )w_{sum,c}^{rec,bal}+0.02$). Parameters: $w_{sum}^{rec,net}=0.99$, τ=1, $\tau_{lag}=0.1$, and *N* = 200 for the network simulation.

The balanced interaction term (proportional to $w^{rec,bal}$) describes the combined effect of delayed inhibition and immediate excitation of the same strength. It depends on $\Delta x_{i\pm 1}(t)$ and therefore only acts when there are activity changes during the preceding brief E-I lag. Any change $\delta x_{j}$ in the activity of neuron *j* causes postsynaptic activity changes in neuron *i* that integrate to $\frac{\tau_{lag}}{\tau }W_{ij}^{rec,bal}\;\delta x_{j}$ (see Eq S21 in S1 Appendix). Once $\frac{\tau_{lag}}{\tau }w_{sum}^{rec,bal}>1$, where $w_{sum}^{rec,bal}=\sum_{i}W_{ij}^{rec,bal}$, an activity change δx in one neuron causes directly further activity changes that are, integrated over time and neurons, larger than δx. Correspondingly, and taking into account the stabilizing contracting dynamics due to the net interactions, the network dynamics become unstable once $\frac{\tau_{lag}}{\tau }w_{sum}^{rec,bal}$ becomes slightly larger than 1, Fig 5.

Henceforth we consider $w^{rec,net}$ and $w^{rec,bal}$ to be the independent variables. This means, in particular, that increasing the balanced weights, or equivalently the strength of the inhibitory weights, implies a concurrent increase of the excitatory weights to keep the net weights invariant. To connect Eq 28 to our previous, unbalanced network Eq 5, we set

$$w^{rec,net}=w^{rec}.$$ (29)

The dynamical equations then agree if $w^{rec,bal}=0$ or $\Delta x_{i}(t)=0$. The latter is satisfied in the steady state. The steady state is thus independent of the strength of the EI-balance, which is given by $w^{rec,bal}$. In particular, the steady state is the same as in Eq 5 (which is the special case of $w^{rec,bal}=0$). The stability of the steady state, however, depends on $w^{rec,bal}$. We may thus think of $w^{rec,net}$ as defining the RF, and of $w^{rec,bal}$ as affecting the dynamics by modulating the EI-balance. During the build-up of the response strong excitation ramps up slightly before the balancing inhibition. For networks with large $w^{rec,net}$, the analytical solution of Eq 28 and our stability analysis (see Sec. ‘Response Speed in the balanced network below and Fig B in S1 Appendix) show that throughout this window of opportunity excitation may be up to approximately $\tau /\tau_{lag}$  +  1 times larger than the net interaction without destabilizing the network. This strong interaction allows a much quicker propagation of activity, convergence to the steady state, and decay of the loss function.

#### Spatial demand and metabolic cost in the balanced network.

Compared to the unbalanced network, the balanced network requires three additional synapses per principal feature neuron, one E-to-I and two I-to-E synapses, i.e., a total of six synapses. This is again independent of the RF width, such that for large RFs, the balanced, cooperatively coding network still saves synapses compared to the feedforward network. It requires additional space for the inhibitory neurons, which may, however, be needed for other purposes anyways.

Also the metabolic maintenance cost increases, since there are more neurons. Further, there is an increased metabolic cost to sustain the synaptic currents in the stationary state: In this state, large parts of the excitatory and inhibitory currents cancel to give rise to a net current that equals the one in the purely excitatory recurrent network, see Eq 24 and Eq 29. In the L1 norm of the synaptic currents, the excitatory currents and the absolute inhibitory currents, however, add up. The metabolic cost thus increases by the amount of excitatory and inhibitory currents that cancel each other.

#### Response speed in the balanced network.

Under some additional assumptions, the evolution of the L1-loss Eq 12 can be analytically approximated as the solution of a linear delay differential equation, see Eq S28 in S1 Appendix for details. The resulting dynamics are those of a damped oscillator, see Fig 5A: For weak EI-balance, characterized by small $w^{rec,bal}$, they are “overdamped” in the sense that they are well described by the sum of two exponentials with different decay rates. At a specific intermediate balance, the two decay rates agree and we have “critical damping”. For stronger balance the dynamics are “underdamped” in the sense that the loss behaves as the absolute value of an oscillation with exponentially decaying amplitude. Overly strong balance, and hence for fixed net interactions overly strong excitation, causes divergence of the dynamics (see Fig 5B and Fig Ba in S1 Appendix).

In the overdamped regime, the smaller decay rate is the relevant one, as it dominates the speed of the decay for longer times. The larger decay rate rather describes how quickly faster dynamics, that may be present due to the initial conditions, are suppressed and the dynamics converge to the slower mode. The smaller decay rate increases when the balance approaches its critical strength. The same holds for the single decay rate in the oscillatory regime. At the critical balance the overall decay of the loss is thus fastest, see Fig 5B.

We find analytically that its decay time constant is approximately proportional to the geometric mean of the response time in absence of inhibition and of the inhibitory delay,

$$\tau_{resp}^{bal,c}\approx \sqrt{\frac{\tau_{resp}\tau_{lag}}{2}},$$ (30)

(cf. Eq S55 in S1 Appendix); the superscript “*c*” indicates that the result holds for critical balance.

**Fig 6:**
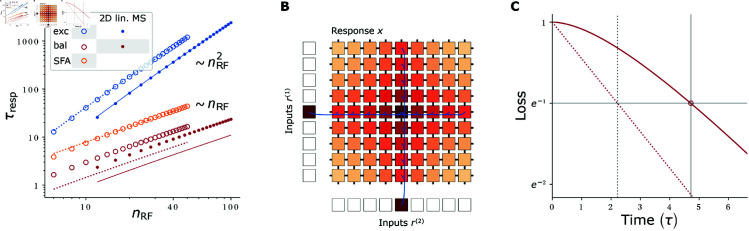
Response speed of networks with inhibition and linear MS. **(A)** Response times for the 1D network and for the 2D linear MS network. The quadratic scaling of $\tau_{resp}$ with $n_{RF}$ for the excitatory networks (blue) can be improved to a linear dependence by introducing balancing, delayed inhibition (red) or SFA (orange). Open (1D network) and filled (MS network) circles display numerical results. Alike-colored dotted (1D network) or continuous (MS network) curves show theoretical estimates (Eqs 14, 31, 37, 38) or, for the SFA network, fit results (monomial fit: $\tau_{resp}^{SFA}(n_{RF})=0.66724{\left(n_{RF}\right)}^{1.07063}$). We use the slowliest-decaying eigenmode to theoretically estimate the response times (see (C) and Eq S46 in S1 Appendix). Since the balanced networks are not initialized in this eigenmode (in contrast to the purely excitatory networks), the numerically measured response times (red markers) lie above the theoretical values (red lines). **(B)** Schematic of a 2D network with linear MS. Feature neurons are arranged on a two-dimensional grid (labeled “Response *x*”). Each receives feedforward input from two arrays of input neurons (labeled “Inputs *r*^(1|2)^”) and four recurrent inputs. Feedforward and recurrent synapses are shown in blue (exemplarily) and black, respectively. Input and feature neuron activities are color-coded. The (linear) network response is the sum of the responses to input one and input two. **(C)** Exemplary loss evolution of a 1D network with lagged inhibition. Due to the temporally constant initialization (*x*_*i*_(0) = 0, $\Delta x_{i}(0)=0$), the network activity (solid red curve) converges initially more slowly than the network’s slowest eigenmode (dotted red line). The experimentally measured response time (continuous vertical gray line) is defined as the time when the loss has decayed by 1/*e* (red open circle, horizontal gray line), see also Fig 4A. It is larger than that of the network’s eigenmode (dotted gray line), which we use as analytical estimate of the response time. We created the data in (A) by scanning $n_{RF}$, setting $w_{sum}^{rec,net}$ to yield an RF of size $n_{RF}$, setting $w_{sum}^{rec,bal}$ to 0 or its critical value, and determining $\tau_{resp}$ or $\tau_{resp}^{bal}$ from the loss dynamics. For the SFA network we set $\tau_{SFA}=\tau$, scanned $a_{SFA}$ and used the value that minimized the temporally integrated loss.

Importantly, this implies that the scaling of the response time with the RF width and size improves compared to the purely excitatory network. This is because $\tau_{resp}^{bal,c}∼\sqrt{\tau_{resp}}$. Inserting Eq 14 into Eq 30 for the 1D network, we obtain

$$\tau_{resp}^{bal,c}\approx \sqrt{\frac{(1+2d^{2})\;\tau \tau_{lag}}{2}}\approx d\sqrt{\tau \tau_{lag}}\approx \frac{1}{2}n_{RF}\sqrt{\tau \tau_{lag}},$$ (31)

which is only linear in the RF width *d* and size $n_{RF}$, instead of quadratic as in the case of $w_{sum}^{rec,bal}=0$, compare Eq 31 with Eq 14 and in Fig 6A the red and blue dotted curves. As a consequence also the speedup gained through the balance, $\tau_{resp}/\tau_{resp}^{bal,c}$, increases for wider RFs.

We finally note that the balanced interactions mediated by $W^{rec,bal}$ can also be thought of as implementing an excitatory transmission of activity changes: an activity change in neuron *j* adds an activity change with the same sign to neuron *i*, because $W^{rec,bal}=-W^{rec,I}$ in Eq 26 is positive. Thus activity changes in different neurons in the network amplify each other. In the limit of small $\tau_{lag}$, the temporal derivative is transmitted, see below Eq S21 in S1 Appendix.

### Linear mixed selectivity

Neurons often respond selectively to more than one stimulus or input feature [65]. This phenomenon is called mixed selectivity (MS). Since our networks are linear, we consider linear mixed selectivity, where neuronal responses are linear functions of multiple stimuli. This is a simplification compared to the nonlinear mixed selectivity that is ubiquitous in the brain [65,66]. Concretely, neurons with activities *x*_*ij*_, i,j=1,⋯,N, are arranged on a two-dimensional grid and respond with equal selectivity to two input features, represented by input neurons $r_{k}^{\left(1\right)}$ and $r_{l}^{\left(2\right)}$ with k,l=1,⋯,N,

$$x_{ij}^{resp}=\sum_{k=1}^{N}{RF}_{ijk}^{\left(1\right)}r_{k}^{\left(1\right)}+\sum_{l=1}^{N}{RF}_{ijl}^{\left(2\right)}r_{l}^{\left(2\right)}.$$ (32)

Due to the linearity in the input representation and in the network, the total response is the sum of the responses to the single input features. We take the grid axes to be aligned with the stimulus dimensions, so that the first index in *x*_*ij*_ determines its response to ${\text{r}}^{\left(1\right)}$ and the second that to ${\text{r}}^{\left(2\right)}$. We model this dependence as the same localized, exponentially decaying shape as for the 1D network (cf. Fig 6B),

$${RF}_{ijk}^{\left(1\right)}=\gamma^{\left|i-k\right|}\;\;\;{RF}_{ijk}^{\left(2\right)}=\gamma^{\left|j-k\right|}.$$ (33)

The desired network response can be generated as the steady state of a recurrent network that is equivalent to the 1D network Eq 5 in each dimension of the 2D grid (see next section),

$$\tau {\dot{x}}_{ij}=-x_{ij}+w^{rec,MS}(x_{i+1,j}+x_{i-1,j}+x_{i,j+1}+x_{i,j-1})\;\;+w^{ff,MS}(r_{i}^{\left(1\right)}+r_{j}^{\left(2\right)}),$$ (34)

with the modified constants $w^{rec,MS}=\frac{w^{rec}}{1+2w^{rec}}=\frac{\gamma }{{\left(1+\gamma \right)}^{2}}$ and $w^{ff,MS}=\frac{w^{ff}}{1+2w^{rec}}=\frac{1-\gamma }{1+\gamma }$. Each neuron receives two external inputs and is connected to its nearest neighbors along each stimulus axis. The network has thus only six synapses per neuron, regardless of the RF width. Also a feedforward network where each dimension of the 2D grid is equal to the 1D network Eq 2 generates the desired response. This implementation requires $n_{RF}^{MS}=2n_{RF}=2(2d+1)$ synapses per neuron, a number that increases linearly with the RF width.

#### Mapping to a 1D system.

In the following, we trace the network dynamics Eq 34 back to those of the 1D system Eq 5. Due to the linearity of Eq 34, network responses again superpose. It thus suffices to study the network in the case where only one input neuron is active: we choose $r_{i}^{\left(1\right)}$, which specifies a property of the first stimulus, to be nonzero. Since the input is independent of *j*, the dynamics Eq 34 are (for initial conditions homogeneous in *j* such as *x*_*ij*_(0) = 0) independent of *j*, $x_{ij}(t)=x_{i}(t)$. The recurrent inputs $w^{rec,MS}(x_{i,j+1}(t)+x_{i,j-1}(t))=2w^{rec,MS}x_{i}(t)$ then simply amount to a modification of the leak current to $-(1-2w^{rec,MS})x_{i}$,

$$\tau {\dot{x}}_{i}=-(1-2w^{rec,MS})x_{i}+w^{ff,MS}r_{i}^{\left(1\right)}+w^{rec,MS}(x_{i+1}+x_{i-1}).$$ (35)

After dividing by $\left(1-2w^{rec,MS}\right)$, the differential equation for *x*_*i*_ becomes

$$a\tau {\dot{x}}_{i}=-x_{i}+aw^{rec,MS}(x_{i+1}+x_{i-1})+aw^{ff,MS}r_{i}^{\left(1\right)},$$ (36)

where we introduced $a={\left(1-2w^{rec,MS}\right)}^{-1}$ for brevity. With the values of the constants $w^{rec,MS}$ and $w^{ff,MS}$ highlighted after Eq 34 this is equivalent to the one-dimensional network dynamics Eq 5 up to a different neuronal time constant aτ instead of τ. (We note that we obtained the modified constants such that this holds. For example, equating the prefactors of the recurrent term in Eq 36 and Eq 5 gives $w^{rec}=aw^{rec,MS}=(1$ − $2w^{rec,MS}{)}^{-1}w^{rec,MS}$, which then can be solved for $w^{rec,MS}$.) As a direct consequence, while the 1D network must have recurrent coupling strength of $w^{rec}<0.5$ for being stable, the 2D MS network must have $w^{rec,MS}<0.25$. This is because in the MS network, a neuron receives direct recurrent input from four nearest neighbors instead of two as in the 1D case.

Eq 36 means that the RF of the MS network, along one axis, has the same shape and width *d* as the equivalent one-dimensional network. In particular, *d* is related to the recurrent weight strength $aw^{rec,MS}$ via $aw^{rec,MS}=w^{rec}=\frac{\gamma }{{\left(1+\gamma \right)}^{2}}$ and γ=exp(−1/d); the two RF components in Eq 33 are the same as the RFs in Eq 1, for example ${RF}_{ijk}^{\left(1\right)}={RF}_{ik}$.

#### Response speed.

From the mapping of the MS to the 1D system, Eq 36, we see that the MS dynamics behave in response to a single input like the 1D dynamics with the neuronal time constant τ enlarged by a factor of *a*. The response time is thus given by Eq 14, but with enlarged neuronal time constant, τ→aτ. For sufficiently large *d*, we have γ≈1 (reflecting the spatially slow RF decay), $w^{rec}\approx 1/2$, $w^{rec,MS}\approx 1/4$ and thus a≈2. The scaling of the response time with the RF width *d* and size $n_{RF}^{MS}$ is thus again quadratic,

$$\tau_{resp}^{MS}\approx (1+2d^{2})\;2\tau \approx 4d^{2}\tau \approx \frac{1}{4}{\left(n_{RF}^{MS}\right)}^{2}\tau .$$ (37)

In the last equation we used $n_{RF}^{MS}=2(2d+1)\approx 4d$. Compared to the 1D case (Eq 14), the response time as a function of *d* is therefore larger by a factor a≈2. In contrast, it is smaller by a factor 1/2 as a function of the RF size, compare Eq 37 with Eq 14 and the blue continuous and dotted curves in Fig 6A. In other words: the trade-off between response time and number of needed synapses improves for sufficiently large RFs by a constant factor of about 1/2 compared to the 1D network. This is because the MS network effectively implements two 1D RFs (Eq 32).

#### Balanced network.

We now incorporate the effect of inhibitory neurons into the MS network. As in the 1D case, we assume that the generated inhibition precisely tracks excitation with a short time delay. We thus add to each recurrent excitatory connection an inhibitory one that is slightly delayed. This results in a delayed differential equation like Eq 28 for the balanced MS network dynamics. The parameters are given by those of the 1D balanced system up to a factor $a=1+w_{sum}^{rec,net}$, like in Eq 36. Further, it is again sufficient to study the response dynamics to a single input, which can be reduced to those of the 1D balanced network Eq 28 with adapted parameters. As in the purely excitatory case, for a fair comparison of response times, we consider MS and 1D networks with the same neuronal time constant τ. The effective time constant of the MS dynamics is then aτ. Therefore the response time of the MS network is given by that of the 1D network Eq 31 with neuronal time constant τ replaced by aτ≈2τ,

$$\tau_{resp}^{bal,c,MS}\approx \sqrt{(1+2d^{2})\;\tau \tau_{lag}}\approx \sqrt{2}d\sqrt{\tau \tau_{lag}}\approx \frac{1}{2\sqrt{2}}n_{RF}^{MS}\sqrt{\tau \tau_{lag}}.$$ (38)

At the critical balance, the response time thus scales again with the square root of the response time of the unbalanced network (Eq 30). Therefore it scales linearly with the RF width *d* and size $n_{RF}^{MS}$. The response time is as a function of the RF size by a factor of about $1/\sqrt{2}$ smaller than that in the 1D case, Eq 31, see Fig 6A, red continuous and dotted curves. In other words, the trade-off between response time and number of required synapses improves by a factor of $1/\sqrt{2}$. This is again because the MS network effectively implements two RFs in the MS case; the RF size doubles for the same width compared to the 1D network.

As for the 1D stimulus, the balanced networks have twice as many neurons and additional synapses: Each (excitatory) feature neuron drives one inhibitory neuron, which mirrors its activity. This inhibitory neuron in turn forms inhibitory synapses to the four nearest neighbors that its presynaptic feature neuron excites. In total, the balanced, cooperatively coding network thus requires eleven synapses per feature neuron, instead of six for the unbalanced network. This is independent of the RF width, such that the balanced, cooperatively coding network saves synapses for sufficiently wide RFs.

#### Higher-dimensional linear MS.

We can straightforwardly extend the introduced scheme to networks that have MS with *P* > 2 stimuli. Neurons are then arranged on a hyper-grid with one grid axis per stimulus dimension, so that ${\text{N}}^{\text{P}}$ feature neurons respond to *PN* input neurons. In the cooperatively coding network, each neuron receives *P* feedforward and 2*P* recurrent inputs, requiring a total of 3*P* synapses per neuron. The feedforward network, in contrast, needs for each stimulus dimension 2*d*  +  1 synapses, in total P(2d  +  1) synapses per neuron. The number of saved synapses thus grows linearly with the number of encoded stimulus dimensions and the RF width.

### Encoding a 2D stimulus

**Fig 7:**
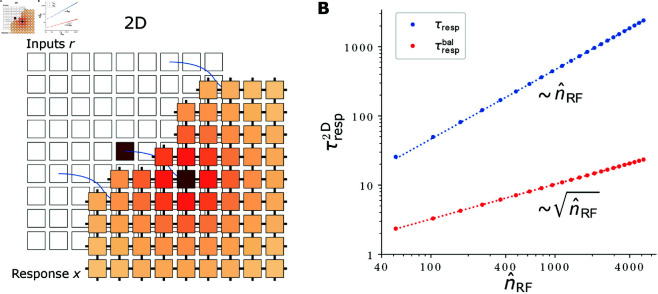
2D network schematic and response times versus RF size. **(A)** Schematic of a two-dimensional network responding to a two-dimensional stimulus. Feature neurons (labeled “Response *x*”) and input neurons (labeled “Inputs *r*”) are arranged on two-dimensional grids. In the cooperatively coding network each feature neuron receives one feedforward and four recurrent inputs; activities and shown connections are color-coded as in Fig 2. **(B)** Response times in the cooperatively coding 2D network increase linearly (without inhibition, blue. Monomial fit: $\tau_{resp}^{2D}=0.46115\;{\left({\hat{n}}_{RF}\right)}^{1.00085}$) or square-root-like (with inhibition, red. Monomial fit: $\tau_{resp}^{bal,2D}=0.31995\;{\left({\hat{n}}_{RF}\right)}^{0.50202}$) with the RF size ${\hat{n}}_{RF}$. Dotted lines represent the monomial fits. Data was created by scanning $w_{sum}^{rec,net}$, setting $w_{sum}^{rec,bal}$ to 0 or its critical value, and determining ${\hat{n}}_{RF}$ and $\tau_{resp}$ or $\tau_{resp}^{bal}$, respectively, from the response curves after network activity converged.

We finally consider the encoding of a two-dimensional stimulus, with both input and feature neurons arranged on a two-dimensional grid, see Fig 7A. Two-dimensional input appears for example in vision [36] or planar navigation tasks [67]. Each feature neuron responds to inputs that are close to its preferred input in both stimulus dimensions. The RFs of neighboring neurons thus overlap and neuronal responses tile the represented stimulus space. A purely excitatory cooperative coding network generating such activity as stationary state is given by

$$\tau {\dot{x}}_{ij}=-x_{ij}+w^{rec,2D}(x_{i+1,j}+x_{i-1,j}+x_{i,j+1}+x_{i,j-1})+w^{ff,2D}r_{ij}.$$ (39)

It has the same recurrent connectivity as the network with linear MS Eq 34, but the feedforward input is arranged on a grid. The activity of feature neuron *ij* in the stationary state is

$$x_{ij}^{steady}=\sum_{kl=1}^{N}{RF}_{ijkl}r_{kl}.$$ (40)

The neuron thus responds to a combination of two input features represented by input neurons *r*_*ij*_ with i,j=1,⋯,N. The RF is explicitly given by (see Eq S62 in S1 Appendix)

$${RF}_{ijkl}\approx c\cdot K_{0}(\gamma^{2D}\rho_{ijkl}),$$ (41)

with $c=\frac{1}{2\pi }\frac{w^{ff,2D}}{w^{rec,2D}}$, $\gamma^{2D}=\sqrt{\frac{1-4w^{rec,2D}}{w^{rec,2D}}}$ and $\rho_{ijkl}=\sqrt{|i-k{|}^{2}+|j-l{|}^{2}}$. *K*_0_ is the zeroth modified Bessel function of the second kind, which decays with distance ρ approximately as $K_{0}(\rho )\approx \frac{\pi }{2}e^{-\rho }/\sqrt{\rho +1/8}$ [68]. The RF is thus approximately radially symmetric. The RF size depends on $w^{rec,2D}$ and the response amplitude also on $w^{ff,2D}$. The network requires 5 synapses per neuron.

We also construct a balanced network by introducing for each excitatory recurrent input a delayed inhibitory one, like in the balanced 1D and MS networks. A balanced implementation with explicit inhibitory neurons requires twice as many neurons and synapses than the purely excitatory network: it requires additionally one inhibitory neuron per principal neuron, one inhibitory synapse for each excitatory recurrent synapse and one synapse from each principal neuron to its corresponding inhibitory neuron.

#### Response speed.

To estimate the dependence of the response speed on the RF size, we first need to appropriately adapt the definition of the RF size, which we introduced after Eq 3. For the one-dimensional network, this definition can be reformulated as follows: we count the number of synapses that are necessary to generate the largest (around the center) RF responses such that these responses summed together amount to a fraction of about $1-e^{-1}\approx 63\%$ of the summed non-truncated RF. Accordingly, for the 2D network at hand we define the RF size as the number of feedforward synapses that are necessary to implement the largest RF entries, such that together they account for a fraction of approximately 63% of the summed nontruncated RF. We denote the so-defined RF sizes by ${\hat{n}}_{RF}$.

As for the 1D and linear MS networks, the response time with or without lagged inhibition depends only on the summed excitatory weights or on the summed net and inhibitory weights. It is thus given by Eq 13 or by Eq 30 in terms of $w_{sum}^{rec}=4w^{rec,2D}$ or in terms of the alike obtained $w_{sum}^{rec,net}$ and $w_{sum}^{rec,bal}$. Fig 7B shows that the scaling of the response time with the RF size is linear for unbalanced and square-root-like for balanced networks. We give a geometric argument for this general scaling in the next paragraph. The scaling is more economical than for the 1D and 2D linear MS networks, cf. Fig 6.

#### Multi-dimensional stimuli.

For P≥2-dimensional stimuli, the fact that activity simultaneously propagates along all dimensions suggests that the scaling of the response time with *d*, the characteristic RF width along one of the dimensions, does not change with the number of dimensions. However, the RF size $n_{RF}$, the number of synapses needed in a purely feedforward implementation, can be assumed to scale as $n_{RF}∼(2d$  +  1)^*P*^ (number of neurons in a cube with 2*d*  +  1 neurons at each edge), with a prefactor that depends on geometry. This reasoning suggests that the response time of networks encoding higher-dimensional stimuli scales like $\tau_{resp}∼n_{RF}^{2/P}$ (spreading time of activity along one of the dimensions, since spreads in all dimensions happen simultaneously) and $\tau_{resp}^{bal}∼n_{RF}^{1/P}$ (spreading time of activity along one dimension for the balanced network), which we verified for P=1,2. For higher-dimensional stimuli, the trade-off between response time and saved synapses would thus become highly beneficial: the response time $\tau_{resp}$ or $\tau_{resp}^{bal}$ would grow only slowly with the number of saved synapses due to the strongly sublinear relationship with $n_{RF}$ for larger *P*.

### Cooperative coding in spiking neural networks

#### Excitatory networks.

**Fig 8:**
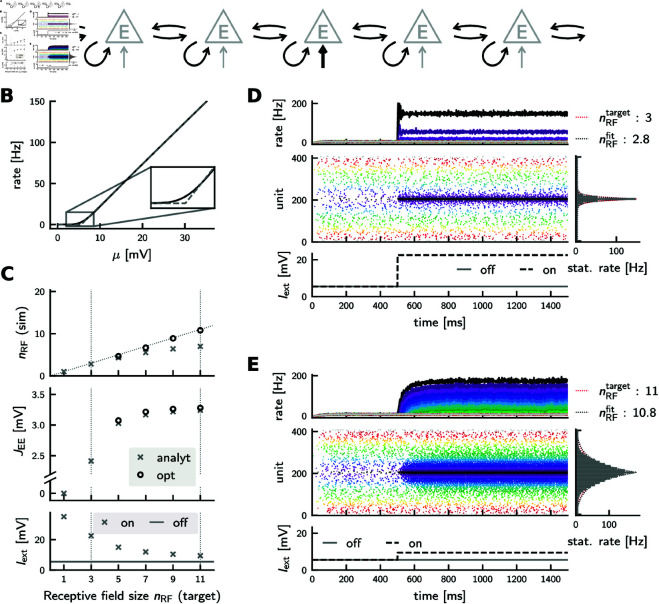
Cooperative coding in an excitatory spiking network of leaky integrate-and-fire neurons. **(A)** Network wiring diagram. Connections are marked in black if their strength depends on RF size, and in gray otherwise. **(B)** Single neuron transfer function (black solid) and threshold-linear fit (gray dashed). Inset highlights onset nonlinearity. **(C)** Top: Simulated vs. target RF size. Light gray crosses: networks with analytically computed weights; black circles: networks with numerically optimized weights. Middle: excitatory synaptic weights (normalized for arbitrary indegree: the voltage increase in response to one spike is given by $w_{EE}=J_{EE}/K_{EE}$) . Bottom: Feedforward input to stimulated population (*on*) and to others (*off*), used both for networks with analytically or numerically computed weights. Left and right dashed vertical lines mark example simulation shown in (D) and (E). The target RF peak rate was set here to $x_{max}=150$ Hz. **(D)** Example simulation for target RF size $n_{RF}=3$. Top: Binned average firing rates of 41 feature populations. Middle: Raster plot showing spikes of 10 exemplary neurons from each feature population. Color codes distance from the stimulated population (population index 21, the population includes neurons 200-209 shown in black). Bottom: Feedforward stimulation. Right: Stationary population rates (gray), exponential fit (black dashed), and target rate profile (red). **(E)** Example simulation for target RF size $n_{RF}=11$. All panels are as in (D).

So far, we implemented and analyzed cooperative coding in linear rate networks. We now show that the main results concerning synaptic savings as well as scaling of response speed with and without balanced amplification also apply to cooperatively coding spiking neural networks. We demonstrate this for a one-dimensional feature layer with periodic boundary conditions (Fig 8A, cf. Fig 1). Each feature (rate) neuron becomes a feature population composed of multiple spiking neurons. Specifically, we use leaky integrate-and-fire (LIF) neurons (see Methods, Eq 47). Synaptic coupling is sparse and random; couplings are restricted to neurons of the same and neighboring feature populations (Fig 8A). The feedforward input is modeled as a constant mean drive, which is the same for all neurons of a given feature population. Every neuron also receives independent Gaussian white noise mimicking balanced background activity. For a moderate amount of noise and when omitting the absolute refractory period, the single neuron transfer function becomes approximately threshold-linear (Fig 8B). Since we require threshold-linearity only for the network’s operating regime between 0 Hz and a peak rate *x*_max_, small absolute refractory periods can be incorporated without deviating too much from threshold-linearity.

We use an analytical approximation to construct appropriate networks: We first approximate synaptic inputs with a diffusion approximation. Using the threshold-linear approximation of the single neuron transfer function, we then derive a self-consistency condition for the stationary feature population rates (Eq S64 in S1 Appendix). This yields for any target RF with size $n_{RF}$ and peak rate $x_{max}$ the feedforward stimulation $I_{ext}^{on}$ and the recurrent synaptic weight $J_{EE}$ for which the network is expected to settle into the target rates (Eq S74 – S77 in S1 Appendix).

For small RF sizes, the analytically obtained stimulation and weight sizes directly yield networks that generate the desired RFs (Fig 8C, gray crosses). Larger RFs depend more sensitively on the recurrent synaptic coupling strength, so we further optimize $J_{EE}$ numerically to obtain the desired RFs (Fig 8C, black circles; see also Discussion and Fig G in S1 Appendix). We decided not to co-tune the feedforward strength $I_{ext}^{on}$ since the simpler, one-dimensional optimization of only $J_{EE}$ already yields satisfactory matches to the target fields.

Analogously to the rate model, the spiking network can exhibit cooperative coding, i.e., the neurons can have wide RFs despite narrow feedforward and limited recurrent connectivity. The stationary spiking activity in our numerically simulated networks is asynchronous (Fig 8D, 8E). For an isolated, constant input to one feature population (black arrow in Fig 8A), the stationary firing rates, averaged within feature populations and over time (Fig 8D, 8E, right panel, gray bars), decay with distance from the stimulated population, as desired. The slight deviations from the exponentially decaying target profile (Fig 8D, 8E, right panel, red dotted line) likely arise from the onset nonlinearity of the transfer function (see inset in Fig 8B).

For the simulations shown in Fig 8, we fixed the indegree of the recurrent synaptic coupling and used relatively large feature populations to stabilize the dynamics and facilitate the estimation of stationary rates and response speeds. We confirmed in exemplary simulations that cooperative coding is still possible with smaller (e.g. *N*_*E*_ = 500) feature populations with random Erdös-Rényi connectivity and under Poisson spiking input (Fig H in S1 Appendix).

#### Number of synaptic connections.

As in the rate model, cooperative coding can save synapses compared to a feedforward network: With pure feedforward coding, every feature neuron needs to receive input from as many input populations as the RF is large ($∼n_{RF}$, cf. Fig 2A) — as well as recurrent input from its peers, if we include within-population coupling for comparability with the cooperatively coding network. On average this yields

$$N_{\text{FF}}=n_{RF}K_{FF}+K_{EE}^{in}$$ (42)

synaptic inputs per feature neuron, where $K_{FF}$ denotes the average feedforward indegree from an input to a feature population and $K_{EE}^{in}$ denotes the average indegree of recurrent coupling within a feature population. In the cooperatively coding network, the number of synaptic inputs is independent of RF size: Every feature population receives input from only one input population, from itself, and from its two neighboring feature populations (Fig 2B, Fig 8A):

$$N_{\text{CC}}=K_{FF}+K_{EE}^{in}+2K_{EE}^{cross},$$ (43)

where $K_{EE}^{cross}$ denotes the average indegree for connections across two neighboring feature populations. We note that synaptic coupling within feature populations ($K_{EE}^{in}>0$) is not required for either feedforward or cooperative coding, but was included here for biological plausibility. In biological neural networks, it might be used for cooperative coding between identically tuned neurons to save further feedforward synapses. In our simplest model we assume that all indegrees are equal, $K_{FF}=K_{EE}^{in/cross}=:K$. In this case, the number of synapses per feature neuron is smaller in the cooperative coding network for all RF sizes larger than three:

$$N_{\text{CC}}=4\;K<(1+n_{RF})K=N_{\text{FF}}\;\text{for }n_{RF}>3\;.$$ (44)

The total number of synapses in the network depends also on the size *N*_*E*_ of the *N*_*F*_ feature populations. We have $N_{\text{FF}}^{total}=N_{F}N_{E}N_{\text{FF}}$ and $N_{\text{CC}}^{total}=N_{F}N_{E}N_{\text{CC}}$ synapses for feedforward and cooperative coding architectures, respectively. Furthermore, if we assume a fixed connection probability $p_{EE}$ between neurons of connected feature populations, $K_{EE}^{in/cross}$ increases with *N*_*E*_. The total number of synapses in the network then becomes

**Fig 9:**
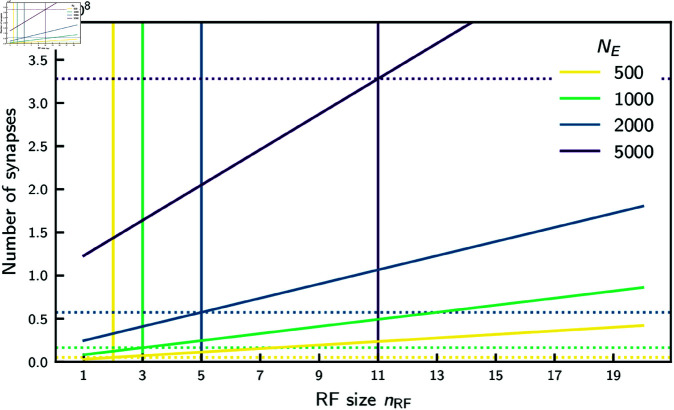
Number of synaptic connections for the feedforward and cooperative coding architecture depending on RF size, for different sizes of feature populations. Total number of synapses in a network of *N*_*F*_ = 41 feature populations as a function of RF size $n_{RF}$ and number of neurons *N*_*E*_ per feature population (color-coded). In the feedforward network the number of synapses $N_{\text{FF}}^{total}$ (solid lines) depends linearly on RF size with a slope proportional to *N*_*E*_. In the cooperative network the number of synapses $N_{\text{CC}}^{total}$ (dotted lines) is independent of RF size but increases quadratically with *N*_*E*_. Vertical lines mark the minimal RF size for which the cooperative network saves synapses compared to the feedforward network ($N_{\text{CC}}^{total}\leq N_{\text{FF}}^{total}$). Synapse numbers are shown here for $K_{FF}=100$ and $p_{EE}=0.1$.

NFFtotal=NFNENFF=nRFNFKFFNE+3pEENFNE2, (45)

$$N_{\text{CC}}^{total}=N_{F}N_{E}N_{\text{CC}}=N_{F}K_{FF}N_{E}+3p_{EE}N_{F}N_{E}^{2}.$$ (46)

(If, instead, the indegree of each neuron stayed constant, i.e., independent of *N*_*E*_, the scalings would only be linear in *N*_*E*_.) We observe that for larger feature populations synaptic savings happen from larger field sizes on, Fig 9. The cooperative scheme relies on averaging recurrent synaptic input and therefore requires a certain minimal population size *N*_*E*_. Our estimate Fig 9 suggests that a cooperative spiking network with 1000 neurons per feature population may save synapses for RFs larger than ∼3.

#### Response speed and balanced networks.

**Fig 10:**
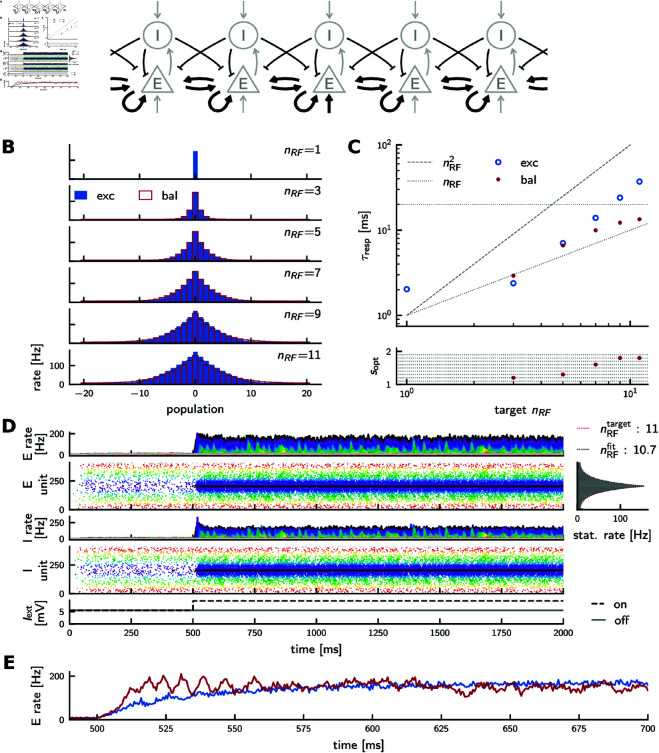
Response times of cooperatively coding spiking neural networks. **(A)** Network wiring diagram of the balanced excitatory-inhibitory spiking network. Connections are marked in black if their strength depends on RF size, and in gray otherwise. **(B)** Stationary rate profiles of excitatory (blue) and balanced networks (red) in response to isolated inputs match. There is no balanced network simulation shown for $n_{RF}=1$, since there is no recurrent coupling between or within populations in that case. **(C)** Top: Response times. Blue: purely excitatory network (cf. Fig 8). Red: balanced excitatory-inhibitory network. Dashed horizontal line: membrane time constant. Quadratic and linear scaling is indicated by the dashed and dotted lines, respectively. Bottom: scaling factor *s* used to scale up the excitatory synaptic coupling strength $J_{EE}$ in the balanced network. **(D)** Activity of a balanced network with RF size 11. Panels as in Fig 8D, 8E; top: excitatory, bottom: inhibitory populations. **(E)** Direct comparison of the rate of the stimulated population (index 21) around stimulus onset in the purely excitatory network (blue, cf. black trace in Fig 8E), and in the balanced network (red, cf. black trace in panel D, top). Rates are shown for a finer binsize of 0.5 ms.

In the purely excitatory spiking network, the response time increases approximately quadratically with RF size for larger RFs (Fig 10C, blue). In rate networks, we observed a speedup and even an improved scaling of the response time for balanced networks. To investigate whether this also occurs in spiking networks, we implement a spiking version of the balanced rate network described in Eq 21 (see Methods, Eq 48 and Eq 49, and Fig 10A). Taking advantage of the approximately threshold-linear transfer functions of single neurons, we tune the synaptic strength from excitatory to inhibitory feature populations such that the inhibitory populations fire at approximately the same stationary rate as their excitatory counterparts, analogous to our rate models (Eq S78 in S1 Appendix). To construct a balanced network, we increase the excitatory recurrent coupling $J_{EE}$ by a factor *s* > 1, and tune the inhibitory-to-excitatory projections such that the balanced network exhibits an RF of approximately the same size as the excitatory reference network (Fig 10B, Eq S81 in S1 Appendix). For each target RF size $n_{RF}$ we broadly grid-search for the scaling factor *s* that yields the shortest rate response time (Fig 10C). For such optimal amplification $s_{opt}$, the scaling of the response speed with respect to RF size improves from quadratic to linear as in the rate model (cf. Fig 6). As expected from the theory (Eq S43 in S1 Appendix), the optimal scaling factor increases with RF size (Fig 10C, bottom). The speedup of the response is likely due to balanced amplification as in the rate network.

We note that, depending on the nature and strength of the noise, spiking neurons can exhibit fast rate responses with transient onmodulated oscillations to step current input [69–71]. We observe such oscillations in our purely excitatory spiking networks for small RF sizes (where we have strong feedforward input), e.g. Fig 8D. This is in contrast to our purely excitatory rate networks. We also find transient oscillatory rate modulations in our spiking EI networks, e.g. Fig 10E.

Given their often rather sharp tuning [62–64], it is conceivable that not only excitatory, but also inhibitory feature populations receive tuned feedforward input. We thus also construct a network where the feedforward input to both subpopulations is the same (see Eq S101 in S1 Appendix). Inspired by “Model A” of [72] we furthermore use the same connectivity and biophysical parameters for both excitatory and inhibitiory subpopulations, such that they have the same stationary rates. We fix the coupling strengths within EI feature populations and only tune the across-population coupling and feedforward input to achieve the desired RF size (Eq S108 and S110 in S1 Appendix). Interestingly, in this network it seems that the balanced amplification mechanism cannot come into effect: In the terminology of [21], both feedforward and recurrent inputs recruit only sum, not difference modes. Nevertheless, we find that the response speed of this network is enhanced compared to an excitatory-only network with the same excitatory within-population coupling strength (Eq S85 and Fig I in S1 Appendix). This may be a consequence of the fact that embedding neurons in balanced EI nodes slightly flattens their transfer function (Fig Ib in S1 Appendix). Thus, the across-population coupling strength required for a certain RF size to emerge is larger than in the purely excitatory network (Fig Ic in S1 Appendix). This increase in cross-population coupling may induce the speed-up of signal propagation.

## Discussion

**Cooperative coding.** In this work, we have studied networks that encode continuous variables with neurons that have overlapping response properties. We developed a *cooperative coding* scheme, which enables similarly tuned neurons to share and distribute computations, crucially using (net) excitatory connections. In general, the signature of cooperative coding is that the network trades feedforward and less specific recurrent synapses for fewer specific recurrent ones.

**Saving synapses.** How can a network save synapses by constructing a given response from different sets of recurrent and feedforward connections? The key observation is that the outputs of few similarly tuned neurons already provide a “large part” of a neuron’s input-output transformation, as well as indirect access to many input neurons. For the simplest considered networks this sharing of computations minimizes the number of required synapses while the total amount of synaptic current remains the same as in a purely feedforward implementation. For networks of neurons that represent higher-dimensional stimuli [65,66], the number of saved synapses is especially large. Our results thus suggest the number of synapses and space constraints as a possible normative reason underlying the cortical like-to-like excitation, which currently lacks such explanation.

**Response time.** The saving of synapses comes at the cost of longer response times. In our most simple, purely excitatory cooperatively coding networks the response is slowed down compared to that of single neurons due to recurrent excitation, which implements a positive feedback loop. This type of amplification has been termed “Hebbian amplification” in [21]. Using rate neuron models, we find, however, that neurons with SFA and neurons in networks in which excitation is largely balanced by delayed inhibition can use the window of opportunity between the arrival of excitation and inhibition to significantly speed up their convergence to the steady state response. This balanced amplification [21] decreases response times by orders of magnitude and improves their scaling with RF size. Implementing cooperative coding in spiking neural networks, we find that these show the same scaling and improvement through balancing inhibition.

Our balanced spiking networks exhibit response times of ∼2–20 ms for RFs of sizes 1–11. This is roughly consistent with experimental measurements of response times, which vary between 5–10 ms measured in V1 single cells from response onset [73] to ≥50 ms measured in populations from stimulus onset [74,75]. However, a direct comparison of our model’s response time with experimental data is challenging, since (1) the onset of feedforward input currents is often unknown in experiments, and (2) our biophysical model parameters have not been matched to any cortical region in particular. Response times are generally thought to increase along the cortical hierarchy [76–79]. This is consistent with an accumulation of response slowdowns and with increased response times for larger RFs, which are both expected in our model.

**Model choices.** Linear rate networks are sufficient for the main part of our study, as we do not model nonlinear phenomena such as feature competition [18]. They allow to capture in an intuitively well-accessible manner the core insight that a desired response can be constructed using few, specific excitatory inputs from similarly tuned neurons (and few feedforward inputs), instead of many feedforward inputs. In addition, linear rate networks can be mathematically well analyzed. This enables a detailed understanding of their response dynamics. In particular, we analytically obtain the network response times and their scaling behavior.

We test the results in spiking neural networks of LIF neurons for a one-dimensional stimulus. Encouragingly, we observe the same qualitative rate dynamics and RFs as in the linear rate networks.

**Networks with SFA.** To speed up the response, we first introduce SFA, a typical feature of excitatory principal neurons [42,50,51]. In the networks with SFA, excitation still dominates, i.e., we have on the one hand Hebbian amplification. On the other hand, we have balanced amplification [21], which speeds up responses. The delayed inhibition thereby originates from private adaptation currents, instead of inhibitory neurons as for balanced amplification in EI-networks.

**Balanced networks.** We model the inhibitory activity in our rate networks as mirroring excitatory activity with an explicit lag. This enables an analysis of the convergence toward equilibrium with techniques from the theory of delay differential equations. Depending on the strength of the balance and the lag of inhibition, this revealed qualitatively different types of dynamics, which are familiar from the harmonic oscillator, namely overdamped, critical, and underdamped dynamics. In our networks with SFA and in balanced amplification networks of others [21], the effective lag of inhibitory feedback originates instead from the fact that inhibitory currents are evoked by a low-pass filtered version of the excitatory activity. In contrast to such models, the inhibitory activity in our balanced networks contains the undamped high-frequency components of the excitatory activity and shifts them by the same delay as the low frequency ones. Because we evaluate our balanced networks at the critical balance, where the time scale of the network dynamics is much larger than the lag, high-frequency components are likely unimportant. Therefore, we do not expect qualitative differences and only small quantitative differences between both implementations in our linear rate networks. Exemplary simulations confirm this expectation, Fig D in S1 Appendix. The reasoning also explains why we find the same qualitative dynamics in our spiking networks, where the inhibitory neurons are fully modeled as LIF neurons with biologically plausible membrane time constants and synaptic delays that govern the delayed feedback to excitatory populations. The basic mechanism of shortening the impulse response and speeding up the reaction to inputs is the same in all these cases.

**RFs in model and experiment.** In the brain the responses of neurons from lower areas are combined to determine the responses of hierarchically higher ones [80,81]. This offers the general opportunity to harness the concept of cooperative coding. RF size often increases along the processing hierarchy, for example by a factor of about 3 to 10 along the ventral visual stream of humans and macaques [82,83]. Ref. [40] suggests a ∼10-fold increase in place field size from dentate gyrus [84] to CA3 [85]. For such changes in RF size, our cooperative coding scheme predicts prominent savings in the number of required synapses compared to purely feedforward networks.

The exponentially decaying RFs that we consider act as tractable models for experimentally encountered localized, overlapping and broadening RFs [2,38,40]. They allow to elegantly illustrate how neurons can use recurrent interactions to cooperatively share feedforward information and shape the network response. However, experimentally measured RFs have different and often more complex shapes [7–9,36]. We are optimistic that these can be approximated by the steady state of a cooperatively coding recurrent network with sparse connectivity, although more synapses will be required. Determining the necessary network parameters might involve minimizing a loss with L0 regularization, which is challenging.

**Related work.** Conceptually, our 1D model is a ring model, operating in the input-driven regime with a single stable ground state [42]. Ring models have been proposed to model orientation selectivity in the visual cortex [23,53] (see also [86]), head direction cells [87] and spatial memory [88]. Similarly, our 2D and 2D mixed selectivity models have a toroidal or, when removing the periodicity, a planar structure. Such networks may be important for spatial navigation [67]. Previous models have broad coupling fields or ranges of coupling probabilities, equivalent to many recurrent synaptic connections that extend over neurons with quite different preferred stimuli [23,42,53,67,87,88]. In contrast, in cooperatively coding networks, we have very sparse synaptic connections between neurons with highly similar tuning.

Our work considers the encoding of continuous variables in a scheme with minimal numbers of required synapses. Ref. [89] investigated a different but related problem: the binary and multinomial classification of random patterns in large networks of neurons with limited and fixed indegrees. The study finds that if an intermediate layer (which is the analogue to our feature layer) is equipped with sparse, excitatory like-to-like recurrent connections, then sparse feedforward connectivity and a sparse readout are sufficient for classification regardless of network size. This connectivity and the resulting saving of synapses imply that the networks realize cooperative coding in our sense. We note that in their Hopfield network intermediate layers could implement cooperative coding by realizing stronger weights with higher (instead of equal) probability.

In a 1D-ring model, ref. [18] had to incorporate strong nearest-neighbor-like excitatory interactions to match experimentally found responses, as they stabilize network responses in the presence of input noise. In our model such connections even determine the RF.

Ref [90] found that local recurrent connectivity in Hebbian assemblies of spiking neurons can reduce the number of feedforward connections between assemblies required for memory replay. The total number of synapses in their model is, however, minimized by a purely feedforward architecture.

Consistent with our results for shallow networks, intermediate-depth ML networks featuring recurrent and feedback connections can match the performance of much deeper feedforward networks while requiring less units and parameters [91]. It would be interesting to investigate whether the recurrent connectivity in such networks is also like-to-like. If so, this would indicate that cooperative coding naturally appears also in ML networks. It may be helpful in particular in convolutional networks, like our models, to save feedforward connections and rely on very sparse recurrent connectivity instead.

**Properties of connectivity.** Our cooperative coding scheme relies on the presence of few strong recurrent excitatory connections between similarly tuned cells; inhibition needs to leave the functional connectivity excitatory. This fits data in visual cortex, which shows that pyramidal neurons with similar RFs connect at higher rates and with stronger synapses [14–16]. Recurrent connections are generally sparse in the cortex [92–95]. Furthermore, net functional connectivity is excitatory between (spatially close) neurons with similar tuning [19] and most correlated responses [18]. Refs. [18,19] also show an inhibitory effect on largely differently tuned neurons. Ref [18] found net inhibition between rather similarly tuned neurons as well. This is assumed to implement feature competition, which we did not include in our model.

A particular benefit of our cooperative coding scheme is that it allows feedforward connections to be sparse. This fits for example experimental observations in V1, where the vast majority of inputs are local recurrent ones, while only a few percent are feedforward inputs [96,97]. Ref. [98] estimated based on experimental studies [99] that a single hypercolumn in primate V1 receives only 10-30 feedforward inputs from the magnocellular layer of dorsal LGN mediating retinal input, with single cells in L4α receiving as little as 0 - 6 inputs. Pyramidal cells in the hippocampal region CA3 may receive input from only about 50 dentate gyrus neurons but from 6000 other CA3 pyramidal cells [100,101]. This is in line with cooperative coding of spatial inputs from dentate gyrus in CA3 and could explain the enlargement of place fields along this pathway [40]. Note, however, that CA3 also receives inputs from entorhinal cortex [102–104].

Experiments that aim to disentangle feedforward from recurrent contributions to orientation selectivity resulted in mixed findings. Ref. [105] showed that excitatory postsynaptic potentials in simple cells in L4 of cat V1 exhibit orientation tuning to drifting gratings, even when recurrent inputs are suppressed by cortical cooling. In line with this, ref. [17] found that thalamic and cortical contributions to the first harmonic (F1) of the response curve to drifting gratings are co-tuned. However, the temporally averaged response (F0) is tuned only in cortical but not in thalamic inputs. A recent study, ref. [106], suggests that the total input current from L4 of mouse primary visual cortex to L2/3 may lack orientation tuning and that orientation selectivity is determined by recurrent inputs from within L2/3.

**Optimality.** We assessed optimality in terms of energy, synapse numbers, and response speed. We find that the simplest, purely excitatory cooperatively coding network minimizes the number of required synapses and has a similar metabolic cost as a feedforward implementation. Adding SFA or balancing inhibition reduces response times but increases metabolic cost. Balancing inhibition also requires additional synapses and neurons. We conclude that the brain might use cooperative coding to save synapses and space compared to a purely feedforward or more wasteful recurrent implementation, but might invest some synapses, neurons, space and energy in balancing inhibition to retain a reasonable response speed.

Previous studies often minimized the number of spikes or, more generally, the neuronal activity needed to represent encoded features. Refs. [30,32,34] follow this approach and suggest that tight EI-balance may be a signature of a highly coordinated and competitive code that, despite the irregular firing, is orders of magnitude more precise than a Poisson rate code. This spike-code depends on an extremely structured, dense connectivity, through which similarly coding neurons quickly inhibit each other to prevent redundant spiking. From this standpoint the findings of excitatory functional connectivity between very similarly tuned neurons [15,16,18,19] seem counter-intuitive.

**Spiking networks.** We verified that the central insights from linear rate networks hold also for biologically more detailed models, by creating cooperatively coding spiking neural networks. In particular, cooperative coding can still save synapses for reasonably sized RFs and introducing balancing inhibition improves the scaling of the response time with the RF size from quadratic to linear. The spiking networks consist of a set of discrete feature populations, with the same connectivity statistics for all neurons within one population. In the stationary state, each feature population encodes as latent variable the stationary activity of a feature neuron of our linear rate networks. The stationary spiking activity thus lies in a low-dimensional manifold of the space spanned by all neurons [107]; the dimensionality is given by the number of feature populations. However, different circuit structures can give rise to the same low-dimensional activity [33,108,109]. We expect that similar dominant latent variables as in our current spiking networks can be reached with spiking neurons arranged quasi-continuously along a ring (or in 2D space) and with tuning-dependent sparse like-to-like connectivity that realizes cooperative coding.

**Sensitivity to recurrent weight strength.** To produce larger RFs, the cooperative network requires larger recurrent weights, which converge toward a critical value. Thus, for larger RFs, the cooperative network is increasingly sensitive toward variation in the recurrent weight and an accurate numerical integration of the spiking networks requires increasingly fine discretization time steps (cf. Fig E in S1 Appendix). Stability can be increased in a hybrid coding model, which uses a small fraction of the inputs from the feedforward model, and complements them with cooperatively coding recurrent inputs. We expect that this may again save synapses compared to the feedforward model.

**Experimental predictions.** In our cooperatively coding models, feedforward input is sparse [98,99,110] and, compared to recurrent input, weak [17]. It directly contributes to only a small part of a neuron’s receptive field or response. In our models, only the center neuron/population receives direct feedforward input. When taken literally, our model predicts strong excitatory connections only between very similarly tuned cells, consistent with some recent experimental findings [18]. A hybrid coding model would yield co-tuning of feedforward and recurrent inputs [17,81,105]. In either case, feedforward input is amplified through recurrent connections, which redistribute it and thereby establish the full response. Our models thus predict that removal of recurrence should lead to responses and receptive fields that are much smaller in amplitude, and deficient in the sense that they lack responses to many inputs within the full RF.

**Conclusion.** To conclude, net excitatory connectivity between similarly tuned neurons is compatible with a novel cooperative coding scheme that generates network responses with a minimal number of synapses. This suggests space constraints as an important factor in shaping neural networks, providing a possible normative explanation for excitatory like-to-like connectivity. The window of opportunity between excitation and balancing, delayed adaptation or inhibition may be harnessed to rapidly propagate activity changes through the network, speeding up equilibration times by orders of magnitude.

## Methods

### Rate models

All simulations have periodic boundary conditions. Fixed network parameters are the number of neurons *N* for 1D and ${\text{N}}^{2}$ for 2D networks, the neuronal time-constant τ and, in networks with inhibition, the EI-lag $\tau_{lag}$. We set *N* = 200, τ=1 and $\tau_{lag}=0.1$. In the networks with SFA we use a fixed value of $\tau_{SFA}=\tau =1$ and, for each RF size, obtain the value of $a_{SFA}$ that minimizes the temporal mean of the normalized L1-loss, $\left(1/T)\int_{0}^{T}dt\;|x(t\right)$ − x^*^(*t*)|_1_/|x^*^|_1_, through a linear grid search. Here T=500τ is the length of a trial as described in Fig Aa in S1 Appendix and ${\text{x}}^{*}(\text{t})$ the target corresponding to the present input. Fig Ae,c show the scans over $a_{SFA}$ and the individual loss curves for the optimal $a_{SFA}$ values. In all networks with inhibition, we set $w_{sum}^{rec,bal}$ to its critical value given by Eq S43 in S1 Appendix.

We simulate our networks with SFA using the Euler method and all other networks using the midpoint method with stepsize dt=0.01. To simulate the networks with delayed inhibition, we also need midpoint values of the delayed activity. We obtain them by copying the midpoint values of the non-delayed activity $\tau_{lag}$ ($\tau_{lag}/\;dt$ simulation steps) before.

For the data in Figs 4 and 6, we obtain RFs with different sizes by setting $w_{sum}^{rec}$ or $w_{sum}^{rec,net}$ to appropriate values: In the case of 1D networks with and without SFA and in the case of 2D linear MS networks, we have analytical expressions for the RF sizes as a function of $w_{sum}^{rec}$ or $w_{sum}^{rec,net}$. We thus chose $w_{sum}^{rec}$ or $w_{sum}^{rec,net}$ such that the RF sizes are sampled linearly from $n_{RF}^{1D}=6$ to $n_{RF}^{1D}=50$ in steps of two. For the 2D network, we simulate networks with 20 different values of $w_{sum}^{rec}$ or $w_{sum}^{rec,net}$ and measure the RF sizes that the networks generate after convergence. We obtain $w_{sum}^{rec}$ or $w_{sum}^{rec,net}$ as $w_{sum}^{rec}(\text{or }w_{sum}^{rec,net})=1$ − $\tau /\tau_{resp}$ by varying $\tau_{resp}$ from 10 to 1,000 with equal spacing on a logarithmic scale.

To numerically determine a network’s response time, we first simulate the network for a long time, clearly longer than the convergence time, and define the resulting state as the final, target state ${\text{x}}^{*}$. The loss is the L1 norm of the difference between ${\text{x}}^{*}$ and the current state. We then simulate the network for a second time. We obtain $\tau_{resp}$ or $\tau_{resp}^{bal}$ as the earliest time at which the loss drops and stays below $e^{-1}$ times the initial loss.

### Spiking models

#### Excitatory network.

We model *N*_*F*_ feature populations arranged on a one-dimensional ring. Every feature population contains *N*_*E*_ excitatory neurons, which are modeled as leaky integrate-and-fire (LIF) neurons. Feature populations are connected randomly and sparsely to their neighbors, as well as recurrently within themselves.

The membrane potential of neuron *k* in feature population *i* is described by the stochastic differential equation (SDE)

$$\tau_{m}{\dot{v}}_{ik}=-v_{ik}+V_{rest}+I_{ext}^{i}+w_{EE}\tau_{m}\sum_{\Gamma_{ik}^{E}}\sum_{\hat{t}}\delta \left(t-\hat{t}-\Delta_{EE}\right)+\sqrt{2\tau_{m}}\sigma \xi_{ik}(t)\;.$$ (47)

We set $V_{rest}=0$ mV and leave it out in the following to simplify the notation. When the membrane potential reaches the threshold $V_{thr}$, a spike is generated and the membrane potential is reset to $V_{reset}$. There is no absolute refractory period. All neurons of a feature population *i* receive the same feedforward input $I_{ext}^{i}$: For the stimulated population (denoted as *i* = 0 in the following) this feedforward input is stronger ($I_{ext}^{0}=I_{ext}^{on}$), while all other populations receive a lower background input ($I_{ext}^{i}=I_{ext}^{off},\;i\neq 0$). Each neuron receives independent Gaussian white noise ξ with $\langle \xi_{ik}(t)\rangle =0$, and $\langle \xi_{ik}(t)\xi_{jl}(t^{\prime })\rangle =\delta_{ij}\delta_{kl}\delta (t-t^{\prime })$; σ specifies its strength. Further, a neuron receives excitatory input from a pool $\Gamma_{ik}^{E}=\Gamma_{ik}^{E,in}$ ∪ $\Gamma_{ik}^{E,out}$ of neurons from its own population *i* ($\Gamma_{ik}^{E,in}$) as well as from the two neighboring populations i±1 ($\Gamma_{ik}^{E,out}$). The pool of presynaptic neurons is constructed by drawing from each admissible population $K_{EE}=p_{EE}N_{E}$ presynaptic neurons (excluding autapses) — i.e., the indegree is fixed. The inner sum in Eq 47 is taken across all spike times $\hat{t}$ of all neurons in these presynaptic pools. Each presynaptic spike induces a jump of the postsynaptic membrane potential of size $w_{EE}$ after a delay $\Delta_{EE}$. Synaptic delays are drawn randomly from a uniform distribution between 0 and 2 ms. Table 1 summarizes all parameter values. The tuning of the recurrent synaptic weight strength $w_{EE}$ and the feedforward input $I_{ext}^{on},I_{ext}^{off}$ is described in Eq S74 – S77 in S1 Appendix.

**Table 1:** Parameters of spiking network models. Top: excitatory-only network (Fig 8). Bottom: additional parameters for balanced network (Fig 10). Equation numbers refer to S1 Appendix.

Parameter	Value	Definition
*N*_*F*_	41	Number of feature populations
*N*_*E*_	4000	Excitatory neurons per feature population
$p_{EE}$	0.1	Connection probability from E to E
$w_{EE}$	Eq S75	Synaptic weight (E to E)
$V_{thr}$	10 mV	Spike threshold
$V_{rest}$	0 mV	Resting voltage
$V_{reset}$	0 mV	Reset voltage
$\tau_{m}$	20 ms	Membrane time constant
$\tau_{ref}$	0 ms	Refractory period
$\Delta_{EE}$	∼𝒰(0,2) ms	Synaptic delay from E to E
**dt**	**0.01 ms**	**Simulation time step**
***N*_*I*_**	**1000**	**Inhibitory neurons per feature population**
$p_{EI}$	0.1	Connection probability from I to E
$p_{IE}$	0.1	Connection probability from E to I
${\bar{w}}_{EE}$	Eq S79	(increased) Synaptic weight (E to E)
$w_{IE}$	Eq S78	Synaptic weight (E to I)
$w_{EI}$	Eq S81	Synaptic weight (I to E)
$\Delta_{IE}$	∼𝒰(0,2) ms	Synaptic delay from E to I
$\Delta_{EI}$	∼𝒰(0,2) ms	Synaptic delay from I to E

#### Balanced network.

We add *N*_*F*_ inhibitory feature populations, each containing *N*_*I*_ inhibitory neurons. The membrane potential of a neuron *k* in excitatory feature population *i* is given by the SDE

$$\tau_{m}{\dot{v}}_{ik}^{E}=-v_{ik}^{E}+I_{ext}^{i}+{\bar{w}}_{EE}\tau_{m}\sum_{\Gamma_{ik}^{E,in}\cup \Gamma_{ik}^{E,out}}\sum_{\hat{t}}\delta \left(t-\hat{t}-\Delta_{EE}\right)\;\;+w_{EI}\tau_{m}\sum_{\Gamma_{ik}^{I,in}\cup \Gamma_{ik}^{I,out}}\sum_{\hat{t}}\delta \left(t-\hat{t}-\Delta_{EI}\right)+\sqrt{2\tau_{m}}\sigma \xi_{ik}^{E}(t)\;.$$ (48)

The membrane potential of a neuron *k* in inhibitory feature population *i* similarly obeys

$$\tau_{m}{\dot{v}}_{ik}^{I}=-v_{ik}^{I}+I_{ext}^{off}+w_{IE}\tau_{m}\sum_{\Gamma_{ik}^{E,in}}\sum_{\hat{t}}\delta \left(t-\hat{t}-\Delta_{IE}\right)+\sqrt{2\tau_{m}}\sigma \xi_{ik}^{I}(t)\;.$$ (49)

Excitatory and inhibitory populations are connected as described for the balanced rate network and illustrated in Fig 10A: An inhibitory neuron *k* in population *i* receives excitatory synaptic input from the excitatory partner population ($\Gamma_{ik}^{E,in}$). An excitatory neuron *k* in population *i* receives synaptic inputs from the inhibitory partner population ($\Gamma_{ik}^{I,in}$), as well as its two neighbors ($\Gamma_{ik}^{I,out}$). Excitatory-to-excitatory connections are as in the purely excitatory network described above. For all pathways, synapses are again drawn randomly with a connection probability of 10%, while imposing a fixed indegree. Inhibitory neurons have the same biophysical parameters as excitatory cells. All synaptic delays are drawn randomly from a uniform distribution between 0 and 2 ms. Table 1 summarizes all parameter values. The feedforward input to inhibitory neurons is untuned. The tuning of the weights between excitatory and inhibitory populations is described in Eq S78 and S81 in S1 Appendix.

#### Numerical simulations.

All spiking network simulations are performed using the spiking network simulator Brian2 [111]. For RF sizes of $n_{RF}\leq 13$ we simulate *N*_*F*_ = 41 feature populations. For larger fields ($n_{RF}\geq 15$) we use *N*_*F*_ = 61 to reduce boundary effects. The feedforward stimulation is chosen to target the central population (i.e., population 21 for *N*_*F*_ = 41 or population 31 for *N*_*F*_ = 61, respectively). First, the network is simulated for 500 ms with only background-level feedforward input. Then the feedforward input to the stimulated population is increased instantaneously and the network is simulated for three seconds to ensure that a stable state has been reached.

We estimate the stationary firing rates by averaging over the last second of the simulation (second 2–3 after stimulus onset). Response times are estimated as described for the rate model, based on the L1 loss of the instantaneous population rates after *t* = 500ms, using an exponential fit. For response time estimation, we use unfiltered population rates, computed as the average number of spikes per simulation time bin. For plotting, population rates are displayed in larger bins of 1 ms.

We numerically optimized the excitatory synaptic weights in Fig 8C by repeatedly simulating the network with fine variations in the weight parameter, until a rate profile within the range of $\left[n_{RF}^{target}-0.5,\;n_{RF}^{target}\right]$ was found. Note that we did not enforce a strict match of the peak rate $x_{max}$ in this optimization process, which would require a covariation of the stimulation strength $I_{ext}^{on}$.

The “optimal” scaling factors in Fig 10C were found by simulating balanced excitatory-inhibitory networks for each field size with scaling factors varied in the range of s∈[1.1,1.9] in steps of 0.1 (see horizontal dashed lines in Fig 10C, bottom). For each scaling factor the (analytically predicted) balancing inhibitory synaptic strength (Eq S81 in S1 Appendix) had to be optimized numerically in order to assure a close match of the EI-rate profile to the target.

Numerical integration was performed in Brian2 using a simple Euler-Maruyama scheme with a discrete time step of 0.01 ms. As the RF size increases, the recurrent weights of the cooperative network approach the point where the network becomes unstable. Close to this instability point, an accurate “clock-driven” numerical integration of the network dynamics requires increasingly small time steps. With spike times restricted to the “grid” set by {0,dt,2dt,3dt,…}, spikes can be missed or recorded late [112]. This affects not only the slope of the rate response at the onset of a step stimulus, but also the steady state into which the rates settle. This effect can already be observed in a single population with recurrent coupling approaching the instability point, Fig F in S1 Appendix. In our cooperative network with multiple coupled populations, the time step sensitivity increases for larger fields. For the simulations shown here we used a timestep of 0.01 ms. At this resolution we find satisfactory convergence of the dynamics of networks tuned to RF sizes $n_{RF}\leq$ 11. Larger RF sizes would require even smaller time steps, see Fig E in S1 Appendix.

## Supporting information

S1 Appendix Details, derivations, proofs and supporting simulations. In Sec A we derive the stationary state and the evolution of eigenmodes for the 1D cooperatively coding networks without SFA and balancing inhibition. Section B derives the evolution of their L1 loss. In Sec C we proof that their architecture indeed minimizes the number of synapses. Section D argues that we can compare the metabolic cost of generating the stationary state in the different implementations by the L1 norms of their synaptic currents. Sec E and Fig A in S1 Appendix study the dynamics of a 1D cooperatively coding network with SFA. In Sec F we define and calculate the effective strength of the balanced interaction. Section G derives the evolution of the L1 loss for cooperatively coding networks with delayed, balancing inhibition, and expressions for the critical balance strength and network response time. Fig B in S1 Appendix characterizes the loss evolution for different balance strengths. In Sec H and Fig C in S1 Appendix we explain the difference between the loss evolution after constant initialization and in the slowliest-decaying eigenmode. Section I and Fig D in S1 Appendix demonstrate that dynamics are similar whether inhibition is modeled with an explicit lag or leakily integrates excitation. In Sec J we derive the stationary response of networks that cooperatively encode 2D stimuli. In Sec K we derive an analytical tuning of the excitatory and balanced spiking networks as a function of RF size and peak rate. We comment on the additional numerical optimization of recurrent synaptic weights. In Sec L we illustrate the need for small discretization time steps for the numerical integration of spiking networks with large RFs (Fig E in S1 Appendix), and analyze the sensitivity of RF formation with respect to the recurrent weight strength (Fig G in S1 Appendix), and finite size fluctuations (Fig H in S1 Appendix). In Sec M we present an alternative, homogeneous network architecture, for which we observe a similar response speedup when balancing inhibition is added (Fig I in S1 Appendix)
(PDF)
