# Supplementary material for: Cooperative coding of continuous variables in networks with sparsity constraint
Source: PLoS Comput Biol. 2025 Jul 3;21(7):e1012156. doi: 10.1371/journal.pcbi.1012156 (PMC12407556; doi:10.1371/journal.pcbi.1012156)
Supplement: S1 Appendix — In Sec A we derive the stationary state and the evolution of eigenmodes for the 1D cooperatively coding networks without SFA and balancing inhibition. Section B derives the evolution of their L1 loss. In Sec C we proof that their architecture indeed minimizes the number of synapses. Section D argues that we can compare the metabolic cost of generating the stationary state in the different implementations by the L1 norms of their synaptic currents. Sec E and Fig A in S1 Appendix study the dynamics of a 1D cooperatively coding network with SFA. In Sec F we define and calculate the effective strength of the balanced interaction. Section G derives the evolution of the L1 loss for cooperatively coding networks with delayed, balancing inhibition, and expressions for the critical balance strength and network response time. Fig B in S1 Appendix characterizes the loss evolution for different balance strengths. In Sec H and Fig C in S1 Appendix we explain the difference between the loss evolution after constant initialization and in the slowliest-decaying eigenmode. Section I and Fig D in S1 Appendix demonstrate that dynamics are similar whether inhibition is modeled with an explicit lag or leakily integrates excitation. In Sec J we derive the stationary response of networks that cooperatively encode 2D stimuli. In Sec K we derive an analytical tuning of the excitatory and balanced spiking networks as a function of RF size and peak rate. We comment on the additional numerical optimization of recurrent synaptic weights. In Sec L we illustrate the need for small discretization time steps for the numerical integration of spiking networks with large RFs (Fig E in S1 Appendix), and analyze the sensitivity of RF formation with respect to the recurrent weight strength (Fig G in S1 Appendix), and finite size fluctuations (Fig H in S1 Appendix). In Sec M we present an alternative, homogeneous network architecture, for which we observe a similar response speedup when balancing inhibiti [file pcbi.1012156.s001.pdf]

Cooperative coding of continuous variables in networks with sparsity constraint – Supplement S1 Appendix –

Paul Züge[•, 1], Natalie Schieferstein[◦, 1], Raoul-Martin Memmesheimer[*,1]

[1]Institute for Genetics, University of Bonn, Germany

• pzuege@uni-bonn.de, ◦ nschiefe@uni-bonn.de, ∗ rm.memmesheimer@uni-bonn.de

Contents

A Dynamics of excitatory networks **1**

B Loss evolution of excitatory networks **3**

C Cooperative coding minimizes the number of required synapses in 1D excitatory networks **4**

D Metabolic cost **4**

E Spike frequency adaptation **5**

F Effective balanced interaction strength **5**

G Loss evolution of balanced networks **7**

H Initial response of balanced networks **12**

I Leaky integrator inhibition **14**

J Stationary solution for 2D stimulus **15**

K Tuning spiking networks for cooperative coding **16**

L Stability of cooperative coding in spiking networks **19**

M Cooperative coding in spiking networks with homogeneous E/I populations **24**

A Dynamics of excitatory networks

The dynamics of our networks without SFA and explicit inhibition read for constant input $r_i(t) = r_i$

$$\tau \dot{x}_i(t) = -x_i(t) + \sum_j W^{\mathrm{rec}}_{ij} x_j(t) + \sum_j W^{\mathrm{ff}}_{ij} r_j, \tag{S1}$$

see Eq (4). In the following, we discuss stationary states and time-dependent behaviors of these dynamics.

We first verify that the recurrent network Eq (5), where $W_{ij}^{\mathrm{rec}} = (\delta_{i+1,j} + \delta_{i-1,j})/(\gamma + \gamma^{-1})$ and $W_{ij}^{\mathrm{ff}} = \delta_{ij}\left(1 - 2\gamma/(\gamma + \gamma^{-1})\right)$, has the desired stationary state $x_i^{\mathrm{resp}} = \sum_j \gamma^{|i-j|} r_j$. For this we insert Eq (1) into Eq (5),

$$\tau \dot{x}_i = -\sum_j \gamma^{|i-j|} r_j + \frac{1}{\gamma + \gamma^{-1}} \left(\sum_j \gamma^{|i+1-j|} r_j + \sum_j \gamma^{|i-1-j|} r_j \right) + \left(1 - \frac{2\gamma}{\gamma + \gamma^{-1}}\right) r_i \tag{S2}$$

$$= -\sum_j \gamma^{|i-j|} r_j + \frac{1}{\gamma + \gamma^{-1}} \left(\sum_{j<i+1} \gamma^{i+1-j} r_j + \sum_{j \geq i+1} \gamma^{j-i-1} r_j + \sum_{j \leq i-1} \gamma^{i-1-j} r_j + \sum_{j>i-1} \gamma^{j-i+1} r_j \right) + \left(1 - \frac{2\gamma}{\gamma + \gamma^{-1}}\right) r_i \tag{S3}$$

$$= -\sum_j \gamma^{|i-j|} r_j + \frac{1}{\gamma + \gamma^{-1}} \left(\gamma \sum_{j \leq i} \gamma^{i-j} r_j + \gamma^{-1} \sum_{j>i} \gamma^{j-i} r_j + \gamma^{-1} \sum_{j<i} \gamma^{i-j} r_j + \gamma \sum_{j \geq i} \gamma^{j-i} r_j \right) + \left(1 - \frac{2\gamma}{\gamma + \gamma^{-1}}\right) r_i \tag{S4}$$

$$= -\sum_j \gamma^{|i-j|} r_j + \frac{1}{\gamma + \gamma^{-1}} \left(\gamma \sum_j \gamma^{|i-j|} r_j + \gamma r_i + \gamma^{-1} \sum_j \gamma^{|i-j|} r_j - \gamma^{-1} r_i \right) + \frac{\gamma + \gamma^{-1} - 2\gamma}{\gamma + \gamma^{-1}} r_i \tag{S5}$$

$$= \frac{\gamma - \gamma^{-1}}{\gamma + \gamma^{-1}} r_i + \frac{\gamma^{-1} - \gamma}{\gamma + \gamma^{-1}} r_i = 0 \tag{S6}$$

The computation shows that, in the steady state, the recurrent input from neurons $i+1$ and $i-1$ add up to nearly generate the desired response of neuron i: the first and third summand in the bracket in Eq (S5) give the desired response. The input that is missing, $\frac{\gamma - \gamma^{-1}}{\gamma + \gamma^{-1}} r_i < 0$, is contributed by the feedforward input (last summand in each line, which cancels the missing input in Eq (S6)).

We now turn to non-stationary solutions. The recurrent weight matrices in our recurrent networks Eq (5) are real symmetric matrices and therefore diagonalizable with real eigenvalues and orthogonal eigenvectors. We denote by $\hat{x}^\mu$, $\mu = 0, \cdots, N-1$, the μth normalized eigenvector of W^{rec} with eigenvalue α^μ, and by $x^\mu(t) = \hat{x}^{\mu T} x(t)$ the projection of the network activity on this eigenvector (a scalar). Any activity $x(t)$ can be expressed as a linear combination of the orthonormal eigenvectors $\hat{x}^\mu$ with coefficients $x^\mu(t)$. $\hat{x}^\mu$ is also an eigenvector of $W^{\mathrm{rec}} - \mathbb{1}$, with eigenvalue $\alpha^\mu - 1$. Multiplying Eq (S1) with $\hat{x}^{\mu T}$ from the left shows that the evolution of network activity can be separated into the evolution of N individual components,

$$\tau \dot{x}^\mu(t) = -x^\mu(t) + \alpha^\mu x^\mu(t) + \sum_{i,j} \hat{x}_i^\mu W_{ij}^{\mathrm{ff}} r_j, \tag{S7}$$

$$\dot{x}^\mu(t) = -\lambda^\mu x^\mu(t) + \frac{1}{\tau} \sum_{i,j} \hat{x}_i^\mu W_{ij}^{\mathrm{ff}} r_j, \tag{S8}$$

see, e.g., [1]. Here we defined

$$\lambda^\mu = \frac{1 - \alpha^\mu}{\tau} \tag{S9}$$

as the decay rate of network activity in the μth eigenmode. The response time of the network equals $1/\lambda^\mu$, if it is initialized in the μth eigenmode. When initialized by $x(0)$, the state decays to a stationary value in the different components,

$$x^\mu(t) = \mathrm{e}^{-\lambda^\mu t} x^\mu(0) + (1 - \mathrm{e}^{-\lambda^\mu t}) x^{*\mu}. \tag{S10}$$

Here $x^\mu(0) = \hat{x}^{\mu T} x(0)$ are the initial and $x^{*\mu} = \hat{x}^{\mu T} x^* = \frac{1}{\lambda^\mu \tau} \sum_{i,j} \hat{x}_i^\mu W_{ij}^{\mathrm{ff}} r_j$ the final stationary values of the μth component in the eigenbasis. The vector of the stationary dynamics x^* has the entries $x_i^* = \sum_{j=1}^N \mathrm{RF}_{ij} r_j$, if the network generates the desired stationary dynamics. In the absence of recurrent input we have $W^{\mathrm{rec}} = 0$ and thus $\alpha^\mu = 0$, such that the activity decays at a rate of $\lambda = 1/\tau$ to its target. Positive eigenvalues $\alpha^\mu > 0$ mean a slower decay. At $\alpha^\mu = 1$ there is no decay at all, and for $\alpha^\mu > 1$ activity diverges. Stable activity thus requires the largest eigenvalue of the recurrent weight matrix to be smaller than one, such that together with the individual intrinsic decay of each neuron the dynamics are a contraction. Because the system is linear, the different eigenmodes decay independently from each other at different rates, following Eq (S10). Thus with time the faster-decaying modes connected to smaller eigenvalues become exponentially suppressed relative to the dominant, slowest-decaying mode.

The recurrent weight matrices in our recurrent networks Eq (5) furthermore have the property that they are circulant matrices, i.e., each row is equal to the row before rotated one element to the right. The eigenvalues of such matrices are given by the explicit formula [2]

$$\alpha^\mu = \sum_{j=1}^N W_{1j}^{\mathrm{rec}} \cdot \mathrm{e}^{\frac{2\pi I}{N}(j-1)\mu}, \tag{S11}$$

where I is the imaginary unit. For purely excitatory networks, we have $W_{1j}^{\mathrm{rec}} \geq 0$, such that α^μ is maximal if $\mu = 0$, as otherwise the real part of each addend in Eq (S11) is smaller or equal. Therefore we obtain the slowest convergence for

$$\alpha^{\max} = \alpha^0 = w_{\mathrm{sum}}^{\mathrm{rec}} = \sum_{j=1}^N W_{ij}^{\mathrm{rec}}. \tag{S12}$$

$w_{\mathrm{sum}}^{\mathrm{rec}}$ is the row sum of the recurrent coupling matrix, which is independent of i as the matrix is circulant. The corresponding eigenvector of W^{rec} is

$$\hat{x}^0 = \frac{1}{\sqrt{N}} (1, 1, 1, ..., 1)^T. \tag{S13}$$

The slowest exponential decay dominates the behavior for longer times. Inserting $\alpha^{\max}$ into Eq (S9) thus yields τ_{resp}, the generic time scale of convergence of the network dynamics Eq (5) to the target state $x_i = \sum_j \mathrm{RF}_{ij} r_j$,

$$\tau_{\mathrm{resp}} = \frac{\tau}{1 - \alpha^0} = \frac{\tau}{1 - w_{\mathrm{sum}}^{\mathrm{rec}}}. \tag{S14}$$

B Loss evolution of excitatory networks

In this section, we compute the time evolution of the network loss for our networks without SFA and explicit inhibition. We assume that the networks receive constant input and have initial state $x_i(0) = 0$. All convergence time scales that are present in the network (cf. Eqs (S9) and (S11)) could in principle contribute to this time evolution. We will however see, that the decay time of the loss is equal to that of the slowest-decaying mode.

We define the loss as the 1-norm of the deviation of the network activity from the target activity,

$$L(t) = \frac{1}{N} \sum_{i=1}^N |x_i(t) - x_i^*|. \tag{S15}$$

Under the assumption that network activity does not "overshoot", i.e., that it is always lower than or equal to the target activity, $x_i(t) \leq x_i^* \; \forall t$, we can replace the loss function by a linear loss function

$$L^{\mathrm{lin}}(t) = \frac{1}{N} \sum_{i=1}^{N} x_i^* - x_i(t). \tag{S16}$$

We can express the linearized loss as the (scaled) projection of the deviation of the activity from its target, $x^* - x(t)$, onto the eigenvector $\hat{x}^0$ Eq (S13),

$$L^{\mathrm{lin}}(t) = \frac{1}{\sqrt{N}} \hat{x}^{0T} \left(x^* - x(t) \right) = \frac{1}{\sqrt{N}} \left(x^{*0} - x^0(t) \right). \tag{S17}$$

The linear loss thus has a single exponential decay. This is a consequence of the fact that $(1, 1, 1, ...1)^T$ is an eigenvector of the dynamics, which, in turn, holds because the row sum of a circulant matrix is the same for each row. The linear loss therefore decays with the time constant τ_{resp} obtained in Eq (S14) to its stationary value, 0.

C Cooperative coding minimizes the number of required synapses in 1D excitatory networks

The cooperatively coding recurrent networks Eq (5) and, with SFA, Eq (19) generate the required stationary dynamics Eq (1) with three synapses per neuron. Here we show that this is the minimal number of synapses. Specifically, we show that it is impossible to construct the RF of one neuron as the sum of only one or two other RFs and/or feedforward inputs.

Any network of the form Eq (4) that correctly implements the desired target response has RFs that satisfy Eq (10), which we reproduce in reordered manner for the convenience of the reader,

$$\mathrm{RF}_{ij} = W_{ij}^{\mathrm{ff}} + \sum_k W_{ik}^{\mathrm{rec}} \mathrm{RF}_{kj}, \tag{S18}$$

i.e., $\mathrm{RF}_{i\cdot}$ is a sum of localized peaks from feedforward inputs and extended two-sided exponentials from recurrent inputs (see Fig 2C for an example). First, there needs to be at least one (nonzero) feedforward synapse, as otherwise the network could not respond to input neuron activity. Clearly two feedforward synapses cannot solve the problem for RFs with $n_{\mathrm{RF}} > 2$. But the sum of a localized peak from a feedforward input and the extended response from a recurrent input (with nonzero coefficients) cannot match the target shape, because the difference between the target RF and another RF is either zero or has extended support. Thus every neuron needs at least three synapses to implement the target network response.

D Metabolic cost

In the following we compare the metabolic cost of the feedforward and the simplest, purely excitatory cooperative coding networks in the stationary state. We consider the three main contributions of the metabolic cost [3,4]: the cost of keeping up the resting network, of generating the required activity and of the synaptic transmissions. Keeping up the resting network (housekeeping and maintaining the resting potentials) requires the same energy expenditure in both implementations, as the number of neurons is the same. Also the energy required to generate the stationary output activity is the same, as corresponding neurons generate the same activity in both implementations.

We thus focus on the cost of synaptic transmission. This is dominated by the metabolic cost caused by the postsynaptic currents [4]. We assume that this cost is characterized by the sum of the absolute current strengths at individual synapses, i.e., by the L1 norm of the synaptic currents [5]. Finally, a small part of the overall cost (less than 10% [4]) arises due to presynaptic calcium influx and usage of neurotransmitter during synaptic transmission. A comparison of these contributions between the network implementations is

more difficult. The surface of the active zone [6] increases linearly with the synaptic strength [7]. Assuming that the area where calcium influx happens increases linearly with the active zone size, the amount of influx during a single synaptic transmission depends linearly on the synaptic strength, consistent with [8]. The same should then hold for the related cost. Concerning the amount of neurotransmitter used, experiments observe that the number of directly releasable vesicles is linearly related to the synaptic strength, while the impact of a single vesicle and its release probability are independent of it [7, 9] (this may differ for different connected neuron types [10]). The finding suggests a linear dependence between the synaptic strength and the used neurotransmitter and thus the related cost at a single transmission. The total cost due to presynaptic calcium influx and neurotransmitter usage is therefore proportional to the absolute synaptic strength times the number of transmissions (the presynaptic activity). This, in turn, is proportional to the absolute value of the induced postsynaptic current.

We conclude that we can compare the metabolic cost of generating the stationary state between the different network implementations by comparing the L1 norms of the synaptic currents. We argue in the main text that these are the same in both implementations.

E Spike frequency adaptation

Here we study the dynamics of a 1D network with SFA, which are given by Eq (19). Fig Aa shows the dynamic response of neuron j_0, whose preferred input is presented as isolated unit input. The initial dynamics are faster than for a network without SFA. This is because the adaptation current effectively reduces the membrane time constant and because it does not yet fully compensate for the stronger recurrent and feedforward weights, as it is slow (see Eqs (16) and (18)).

To quantify the change in response speed, we compute the integrated loss, as fast convergence implies that the integrated loss is small. We normalize the loss, $\text{Loss}(t) = |x(t) - x^*|_1/|x^*|_1$ to render it comparable for different RF widths. We define the integrated loss as the temporal mean of the normalized loss. Fig Ad shows the dependence of the integrated loss on the SFA time constant. The left-most data point at $\tau_{\text{SFA}} = 0$ corresponds to the zero-lag limit where $u_i(t) = x_i(t)$, which effectively modifies the leak current to $-(1 + a_{\text{SFA}})x_i(t)$ (Eq (18)), resulting in a reduced neuronal time constant $\tau \to \tau/(1 + a_{\text{SFA}})$. Consequently, in this limit and for $a_{\text{SFA}} = 1$, the response time of the SFA network is half as large as that of a network without SFA, which is $\tau_{\text{resp}} = \tau/(1 - w_{\text{sum}}^{\text{rec}}) \approx 201$, Eq (13). Also the integrated loss is smaller; without SFA it would be ≈ 0.413. The negative slope at $\tau_{\text{SFA}} = 0$ and the clear minimum at $\tau_{\text{SFA}} \approx 0.87$ show that the network can increase its response speed beyond this effective reduction of τ by utilizing the larger recurrent and feedforward weights, Eq (17). For oscillating dynamics, τ_{resp} can jump discontinuously. This happens if the time point at which all later losses are below e^{-1} jumps from one oscillation peak to the next oscillation. Therefore we use the integrated loss here as the minimization target.

Fig Af shows the integrated loss as a function of a_{SFA} and $w_{\text{sum}}^{\text{rec,bal}}$ in networks that combine SFA and balancing inhibition. The integrated loss is minimized in networks with critical EI-balance and without SFA. Larger adaptation can partly compensate weaker balance; the data suggest that the optimal values of the adaptation and balance parameters satisfy the relation $a_{\text{SFA}}\tau_{\text{SFA}} + w_{\text{sum}}^{\text{rec,bal}}/w_{\text{sum},c}^{\text{rec,bal}} \approx 1$.

F Effective balanced interaction strength

The balanced interaction term in the dynamics Eq (26) reads

$$\tau \dot{x}_i = \ldots + \sum_{j=1}^{N} W_{ij}^{\text{rec,bal}} \Delta x_j(t) + \ldots . \tag{S19}$$

In the following we compute the effective strength of the interaction mediated by this term between two neurons j and i. For this we assume that the presynaptic activity x_j of neuron j changes only for a limited amount of time, i.e., its derivative has limited support. Concretely we assume that $\dot{x}_j(t) = 0$ for $|t| > T - \tau_{\text{lag}}$ for some finite time T, denoting $\delta x_j = x_j(T) - x_j(-T)$ as the total change in presynaptic activity. We

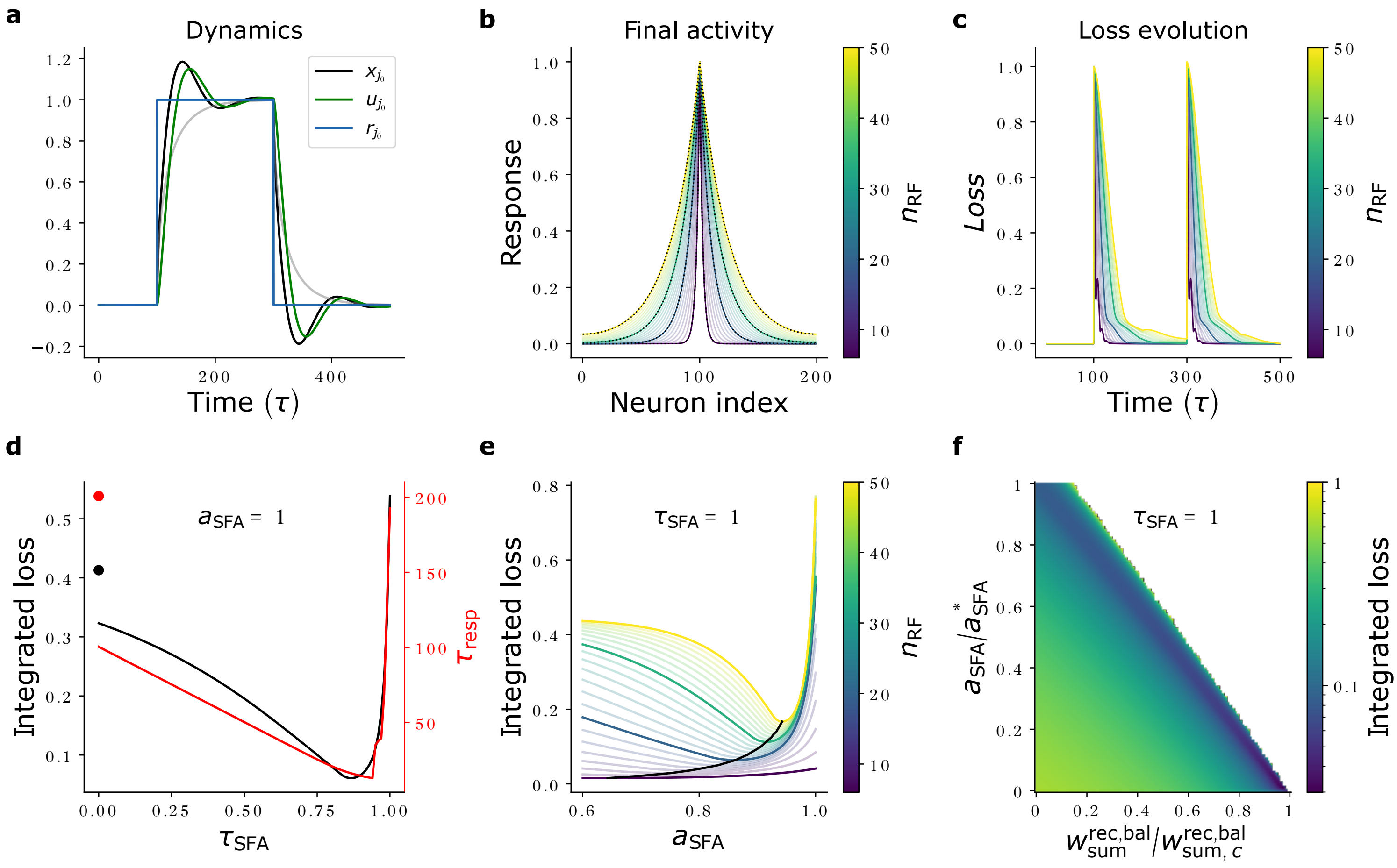

Figure A: **Network and loss dynamics in a 1D network with SFA. (a)** Dynamics of activity ($x_{j_0}(t)$, black), adaptation variable ($u_{j_0}(t)$, green) and input ($r_{j_0}(t)$, blue) for the $j_0 = 100$th neuron, which receives its preferred input as isolated unit input that is switched on at $t = 100\tau$ and off at $t = 300\tau$ ($r_j(t) = \delta_{jj_0}$ between these times). The network implements an RF with $d = 4.5$ ($n_{\mathrm{RF}} = 10$) and has, for better illustration, a rather slow $\tau_{\mathrm{SFA}} = 10\tau$ and slightly stronger-than-optimal $a_{\mathrm{SFA}} = 0.09$, resulting in a visible lag between $x(t)$ and $u(t)$ and oscillatory dynamics. During the initial rising phase ($t \gtrsim 100\tau$), activity rises faster than for a network without SFA (gray curve). **(b)** Stationary activity of SFA networks with different RF widths. The networks receive an isolated input (parameters are $\tau_{\mathrm{SFA}} = \tau$ and optimal $a_{\mathrm{SFA}} = a^*_{\mathrm{SFA}}$, see (e)). The stationary activity matches its target for different n_{RF} (color-coded). To show this, target (black dashed) and final activity (color-coded with intense colors) of four networks with $n_{\mathrm{RF}} = 6, 20, 34, 50$ are highlighted. **(c)** Evolution of the L1-loss of the deviation of network activity from its target, normalized by the L1-norm of the target response for present input, under the stimulus protocol as described in (a) for the same networks as in (b), with $a_{\mathrm{SFA}} = a^*_{\mathrm{SFA}}$ determined as described in (e). **(d)** Integrated loss (black), determined as the temporal mean of the normalized L1-loss shown in (c), and response time (red), determined as the earliest time after the onset of input for which the normalized L1-loss drops and stays below e^{-1}, for a network with $d_{\mathrm{RF}} = 10$ ($n_{\mathrm{RF}} = 21$), $a_{\mathrm{SFA}} = 1$ and 101 values of τ_{SFA} scanned between 0 and 1. The integrated loss and response time of a network without SFA are shown by colored dots. **(e)** Integrated loss for different n_{RF} (curves are color coded as in (b)) as a function of a_{SFA} (for $\tau_{\mathrm{SFA}} = 1$). The minima, determining a^*_{SFA} used in (b), (c) and Fig 6A, are connected by a black curve, showing that the optimal adaptation strength increases for larger RFs. We note that the data in (a), (d) and (e) suggest that the optimal adaptation parameters fulfill the relation $a_{\mathrm{SFA}}\tau_{\mathrm{SFA}} \lesssim 1$. *(Caption continued on next page)*

Figure A: *(Continued)* **f)** Convergence speed in networks with SFA and inhibition. Grid scan of the integrated loss for a_{SFA} linearly scanned between 0 and a^*_{SFA} and $w^{\mathrm{rec,bal}}_{\mathrm{sum}}$ linearly scanned between 0 and $w^{\mathrm{rec,bal}}_{\mathrm{sum},c}$ (101 values each, $d_{\mathrm{RF}} = 10$ ($n_{\mathrm{RF}} = 21$), $\tau_{\mathrm{SFA}} = 1$). The white region indicates parameters for which network activity diverges, identified by an integrated loss larger than 1, cf. also (e). Here the loss was computed for the time window with present input only, more precisely for $t \in [100\tau - \mathrm{d}t, 300\tau]$, different from (a)-(e). For all simulations, we created data by simulating 1D networks with $N = 200$, $\tau = 1$ using the Euler method with step size $\mathrm{d}t = 0.01$; n_{RF}, τ_{SFA}, a_{SFA} and $w^{\mathrm{rec,bal}}_{\mathrm{sum}}$ varied as described above.

define $\delta x_i^{\mathrm{bal}} = \int_T^T \mathrm{d}t\ \delta x_i^{\mathrm{bal}}(t)$ as the integral of the postsynaptic activity changes $\delta x_i^{\mathrm{bal}}(t)$ in neuron i that are directly caused by activity changes $\Delta x_j(t)$ in neuron j through the balanced interaction (without considering how the postsynaptic changes are further affected by the recurrent net weights). With this, we define the balanced interaction strength as the proportionality factor between $\delta x_i^{\mathrm{bal}}$ and δx_j. We find that

$$\begin{aligned}
\delta x_i^{\mathrm{bal}} &= \int_{-T}^{T} \mathrm{d}t\ \frac{W_{ij}^{\mathrm{rec,bal}}}{\tau} \Delta x_j(t) && \text{(S20)}\\
&= \frac{W_{ij}^{\mathrm{rec,bal}}}{\tau} \left(\int_{-T}^{T} \mathrm{d}t\ x_j(t) - \int_{-T}^{T} \mathrm{d}t\ x_j(t - \tau_{\mathrm{lag}}) \right)\\
&= \frac{W_{ij}^{\mathrm{rec,bal}}}{\tau} \left(\int_{-T}^{T} \mathrm{d}t\ x_j(t) - \int_{-T-\tau_{\mathrm{lag}}}^{T-\tau_{\mathrm{lag}}} \mathrm{d}\tilde{t}\ x_j(\tilde{t}) \right)\\
&= \frac{W_{ij}^{\mathrm{rec,bal}}}{\tau} \left(\int_{T-\tau_{\mathrm{lag}}}^{T} \mathrm{d}t\ x_j(t) - \int_{-T-\tau_{\mathrm{lag}}}^{-T} \mathrm{d}t\ x_j(t) \right)\\
&= \frac{W_{ij}^{\mathrm{rec,bal}}}{\tau} \Big(\tau_{\mathrm{lag}} x_j(T) - \tau_{\mathrm{lag}} x_j(-T) \Big)\\
&= \frac{\tau_{\mathrm{lag}}}{\tau} W_{ij}^{\mathrm{rec,bal}}\ \delta x_j. && \text{(S21)}
\end{aligned}$$

Here we substituted $\tilde{t} = t - \tau_{\mathrm{lag}}$ in the third line; in the fourth line we used that large parts of the two integrals cancel and in the fifth line that $x_j(t)$ is constant between $T - \tau_{\mathrm{lag}}$ and T as well as between $-T - \tau_{\mathrm{lag}}$ and $-T$. The result states that a change δx_j in presynaptic activity causes postsynaptic state changes that integrate to $\frac{\tau_{\mathrm{lag}}}{\tau} W_{ij}^{\mathrm{rec,bal}}\ \delta x_j$.

This may also be intuitively understood as follows: A step-like activity change in $x_j(t_0)$ by $\delta x_j(t_0)$ at time t_0 increases $\Delta x_j(t)$ by $\delta x_j(t_0)$ for all t with $t_0 \le t \le t_0 + \tau_{\mathrm{lag}}$. According to Eq (S19) it thus changes $\dot{x}_i(t)$ by $(1/\tau) W_{ij}^{\mathrm{rec,bal}} \delta x_j(t_0)$ for a duration of τ_{lag}. The integrated effect is thus $(\tau_{\mathrm{lag}}/\tau) W_{ij}^{\mathrm{rec,bal}} \delta x_j(t_0)$. A continuous change of $x_j(t)$ may be seen as assembled of many small step-like ones, which together sum to δx_j and thus have the integrated effect Eq (S21).

In the limit of small τ_{lag}, the balanced interaction term transmits the temporal derivative of the neuronal dynamics in excitatory manner, adding a contribution with the same sign to the temporal derivative of the postsynaptic neuron. To see this, we scale the balanced weights with τ_{lag} in such a way that the total, integrated effect of the balanced interaction due to a presynaptic activity change at t is independent of the lag between excitatory and inhibitory activity, τ_{lag}, i.e., we set $W_{ij}^{\mathrm{rec,bal}} = \frac{\tau}{\tau_{\mathrm{lag}}} c_{ij}$ with constant c_{ij}, compare Eq (S21). In the limit of short lag the balanced interaction term then becomes $\lim_{\tau_{\mathrm{lag}} \to 0} W_{ij}^{\mathrm{rec,bal}} \Delta x_j(t) = \lim_{\tau_{\mathrm{lag}} \to 0} \tau c_{ij} \Delta x_j(t)/\tau_{\mathrm{lag}} = \tau c_{ij} \dot{x}_j(t)$. The prefactor $\tau c_{ij} > 0$ is positive, which renders the coupling excitatory.

G Loss evolution of balanced networks

To analytically estimate the evolution of the loss in our networks with balancing, delayed inhibition, we use again the linearized L1 loss Eq (S16), i.e., we assume again that the activity is always lower than or equal to the target activity. In the purely excitatory network, the linearized loss satisfied some simple dynamical

equations, Eq (S17) and Eq (S8). We will see in the following that this also holds in our balanced networks. To obtain the dynamical equation we compute the temporal derivative of the loss, using the linear time evolution of the activity Eqs (26) and (28). We assume that the input is constant, $r_j(t) = r_j$, and use the knowledge that for such input the dynamics converge to a stationary target state x_i^*. This yields

$$\begin{aligned}\tau \dot{L}^{\text{lin}}(t) &= \frac{\tau}{N}\sum_{i=1}^{N}\frac{\mathrm{d}}{\mathrm{d}t}\big(x_i^* - x_i(t)\big)\\ &= -\frac{1}{N}\sum_{i=1}^{N}\Big(-x_i(t) + \sum_{j=1}^{N} W_{ij}^{\text{rec,net}} x_j(t)\\ &\qquad + \sum_{j=1}^{N} W_{ij}^{\text{rec,bal}}\Delta x_j(t) + \sum_{j=1}^{N} W_{ij}^{\text{ff}} r_j\Big).\end{aligned} \tag{S22}$$

We now specialize the computation further by taking into account that in the cases of interest for us, W^{rec}, $W^{\text{rec,I}}$ and thus also $W^{\text{rec,net}}$ and $W^{\text{rec,bal}}$, as well as W^{ff} are circulant matrices. This implies that the column sums $w_{\text{sum}}^{\text{rec,E}} = \sum_{i=1}^{N} W_{ij}^{\text{rec,E}}$, $w_{\text{sum}}^{\text{rec,bal}} = \sum_{i=1}^{N} W_{ij}^{\text{rec,bal}}$, $w_{\text{sum}}^{\text{rec,net}} = \sum_{i=1}^{N} W_{ij}^{\text{rec,net}}$ and $w_{\text{sum}}^{\text{ff}} = \sum_{i=1}^{N} W_{ij}^{\text{ff}}$ are independent of the column j. Eq (S22) thus simplifies to

$$\begin{aligned}\tau \dot{L}^{\text{lin}}(t) &= \frac{1}{N}\sum_{i=1}^{N} x_i(t) - w_{\text{sum}}^{\text{rec,net}}\frac{1}{N}\sum_{j=1}^{N} x_j(t)\\ &\qquad - w_{\text{sum}}^{\text{rec,bal}}\frac{1}{N}\sum_{j=1}^{N}\Delta x_j(t) - w_{\text{sum}}^{\text{ff}}\frac{1}{N}\sum_{j=1}^{N} r_j.\end{aligned} \tag{S23}$$

The networks that we want to track analytically start with $x_i(0) = 0$, such that the initial linear loss is $L^{\text{lin}}(0) = \sum_{i=1}^{N} x_i^* - 0$. To describe the network loss dynamics, we can thus use

$$\frac{1}{N}\sum_{i=1}^{N} x_i(t) = \frac{1}{N}\sum_{i=1}^{N} x_i^* - 0 - \big(x_i^* - x_i(t)\big) = L^{\text{lin}}(0) - L^{\text{lin}}(t). \tag{S24}$$

An alike equation holds for $\frac{1}{N}\sum_{i=1}^{N} x_i(t-\tau_{\text{lag}})$, such that

$$\begin{aligned}\frac{1}{N}\sum_{i=1}^{N}\Delta x_i(t) &= \frac{1}{N}\sum_{i=1}^{N} x_i(t) - x_i(t-\tau_{\text{lag}})\\ &= L^{\text{lin}}(0) - L^{\text{lin}}(t) - \big(L^{\text{lin}}(0) - L^{\text{lin}}(t-\tau_{\text{lag}})\big)\\ &= -\big(L^{\text{lin}}(t) - L^{\text{lin}}(t-\tau_{\text{lag}})\big) = -\Delta L^{\text{lin}}(t),\end{aligned} \tag{S25}$$

where we introduced the abbreviation

$$\Delta L(t) = L^{\text{lin}}(t) - L^{\text{lin}}(t-\tau_{\text{lag}}) \tag{S26}$$

for the difference between the current and the delayed loss. Inserting Eq (S24) and Eq (S25) into Eq (S23) gives

$$\tau \dot{L}^{\text{lin}}(t) = \big(1 - w_{\text{sum}}^{\text{rec,net}}\big)\big(L^{\text{lin}}(0) - L^{\text{lin}}(t)\big) + w_{\text{sum}}^{\text{rec,bal}}\Delta L^{\text{lin}}(t) - w_{\text{sum}}^{\text{ff}}\frac{1}{N}\sum_{j=1}^{N} r_j. \tag{S27}$$

To eliminate the explicit occurrence of the inputs, we use that in the stationary state, which is reached for $t \to \infty$, we have $L^{\text{lin}}(\infty) = 0$, $\dot{L}^{\text{lin}}(\infty) = 0$ and $\Delta L^{\text{lin}}(\infty) = 0$. For $t \to \infty$, Eq (S27) thus shows that

$$0 = (1 - w_{\text{sum}}^{\text{rec,net}})L^{\text{lin}}(0) - w_{\text{sum}}^{\text{ff}}\frac{1}{N}\sum_{j=1}^{N} r_j. \tag{S28}$$

Employing this in Eq (S27) gives our final dynamical equation for the linearized loss in terms of the variable L^{lin} only,

$$\tau \dot{L}^{\mathrm{lin}}(t) = -\big(1 - w_{\mathrm{sum}}^{\mathrm{rec,net}}\big) L^{\mathrm{lin}}(t) + w_{\mathrm{sum}}^{\mathrm{rec,bal}} \Delta L^{\mathrm{lin}}(t). \tag{S29}$$

We analytically solve this 1D linear delay differential equation with the ansatz [11]

$$L^{\mathrm{lin}}(t) = L^{\mathrm{lin}}(0) \exp(-\Omega t). \tag{S30}$$

We note that

$$\Omega = \lambda + \mathrm{i}\omega \tag{S31}$$

is generally complex. The ansatz implies

$$\dot{L}^{\mathrm{lin}}(t) = -\Omega L^{\mathrm{lin}}(t), \tag{S32}$$

$$\begin{aligned}\Delta L^{\mathrm{lin}}(t) &= L^{\mathrm{lin}}(t) - L^{\mathrm{lin}}(t - \tau_{\mathrm{lag}}) = \big(1 - \exp(\Omega \tau_{\mathrm{lag}})\big) L^{\mathrm{lin}}(t) \\ &= -\big(\exp(\Omega \tau_{\mathrm{lag}}) - 1\big) L^{\mathrm{lin}}(t).\end{aligned} \tag{S33}$$

Inserting Eqs (S32) and (S33) into Eq (S29) and dividing by $-\tau L^{\mathrm{lin}}(t)$ yields

$$-\tau \Omega L^{\mathrm{lin}}(t) = -(1 - w_{\mathrm{sum}}^{\mathrm{rec,net}}) L^{\mathrm{lin}}(t) - w_{\mathrm{sum}}^{\mathrm{rec,bal}} \big(\exp(\Omega \tau_{\mathrm{lag}}) - 1\big) L^{\mathrm{lin}}(t), \tag{S34}$$

$$\Omega = \frac{1 - w_{\mathrm{sum}}^{\mathrm{rec,net}}}{\tau} + \frac{w_{\mathrm{sum}}^{\mathrm{rec,bal}}}{\tau} \big(\exp(\Omega \tau_{\mathrm{lag}}) - 1\big). \tag{S35}$$

We see immediately that in the absence of inhibition, $w_{\mathrm{sum}}^{\mathrm{rec,bal}} = 0$, we have $\Omega = \lambda = \frac{1 - w_{\mathrm{sum}}^{\mathrm{rec,net}}}{\tau} = \frac{1}{\tau_{\mathrm{resp}}}$ (cf. Eq (13)), such that the decay rate of the purely excitatory network Eq (4) is recovered, as it has to be. To solve Eq (S35) in presence of inhibition, we rewrite it as

$$\Omega \tau_{\mathrm{lag}} = \frac{\tau_{\mathrm{lag}}}{\tau_{\mathrm{resp}}} + \frac{\tau_{\mathrm{lag}}}{\tau} w_{\mathrm{sum}}^{\mathrm{rec,bal}} \big(\exp(\Omega \tau_{\mathrm{lag}}) - 1\big), \tag{S36}$$

$$\left(\Omega \tau_{\mathrm{lag}} - \frac{\tau_{\mathrm{lag}}}{\tau_{\mathrm{resp}}} + \frac{\tau_{\mathrm{lag}}}{\tau} w_{\mathrm{sum}}^{\mathrm{rec,bal}}\right) \exp(-\Omega \tau_{\mathrm{lag}}) = \frac{\tau_{\mathrm{lag}}}{\tau} w_{\mathrm{sum}}^{\mathrm{rec,bal}}. \tag{S37}$$

Substituting

$$\Omega \tau_{\mathrm{lag}} - \frac{\tau_{\mathrm{lag}}}{\tau_{\mathrm{resp}}} + \frac{\tau_{\mathrm{lag}}}{\tau} w_{\mathrm{sum}}^{\mathrm{rec,bal}} = -z, \tag{S38}$$

we obtain

$$z \exp(z) = -\frac{\tau_{\mathrm{lag}}}{\tau} w_{\mathrm{sum}}^{\mathrm{rec,bal}} \exp\left(\frac{\tau_{\mathrm{lag}}}{\tau_{\mathrm{resp}}} - \frac{\tau_{\mathrm{lag}}}{\tau} w_{\mathrm{sum}}^{\mathrm{rec,bal}}\right). \tag{S39}$$

The branches of the Lambert W function solve $z \exp(z) = \mathrm{RHS}$ for z. Applying them to Eq (S39) yields $z = W_k(\mathrm{RHS})$, where W_k denotes the kth branch and RHS the right hand side of Eq (S39),

$$z = W_k\left(-\frac{\tau_{\mathrm{lag}}}{\tau} w_{\mathrm{sum}}^{\mathrm{rec,bal}} \exp\left(\frac{\tau_{\mathrm{lag}}}{\tau_{\mathrm{resp}}} - \frac{\tau_{\mathrm{lag}}}{\tau} w_{\mathrm{sum}}^{\mathrm{rec,bal}}\right)\right). \tag{S40}$$

Resubstituting for z then gives the decay rate λ (cf. Eq (S31)) and, if an oscillation is present, the oscillation frequency ω of the linearized loss,

$$\Omega \tau_{\mathrm{lag}} = \frac{\tau_{\mathrm{lag}}}{\tau_{\mathrm{resp}}} - \frac{\tau_{\mathrm{lag}}}{\tau} w_{\mathrm{sum}}^{\mathrm{rec,bal}} - W_k\left(-\frac{\tau_{\mathrm{lag}}}{\tau} w_{\mathrm{sum}}^{\mathrm{rec,bal}} \exp\left(\frac{\tau_{\mathrm{lag}}}{\tau_{\mathrm{resp}}} - \frac{\tau_{\mathrm{lag}}}{\tau} w_{\mathrm{sum}}^{\mathrm{rec,bal}}\right)\right). \tag{S41}$$

Fig 5 and B show the relevant solutions, obtained from the branches $k = 0$ and $k = -1$.

Similarly to the dynamics of a dampened harmonic oscillator, there is a transition from an exponentially-decaying ("overdamped") regime with two decay rates to an oscillating ("underdamped") regime with a single one. At the transition point we have critical balance and inhibitory strength ("critical damping"). In the oscillatory regime it is the amplitude of the oscillation that decays exponentially. Because the actual error is always smaller or equal to the amplitude, having slightly stronger than critical balance may be optimal, see Fig 5A. The solutions corresponding to branches other than $k = 0, -1$ have markedly higher decay rates and are thus of little relevance. They also have high oscillation frequencies with periods smaller than the lag.

We note that in the case of oscillations ($\omega \neq 0$), the linearized loss Eq (S30) periodically reaches zero. This corresponds to $x_i(t) = x_i^*$ for all neurons, meaning that all neurons simultaneously go from positive to negative deviations from their target activities, or vice-versa. In the full network model, however, these transitions will occur at different times, such that when one neuron matches its target activity there are others that don't. Therefore, the minima of the oscillation are not at zero but at a final error, see Fig 5A.

We now determine the critical balance and the critical decay rate. These are determined by $z = W_k(\mathrm{RHS})$ (Eq (S40), with RHS the right hand side of Eq (S39)) having only one real solution z. This happens at the branch point $\mathrm{RHS} = -\mathrm{e}^{-1}$ where the 0th and -1st branch agree, $z = W_0(-\mathrm{e}^{-1}) = W_{-1}(-\mathrm{e}^{-1}) = -1$ [11]. We first set $\mathrm{RHS} = z\mathrm{e}^z = -\mathrm{e}^{-1}$ to obtain $w_{\mathrm{sum},c}^{\mathrm{rec,bal}}$,

$$-\frac{\tau_{\mathrm{lag}}}{\tau} w_{\mathrm{sum},c}^{\mathrm{rec,bal}} \exp\left(-\frac{\tau_{\mathrm{lag}}}{\tau} w_{\mathrm{sum},c}^{\mathrm{rec,bal}}\right) = -\exp\left(-1 - \frac{\tau_{\mathrm{lag}}}{\tau_{\mathrm{resp}}}\right), \tag{S42}$$

$$-\frac{\tau_{\mathrm{lag}}}{\tau} w_{\mathrm{sum},c}^{\mathrm{rec,bal}} = W_k\left(-\exp\left(-1 - \frac{\tau_{\mathrm{lag}}}{\tau_{\mathrm{resp}}}\right)\right), \tag{S43}$$

where we have again used a (yet unspecified) branch of the Lambert W function to solve for $w_{\mathrm{sum},c}^{\mathrm{rec,bal}}$. We know that the inhibitory strength $w_{\mathrm{sum},c}^{\mathrm{rec,I}} = -w_{\mathrm{sum},c}^{\mathrm{rec,bal}}$ must be real and negative; this can only hold for $k = 0$ or $k = -1$. It might seem surprising that we obtain two solutions here. The reason is that there are actually two critical points, see Fig Ba: one with a positive decay rate, and another with a negative decay rate, which thus describes exponential growth. We are interested only in the converging dynamics and thus choose $k = 0$, which gives rise to it: We resubstitute $\lambda^c \tau_{\mathrm{lag}} = \frac{\tau_{\mathrm{lag}}}{\tau_{\mathrm{resp}}} - \frac{\tau_{\mathrm{lag}}}{\tau} w_{\mathrm{sum},c}^{\mathrm{rec,bal}} - z$ (Eq (S38) with Ω replaced by the critical, real decay rate λ^c), use $z = -1$ and insert the critical balance strength Eq (S43) to obtain the critical decay rate,

$$\lambda^c \tau_{\mathrm{lag}} = \frac{\tau_{\mathrm{lag}}}{\tau_{\mathrm{resp}}} - \frac{\tau_{\mathrm{lag}}}{\tau} w_{\mathrm{sum},c}^{\mathrm{rec,bal}} + 1 \tag{S44}$$

$$= 1 + \frac{\tau_{\mathrm{lag}}}{\tau_{\mathrm{resp}}} + W_k\left(-\exp\left(-1 - \frac{\tau_{\mathrm{lag}}}{\tau_{\mathrm{resp}}}\right)\right). \tag{S45}$$

As anticipated, this is positive for $k = 0$ and negative for $k = -1$. We therefore henceforth insert $k = 0$. The response time of the balanced network is the inverse of λ^c,

$$\tau_{\mathrm{resp}}^{\mathrm{bal},c} = \frac{1}{\frac{1}{\tau_{\mathrm{lag}}} + \frac{1}{\tau_{\mathrm{resp}}} + \frac{1}{\tau_{\mathrm{lag}}} W_0\left(-\exp\left(-1 - \frac{\tau_{\mathrm{lag}}}{\tau_{\mathrm{resp}}}\right)\right)}. \tag{S46}$$

We now want to derive a more easily interpretable approximation for λ^c and $\tau_{\mathrm{resp}}^{\mathrm{bal},c}$ for small $\tau_{\mathrm{lag}}/\tau_{\mathrm{resp}}$. To

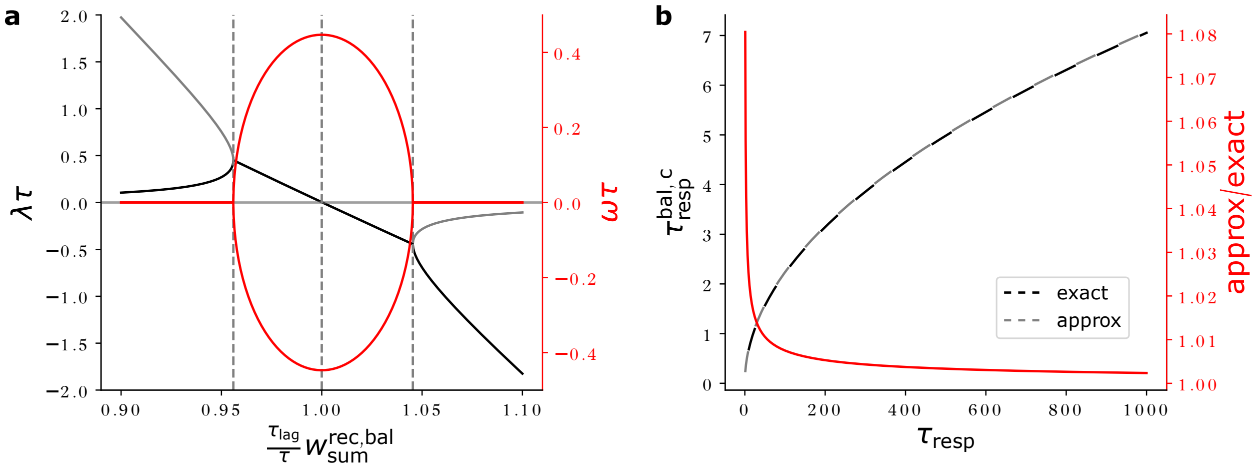

Figure B: **Critical points and approximation of the analytical solution for $\tau_{resp}^{bal,c}$. (a)** Real part (decay rate λ, black/gray) and imaginary part (oscillation frequency ω times ± 1, red) of the complex frequency of the exponential loss evolution, scaled by τ (compare Fig 5). The leftmost dashed gray vertical line marks the first critical balance strength at which the complex frequency has no imaginary part yet but there is already only a single, positive decay rate. For $(\tau_{lag}/\tau)w_{sum}^{rec,bal} \approx 1 + \tau_{lag}/3\tau_{resp}$, the decay rate is zero and transitions from positive (decaying) to negative (growing). This maximum value is slightly stronger than 1, marked by the middle dashed gray vertical line, because of the stabilizing effect of the contracting dynamics of the unbalanced network. There is a second critical balance strength, marked by the rightmost dashed gray vertical line, at which the complex frequency is real and there is a single, negative decay rate. The first critical point, with decaying dynamics, corresponds to the solution of Eq (S43) with $k = 0$, the second one, with growing dynamics, to that with $k = -1$. **(b)** Exact (black dashed, cf. Eq (S46)) and approximate (gray dashed, cf. Eq (S55)) values of the critical decay time of the balanced network, $\tau_{resp}^{bal,c}$, as a function of that of the excitatory network. Approximation and exact solution agree well. Their ratio (red) is close to one, and approaches one for $\tau_{resp} \gg \tau_{lag}$. Parameters: $\tau = 1$, $\tau_{lag} = 0.1$ and, in a), $w_{sum}^{rec,net} = 0.99$.

this end, we modify Eq (S45) to

$$\lambda^c \tau_{\text{lag}} - 1 - \frac{\tau_{\text{lag}}}{\tau_{\text{resp}}} = W_0 \left(- \exp \left(-1 - \frac{\tau_{\text{lag}}}{\tau_{\text{resp}}} \right) \right) \tag{S47}$$

$$\Rightarrow \qquad \left(\lambda^c \tau_{\text{lag}} - 1 - \frac{\tau_{\text{lag}}}{\tau_{\text{resp}}} \right) \exp \left(\lambda^c \tau_{\text{lag}} - 1 - \frac{\tau_{\text{lag}}}{\tau_{\text{resp}}} \right) = - \exp \left(-1 - \frac{\tau_{\text{lag}}}{\tau_{\text{resp}}} \right) \tag{S48}$$

$$\Leftrightarrow \qquad \left(\lambda^c \tau_{\text{lag}} - 1 - \frac{\tau_{\text{lag}}}{\tau_{\text{resp}}} \right) \exp \left(\lambda^c \tau_{\text{lag}} \right) = -1. \tag{S49}$$

Here we have applied the inverse of W_0, $z \to z e^z$, to both sides, and multiplied with $\exp(-1 - \tau_{\text{lag}}/\tau_{\text{resp}})$. Next we assume that $\lambda^c \tau_{\text{lag}}$ is small, meaning that the response time is much larger than the E-I lag. This also implies that $\tau_{\text{lag}}/\tau_{\text{resp}}$, with the response time τ_{resp} of the network without delayed inhibition, is small, since the balanced interaction speeds up responses. We then expand the exponential up to the second power in $\lambda^c \tau_{\text{lag}}$ and neglect powers higher than two in $\lambda^c \tau_{\text{lag}}$ and $\tau_{\text{lag}}/\tau_{\text{resp}}$ in the resulting polynomial,

$$\left(\lambda^c \tau_{\text{lag}} - 1 - \frac{\tau_{\text{lag}}}{\tau_{\text{resp}}} \right) \left(1 + \lambda^c \tau_{\text{lag}} + \frac{1}{2} (\lambda^c \tau_{\text{lag}})^2 + \dots \right) = -1, \tag{S50}$$

$$\lambda^c \tau_{\text{lag}} - 1 - \frac{\tau_{\text{lag}}}{\tau_{\text{resp}}} + (\lambda^c \tau_{\text{lag}})^2 - \lambda^c \tau_{\text{lag}} - \frac{\tau_{\text{lag}}}{\tau_{\text{resp}}} \lambda^c \tau_{\text{lag}} - \frac{1}{2} (\lambda^c \tau_{\text{lag}})^2 \approx -1, \tag{S51}$$

$$\frac{1}{2} (\lambda^c \tau_{\text{lag}})^2 - \frac{\tau_{\text{lag}}}{\tau_{\text{resp}}} (\lambda^c \tau_{\text{lag}}) - \frac{\tau_{\text{lag}}}{\tau_{\text{resp}}} \approx 0. \tag{S52}$$

This can be solved for $\lambda^c \tau_{\text{lag}}$,

$$\lambda^c_{\pm} \tau_{\text{lag}} \approx \frac{\tau_{\text{lag}}}{\tau_{\text{resp}}} \pm \sqrt{ \left(\frac{\tau_{\text{lag}}}{\tau_{\text{resp}}} \right)^2 + 2 \frac{\tau_{\text{lag}}}{\tau_{\text{resp}}} }, \tag{S53}$$

where only the positive solution makes sense. Since $\tau_{\text{lag}}/\tau_{\text{resp}}$ is small, we neglect in the radicand of Eq (S53)'s RHS the quadratic term compared to the linear one. Compared to the resulting square root term we further neglect the first, linear RHS term, such that we obtain

$$\lambda^c \tau_{\text{lag}} \approx \sqrt{2 \frac{\tau_{\text{lag}}}{\tau_{\text{resp}}}}. \tag{S54}$$

The response time of the balanced network follows as the inverse of λ^c,

$$\tau_{\text{resp}}^{\text{bal},c} \approx \sqrt{\frac{\tau_{\text{resp}} \tau_{\text{lag}}}{2}}. \tag{S55}$$

Fig B shows that, despite the simple formula, the quality of the approximation is very good.

H Initial response of balanced networks

In the following we explain the discrepancy between the analytical and numerical linearized loss evolution, which results in different response times (see Fig 6A, C). It stems from different initializations of neuronal activity: For this, we first note that an exponential function Eq (S30), $L^{\text{lin}}(t) = L^{\text{lin}}(0) \exp(-\Omega t)$, with Ω solving Eq (S35) and arbitrary amplitude $L^{\text{lin}}(0)$, is an eigenmode of the time evolution of the linearized loss Eq (S29), as the time evolution preserves its functional form. The results of the last section describe the loss evolution if the system is in such an eigenmode. However, in the main text simulations, networks are not initialized in an eigenmode: They are initialized with zero activity and respond to a sudden jump in the

input. This corresponds to an initial state with $x(t) = 0$ for $t \leq 0$; in particular $\Delta x(0) = 0$. Therefore both the initial and earlier losses equal the maximal loss, $L(t) = L(-\tau_{\text{lag}}) = |x^*|_1/N$ for $t \leq 0$ and $\Delta L(0) = 0$, see Eqs (S25) and (S26), Fig Cb. In contrast, for the eigenmodes we have $\Delta L^{\text{lin}}(t) = -(\exp(\Omega\tau_{\text{lag}}) - 1)L^{\text{lin}}(t)$ for $t \leq 0$ (from Eqs (S26) and (S30)); especially $\Delta L^{\text{lin}}(0) < 0$. The loss is thus not initialized in an eigenmode; the decay rate of the loss converges to that of the slowest decaying eigenmode over time.

For the chosen initial conditions, the loss decay speeds up during this process, see Fig 6c and Fig Ca, b. This can be understood as follows: In our network simulations, activity initially increases, such that $\Delta x_i(t)$ is positive. The amplifying current term $W^{\text{rec,bal}}\Delta x(t)$ ($W_{ij}^{\text{rec,bal}} \geq 0$) in Eq (S22) is thus positive, which causes a dynamical speed-up in the activity increase. Its sum is proportional to $-\Delta L(t)$, which is initially zero. The term $w_{\text{sum}}^{\text{rec,bal}}\Delta L^{\text{lin}}(t) \leq 0$ introduces the dynamical speedup evoked by $\Delta x_i(t)$ into the equation of the loss dynamics, where it causes a faster decay of the loss. As both $\Delta x_i(t)$ and $|\Delta L(t)|$ increase, the terms that generate the speedup in the activity and in the loss dynamics increase in absolute value. As a consequence, the activity increases faster and the loss decays faster. The inset in Fig Cb shows that $|\Delta L|$ increases (initially in absolute, later in relative terms) and approaches the same proportionality to L as for the slowest decaying eigenmode, which is non-oscillatory in the displayed example. This means that the system settles in the eigenmode. The loss curves then become parallel lines on the logarithmic axes (extrapolation of Fig Ca).

This different initialization and the resulting initially lower impact of lagged interactions explain why the red data points in Fig 6 show longer response times than expected from the theory.

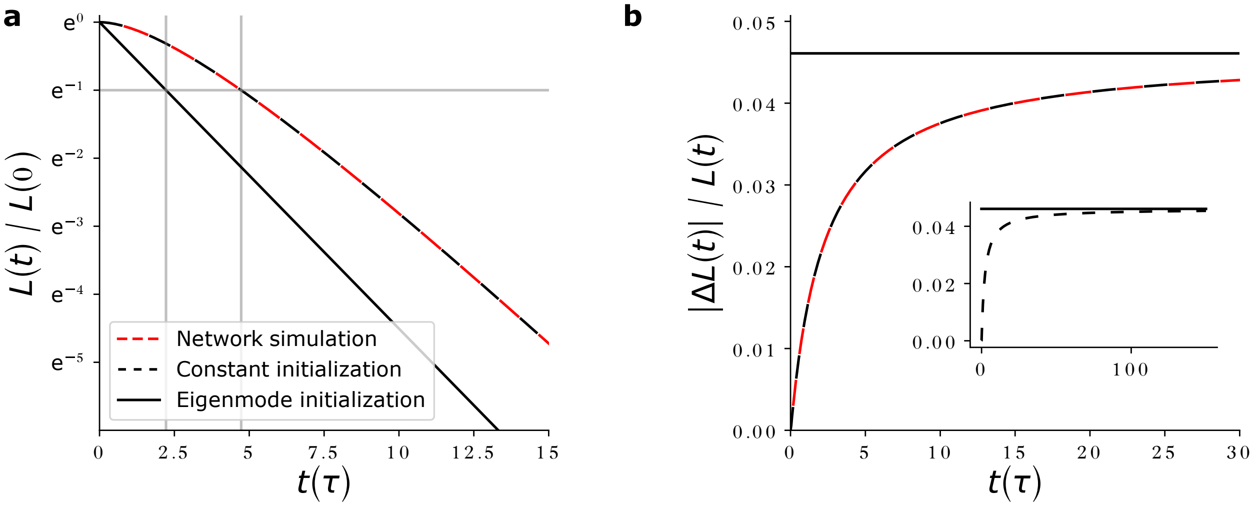

Figure C: **Effects of initial conditions on loss evolution. (a)** Normalized loss evolution obtained by solving Eq (S29) (black curves) for critical balance. The loss is either initialized in the slowest eigenmode (solid, analytical solution of Eq (S29) using Eqs (S30) and (S35)) or it is initialized as a constant function (dashed, $L(t) = L(0)$ for $t \leq 0$, numerical solution of Eq (S29)), like in our network simulations. Vertical lines indicate the time at which the error drops below e^{-1}, which experimentally defines the response time. The numerical solution (black dashed) with constant initialization describes the loss evolution of a simulated network (red dashed, $N = 200$) well. Independent of the initialization, with time the loss decay rates approach the same value; the dashed and continuous curves become parallel. **(b)** In the – for the chosen parameters non-oscillatory – slowest decaying eigenmode, $\Delta L(t)$ is always proportional to $L(t)$ (solid horizontal line at the proportionality factor $1 - e^{\lambda^c\tau_{\text{lag}}} \approx -0.046$). With constant initialization (dashed curves), $\Delta L(t)$ is initially zero, such that the impact of the balanced interactions mediated by $w_{\text{sum}}^{\text{rec,bal}}$ (see Eq (S29)) is initially small. $\Delta L(t)$ then tends to the same proportionality to $L(t)$ as for the eigenmode initialization. Inset: loss evolution for long times. Parameters: $w_{\text{sum}}^{\text{rec,net}} = 0.99$, $\tau = 1$, $\tau_{\text{lag}} = 0.1$, $w^{\text{rec,bal}} = 0.5w_{\text{sum},c}^{\text{rec,bal}}$; we thus have $\frac{\tau_{\text{lag}}}{\tau}w_{\text{sum}}^{\text{rec,bal}} \approx 0.95594$; the displayed slowest decaying eigenmode is non-oscillatory, cf. Fig B.

I Leaky integrator inhibition

The more conventional dynamics of the inhibitory neurons as leaky integrators of excitatory neurons (henceforth: leaky integrator inhibition),

$$\tau_{\mathrm{I}} \dot{x}^{\mathrm{I}}_j(t) = -x^{\mathrm{I}}_j(t) + x^{\mathrm{E}}_j(t), \tag{S56}$$

means that inhibitory activity still tracks excitatory activity, and, due to the compared to τ_{I} long time scales of the changes in $x^{\mathrm{E}}_j(t)$, does so with a time lag approximately given by τ_{I}. This is qualitatively similar to our model choice (henceforth: delay inhibition) where $x^{\mathrm{I}}_j(t) = x^{\mathrm{E}}_j(t - \tau_{\mathrm{lag}})$ with a correspondence of τ_{I} and τ_{lag}. One difference is that the exact delay does not dampen high-frequency components of the excitatory activity and always introduces the same time shift. In contrast, the leaky integration acts as a low-pass filter: it dampens high frequency components. Further it shifts low frequency input components by τ_I, but high frequency ones by the constant phase $\pi/2$ and thus by a lesser extent in time.

In this study, the balanced networks were analyzed at the critical balance that yields the fastest convergence to the steady state, which is exactly at the onset of fluctuations, see Fig Ba. In the entire convergent non-oscillatory regime, the response time $\tau^{\mathrm{bal}}_{\mathrm{resp}}$, which characterizes the time scale of the network dynamics, is a large multiple of the lag τ_{lag}. This holds especially at the critical balance. Therefore we do not expect qualitative and only small quantitative changes between the two choices for the inhibitory dynamics.

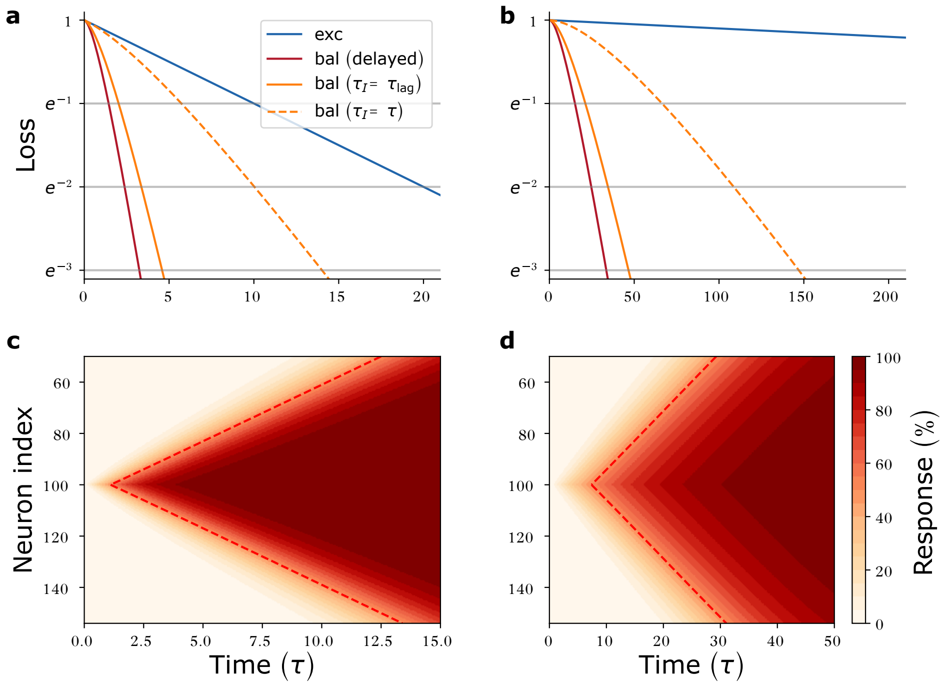

Figure D: **Balanced cooperatively coding networks evolve qualitatively similarly whether inhibition is delayed or leakily integrates excitation. (a)**, **(b)** show the evolution of the L1-loss for a narrow 1D RF ((a), $\tau_{\mathrm{resp}} = 10$) and a wide RF ((b), $\tau_{\mathrm{resp}} = 1000$). Note the differently scaled x-axes. The response time increases by a factor of 100 for the unbalanced and by a factor of about 10 for the balanced networks. **(c)**, **(d)** Activity propagates in a network with leaky integrator inhibition from the site of an isolated constant input at neuron index 100 in the two settings. The input is switched on at $t = 0$. The red dashed line marks the times at which feature neurons achieve 50% of their steady state activation. Parameters: $N = 200$, $\tau = 1$, $dt = 0.01$, $\tau_{\mathrm{lag}} = 0.1$, $w^{\mathrm{rec,net}} = 0.5 \cdot 1/(1 - 1/\tau_{\mathrm{resp}})$ where $\tau_{\mathrm{resp}} = 10$ (a) or $\tau_{\mathrm{resp}} = 1000$ (b). For the balanced networks with leaky integrator inhibition, $w^{\mathrm{rec,bal}}_{\mathrm{leaky}} = 0.5 - \gamma_{\mathrm{I}}(0.5 - w^{\mathrm{rec,bal}}_{\mathrm{delayed},c})$, i.e., $w^{\mathrm{rec,bal}}_{\mathrm{leaky}}$ is a factor γ_{I} further away from the maximum admissible balance strength (0.5) than the critically-tuned lagged inhibition network, with $\gamma_{\mathrm{I}} = 1.4$. This factor was roughly optimized.

Fig D confirms our expectation with numerical simulations comparing: (i) a purely excitatory network, (ii) a balanced network with delay inhibition, (iii) a balanced network with leaky integrator inhibition and time constant $\tau_{\mathrm{I}} = \tau_{\mathrm{lag}}$ and (iv) a balanced network with leaky integrator inhibition and time constant $\tau_I = \tau$. Panels (a) and (c) show results for a small RF for which the response time of the unbalanced network is $\tau_{\mathrm{resp}} = 10$; panels (b) and (d) use $\tau_{\mathrm{resp}} = 1000$. The balanced networks with leaky integrator inhibition are roughly tuned to a critical balance at the onset of oscillations, which is close to but slightly weaker than that for the network with delay inhibition.

For integration time constants matching the delay, $\tau_{\mathrm{I}} = \tau_{\mathrm{lag}}$, we observe that the network response is close to but slightly slower than that of the delay inhibition network. This holds both for narrow and wide RFs on both ends of the spectrum tested in this work. The observation indicates that the scaling with RF size remains the same, irrespective of the particulars of the delayed inhibitory tracking of excitation. More concretely, comparing Fig Da and b, the response times of all three balanced networks increase by the same factor of about 10, while that of the unbalanced network increases 100-fold.

Fig Dc and d show how activity (normalized by the final, steady-state activity) evolves in a network with leaky integrator inhibition in response to an isolated input that is switched on at $t = 0$ (compare Fig 3B). The dynamics are qualitatively similar to those of the delayed-inhibition network.

J Stationary solution for 2D stimulus

To determine its stationary solution, we rewrite the network dynamics for cooperatively encoding a 2D stimulus, Eq (39), as

$$\begin{aligned}\tau \dot{x}_{ij} = &- (1 - 4w^{\mathrm{rec,2D}})x_{ij} + w^{\mathrm{rec,2D}}\big(\ (x_{i+1,j} - 2x_{ij} + x_{i-1,j}) \\ &+ (x_{i,j+1} - 2x_{ij} + x_{i,j-1})\big) + w^{\mathrm{ff,2D}} r_{ij}.\end{aligned} \tag{S57}$$

For wide RFs, the discrete values of x_{ij} can be approximated by a continuous function $x(y, z)$ that is evaluated at the points $y, z = i, j$. The difference $x_{i+1,j} - 2x_{ij} + x_{i-1,j}$ is then approximated by the second-order partial derivative $\partial_y^2 x(y, z)$, and analogously for the second spatial dimension. The stationary solution then satisfies

$$\underbrace{\frac{1 - 4w^{\mathrm{rec,2D}}}{w^{\mathrm{rec,2D}}}}_{=\tilde{\lambda}^2} x(y, z) - \nabla^2 x(y, z) = \underbrace{\frac{w^{\mathrm{ff,2D}}}{w^{\mathrm{rec,2D}}}}_{=\tilde{\gamma}} r(y, z). \tag{S58}$$

Fourier transforming and solving for $X = \mathcal{F}(x)$ yields

$$X(k_y, k_z) = \frac{\tilde{\gamma}}{\tilde{\lambda}^2 + |k|^2} \cdot R(k_y, k_z), \tag{S59}$$

where X and R are the Fourier transforms of x and r, respectively. The inverse Fourier transform $\mathcal{F}^{-1}$ of this product is the convolution of the inverse Fourier transforms,

$$x(y, z) = \left(\mathcal{F}^{-1} \left(\frac{\tilde{\gamma}}{\tilde{\lambda}^2 + |k|^2} \right) \ * \ r \right) (y, z), \tag{S60}$$

with $\mathcal{F}^{-1}(\tilde{\gamma}/(\tilde{\lambda}^2 + |k|^2)$ acting as the Green's function. The Fourier transform is known and given by (e.g. p.66 of [12])

$$\mathcal{F}^{-1} \left(\frac{\tilde{\gamma}}{\tilde{\lambda}^2 + |k|^2} \right) (y, z) = \frac{\tilde{\gamma}}{2\pi} K_0 \left(\tilde{\lambda} \rho(y, z) \right), \tag{S61}$$

where $\rho(y, z) = \sqrt{y^2 + z^2}$ and K_0 is the zeroth modified Bessel function of the second kind. The Green's function describes the (network) response to an isolated input at the origin, and the RF is hence given by

(Eq (41))

$$\mathrm{RF}_{ijkl} = \frac{1}{2\pi}\frac{w^{\mathrm{ff,2D}}}{w^{\mathrm{rec,2D}}}\; K_0\left(\sqrt{\frac{1-4w^{\mathrm{rec,2D}}}{w^{\mathrm{rec,2D}}}}\rho_{ijkl}\right), \tag{S62}$$

where $\rho_{ijkl} = \sqrt{|i-k|^2 + |j-l|^2}$.

K Tuning spiking networks for cooperative coding

Excitatory network A diffusion approximation [13] of the synaptic inputs in our purely excitatory spiking network Eq (47) yields

$$\tau_m \dot{v}_{ik} = -v_{ik} + I^i_{\mathrm{ext}} + J_{\mathrm{EE}}\tau_m \sum_{j=i-1}^{i+1} x_j + \sqrt{2\tau_m}\sigma\xi_{ik}(t), \tag{S63}$$

where we assume that each neuron has a fixed indegree K_{EE}, $J_{\mathrm{EE}} = K_{\mathrm{EE}}w_{\mathrm{EE}}$ and x_i denotes the average firing rate of neurons in population i ("population rate"). We omit the variance of the recurrent spiking input, which is negligible compared to the external Gaussian white noise strength σ. In the stationary state, all population rates must thus satisfy the self-consistency condition

$$x_i = f_\sigma\left(I^i_{\mathrm{ext}} + J_{\mathrm{EE}}\tau_m \sum_{j=i-1}^{i+1} x_j\right), \tag{S64}$$

where

$$f_\sigma(\mu) = \left(\tau_{\mathrm{ref}} + \sqrt{\pi}\tau_m \int_{\frac{\mu - V_{\mathrm{thr}}}{\sqrt{2}\sigma}}^{\frac{\mu - V_{\mathrm{reset}}}{\sqrt{2}\sigma}} \mathrm{e}^{y^2}\,\mathrm{erfc}(y)\,\mathrm{d}y\right)^{-1} \tag{S65}$$

denotes the transfer function of an LIF neuron under Gaussian white noise of strength σ [13]. In the following, we will approximate f_σ by a threshold-linear function

$$f_\sigma(\mu) \approx f(\mu) \coloneqq f_0 + g(\mu - \mu_0)\Theta(\mu - \mu_0) \tag{S66}$$

with gain g, rheobase μ_0, and baseline rate f_0.

We now assume that population 0 receives strong feedforward input, while all other populations $i \neq 0$ receive a lower background input,

$$I^i_{\mathrm{ext}} = \begin{cases} I^{\mathrm{on}}_{\mathrm{ext}}, & i = 0 \\ I^{\mathrm{off}}_{\mathrm{ext}}, & i \neq 0. \end{cases} \tag{S67}$$

The cooperatively coding network should respond to such a stimulation with a rate profile

$$x_i = x_{\mathrm{max}}\gamma^{|i|} + x_b, \tag{S68}$$

with baseline rate $x_b \geq f_0$ and maximal rate $x_{\mathrm{max}} + x_b$. As before, $\gamma = \exp(-1/d)$ depends on the RF size $n_{\mathrm{RF}} = 2d + 1$.

In the following we obtain an analytical expression for the synaptic coupling strength J_{EE} and the feedforward inputs $I^{\mathrm{off}}_{\mathrm{ext}}$, $I^{\mathrm{on}}_{\mathrm{ext}}$ that induce such an RF. We neglect boundary effects. The rate profile $\{x_i\}_i$ can only be a steady-state of the network activity if all feature populations i satisfy the self-consistency condition Eq (S64). For the unstimulated populations $i \neq 0$ we get

$$x_i = f\left(I^{\mathrm{off}}_{\mathrm{ext}} + \tau_m J_{\mathrm{EE}} \sum_{j=i-1}^{i+1} x_j\right). \tag{S69}$$

Since all feature population rates are larger than $x_b \geq f_0$ we can invert the threshold-linear transfer function Eq (S66) and derive from Eq (S69)

$$J_{\mathrm{EE}} = \frac{f^{-1}(x_i) - I_{\mathrm{ext}}^{\mathrm{off}}}{\tau_m \sum_{j=i-1}^{i+1} x_j} = \frac{x_i - f_0 + g(\mu_0 - I_{\mathrm{ext}}^{\mathrm{off}})}{g\tau_m \sum_{j=i-1}^{i+1} x_j} \quad \forall\, i \neq 0 \tag{S70}$$

Expressing the rate of feature population i as a function of the rate of its neighbor $i' = \mathrm{sgn}(i)(|i|+1)$: $x_i = (x_{i'} - (1-\gamma)x_b)/\gamma$, yields

$$J_{\mathrm{EE}} = \frac{x_{i'} - (1-\gamma)x_b - \gamma(f_0 - g(\mu_0 - I_{\mathrm{ext}}^{\mathrm{off}}))}{g\tau_m \sum_{j=i'-1}^{i'+1}[x_j - (1-\gamma)x_b]}. \tag{S71}$$

On the other hand, evaluating Eq (S70), which must hold for all populations $i \neq 0$, for $i = i'$ yields

$$J_{\mathrm{EE}} = \frac{x_i' - f_0 + g(\mu_0 - I_{\mathrm{ext}}^{\mathrm{off}})}{g\tau_m \sum_{j=i'-1}^{i'+1} x_j}. \tag{S72}$$

To ensure the equality of Eqs S71 and S72 (i.e., there must be a single weight J_{EE} such that the selfconsistency condition is fulfilled at all non-stimulated populations) we must require a baseline rate of

$$x_b = 0 \tag{S73}$$

($\Rightarrow x_i = x_{\max}\gamma^{|i|} = x_{i'}/\gamma$) and $f_0 = g(\mu_0 - I_{\mathrm{ext}}^{\mathrm{off}})$. Since $x_b \geq f_0 \geq 0$, a zero baseline rate implies $f_0 = 0$, and the background stimulation must be

$$I_{\mathrm{ext}}^{\mathrm{off}} = \mu_0, \tag{S74}$$

which brings all neurons to the approximate rheobase. The self-consistent synaptic weight is then given by

$$J_{\mathrm{EE}} \overset{(\mathrm{S70})}{=} \frac{x_i}{g\tau_m \sum_{j=i-1}^{i+1} x_j} = \frac{1}{g\tau_m \sum_{j=-1}^{1} \gamma^j}. \tag{S75}$$

Finally we choose the required stimulation strength $I_{\mathrm{ext}}^{\mathrm{on}}$ such that Eq (S64) is also fulfilled for the stimulated population $i = 0$:

$$x_{\max} \overset{!}{=} x_0 \overset{(\mathrm{S66})}{=} f\left(I_{\mathrm{ext}}^{\mathrm{on}} + \tau_m J_{\mathrm{EE}} \sum_{j=-1}^{1} x_j\right). \tag{S76}$$

$$\begin{aligned} \Leftrightarrow \quad I_{\mathrm{ext}}^{\mathrm{on}} &= f^{-1}(x_{\max}) - \tau_m J_{\mathrm{EE}} \sum_{j=-1}^{1} x_j = \mu_0 + \frac{x_{\max}}{g} - \frac{x_{\max}\sum_{j=-1}^{1}\gamma^{|j|}}{g\sum_{j=-1}^{1}\gamma^j} \\ &= \mu_0 + \frac{x_{\max}}{g}\left[\frac{1-\gamma^2}{1+\gamma+\gamma^2}\right], \end{aligned} \tag{S77}$$

where we employed Eq (S66), Eq (S68) and Eq (S75) in the second line. We further optimize J_{EE} numerically to find a close match between the target and simulated RF size n_{RF}.

Balanced network We now describe the tuning of the balanced network (Eq (48), Eq (49)) for an RF of size n_{RF} with a peak rate $x_{\max}$. Every inhibitory population i receives synaptic input from its partner excitatory population with a weight w_{IE} tuned such that the stationary rate x_i^I of the inhibitory population is approximately equal to the rate x_i^E of its excitatory partner population:

$$w_{\mathrm{IE}} = \frac{J_{\mathrm{IE}}}{p_{\mathrm{IE}} N_E} \quad \text{with } J_{\mathrm{IE}} = \frac{f^{-1}(x_i^E) - I_{\mathrm{ext}}^{\mathrm{off}}}{\tau_m x_i^E} = \frac{x_i^E - f_0 + g(\mu_0 - I_{\mathrm{ext}}^{\mathrm{off}})}{g\tau_m x_i^E} = \frac{1}{g\tau_m}, \tag{S78}$$

where we again assumed $f_0 = 0$ and $I_{\text{ext}}^{\text{off}} = \mu_0$ (cf. Eqs (S73) and (S74). The (numerically optimized) excitatory coupling (Fig 8C, black circles) can then be increased beyond the optimal level:

$$\bar{w}_{\text{EE}} = \frac{\bar{J}_{\text{EE}}}{K_{\text{EE}}} \quad \text{with } \bar{J}_{\text{EE}} = sJ_{\text{EE}},\ s > 1. \tag{S79}$$

The increase in excitatory coupling is balanced by feedback projections from the inhibitory neurons: Every inhibitory population projects back to its excitatory partner population, as well as to its two neighbors (see Fig 10A). The synaptic weights are adjusted such that the net excitatory weight between populations remains optimal for the given RF size. In particular, in the stationary regime we have

$$\tau_m \dot{v}_{ik}^E \approx -v_{ik}^E + I_{\text{ext}}^i + \underbrace{\left(\bar{J}_{\text{EE}} + J_{\text{EI}}\right)}_{\stackrel{!}{=} J_{\text{EE}}} \tau_m \sum_{j=i-1}^{i+1} x_j^E + \sqrt{2\tau_m}\sigma\xi_{ik}(t). \tag{S80}$$

$$\Rightarrow \quad w_{\text{EI}} = \frac{J_{\text{EI}}}{p_{\text{EI}} N_I} \quad \text{with } J_{\text{EI}} = J_{\text{EE}} - \bar{J}_{\text{EE}} < 0. \tag{S81}$$

To get a close match to the target RF size we must again numerically optimize (upregulate $|w_{\text{EI}}|$ within a range of [0,1.2]%). This is likely due to the imperfect match of inhibitory stationary firing rates, which are slightly lower than their excitatory counterparts.

L Stability of cooperative coding in spiking networks

Numerical integration of network dynamics close to instability. To estimate the stability of our cooperative spiking networks, we resort to our approximate description of their rate dynamics

$$\tau\dot{r} = -r + f\left(I_{\text{ext}} + \tau_m J_{\text{EE}} C r\right) \tag{S82}$$

where $r \in \mathbb{R}_{\geq 0}^{N_F}$ denotes the firing rates of N_F excitatory feature populations, $f(\mu) = g(\mu - \mu_0)\Theta(\mu - \mu_0)$ is the threshold-linear approximation of the transfer function, and the connectivity of feature populations is given by the circulant adjacency matrix

$$C = \begin{pmatrix} 1 & 1 & 0 & 0 & \dots & 0 & 1 \\ 1 & 1 & 1 & 0 & \dots & 0 & 0 \\ & & & & \ddots & & \\ 1 & 0 & 0 & \dots & 0 & 1 & 1 \end{pmatrix}. \tag{S83}$$

For simplicity we consider here the purely excitatory network. Since the summed feedforward and recurrent synaptic inputs are at all times above the rheobase μ_0, the dynamics are linear

$$\tau\dot{r} = -r + g\tau_m J_{\text{EE}} C r + g(I_{\text{ext}} - \mu_0) \tag{S84}$$

and the rates have a stable fixed point if the real part of the largest eigenvalue of $g\tau_m J_{\text{EE}} C$ is smaller than 1.

By design, the eigenvalues of the analytically tuned networks approach this instability point for increasing RF size (Fig Ea). Due to the high time step sensitivity of the numerical integration described in the Methods and illustrated in Fig Eb, a numerical optimization of the weights J_{EE} for larger target RF sizes can overestimate the required weight, or even push some eigenvalues across the instability point (see insets in Fig Ea). Thus, when an optimized network is simulated with a smaller discretization time step, deviations in the RF size or even an instability of the network dynamics can become apparent (Fig Ec).

We confirmed in simulations with stochastic Heun instead of Euler-Maruyama integration that the observed time step sensitivity is indeed due to the grid-based processing of spikes and synaptic delta-pulses, and not the numerical integration method itself.

We used a default time step of dt=10μs throughout this paper. Decreasing the time step further has no strong influence in networks tuned to $n_{\text{RF}} \leq 7$, and a mild influence in networks tuned to $n_{\text{RF}} = 9, 11$ (see Fig Eb, c). For larger RFs, the networks that are numerically optimized using the default time step become unstable when simulated at smaller time steps (Fig Ec). We thus only show results up to $n_{\text{RF}} \leq 11$. We expect that our qualitative results hold for larger RF sizes. Integrating these network dynamics accurately merely requires much smaller time steps.

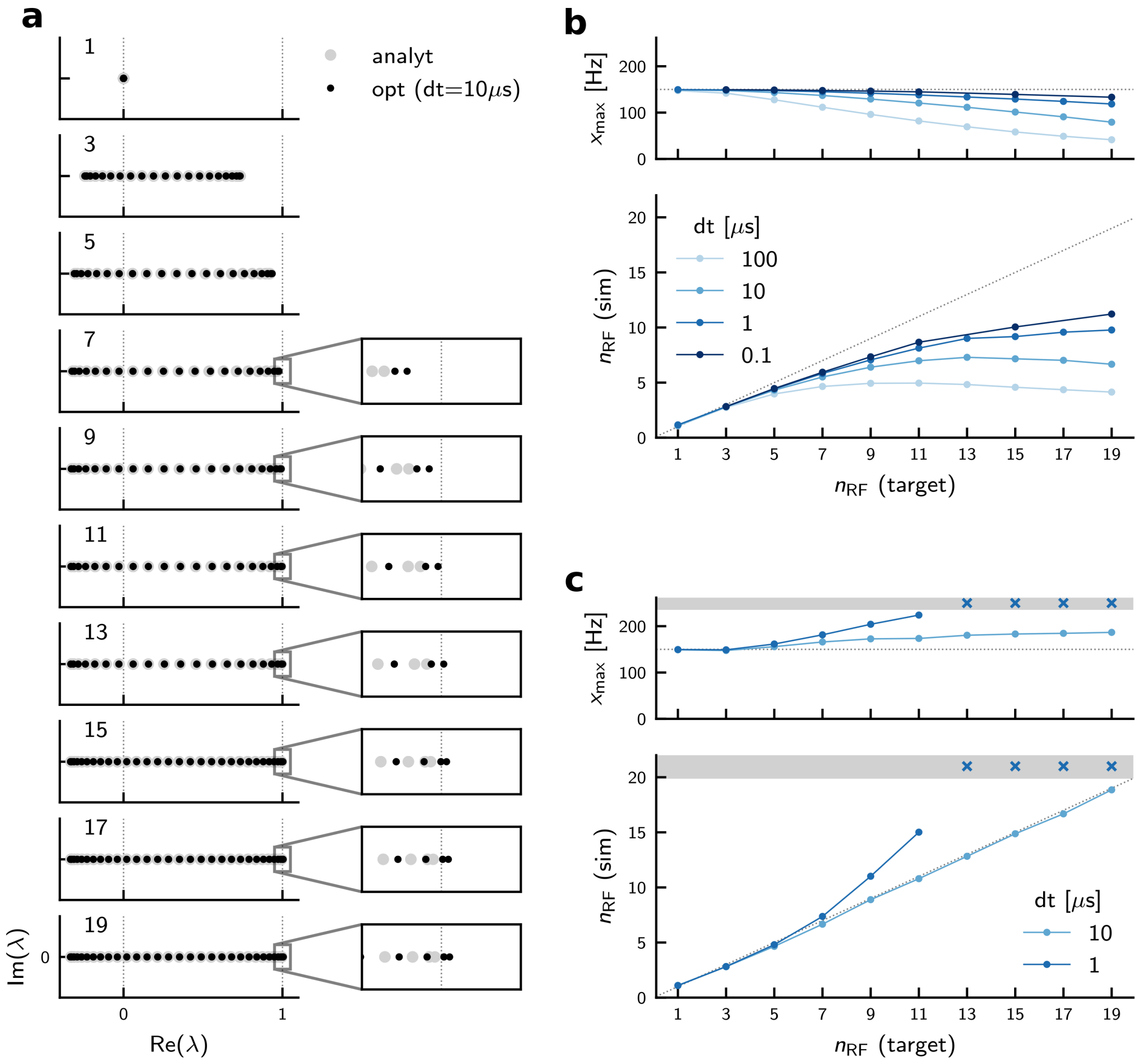

Figure E: **Numerical integration accuracy for increasing RF size.** **(a)** Eigenvalues λ of the population coupling matrix $g\tau_m J_{\mathrm{EE}}C$ in the analytically tuned networks (gray, independent of dt), and in networks tuned numerically using dt=10μs (black). Numbers in top left corner indicate target RF size n_{RF}. Insets highlight eigenvalues with real parts crossing the instability point of 1 for networks optimized numerically for RFs larger than 11. **(b)** Simulations of the analytically tuned networks (gray eigenvalues in a) for decreasing numerical integration time step dt. Top: Peak rate. Bottom: RF size. **(c)** Simulations of the numerically optimized networks (using dt=10μs, black eigenvalues in a), using either the original optimization time step (dt=10μs, light blue), or a smaller time step (dt=1μs, darker blue). Crosses mark simulations for which the network is unstable. Otherwise same layout as (b). The networks shown in this figure are the same that are shown in Fig 8.

The above-described time step sensitivity can already be observed in most simple networks close to the instability point, such as a single population of spiking neurons coupled with an excitatory weight $J_{\rm EE}^{\rm max} = (g\tau_m)^{-1}$. Fig F demonstrates this by simulations of a single population of 500 spiking neurons (Eq (47)) with excitatory within-population coupling strength $J_{\rm EE} = sJ_{\rm EE}^{\rm max}$ approaching the instability point (scaling factors $s = 0.7, 0.9, 0.99$, columns in Fig F). We simulate each network for a range of time step sizes dt=1μs, 10μs, 100μs and feedforward drives $I_{\rm ext} \in [2{\rm mV}, 15{\rm mV}]$. The closer the network is to the instability point, the stronger the dependence of the stationary firing rates on the size of the time step.

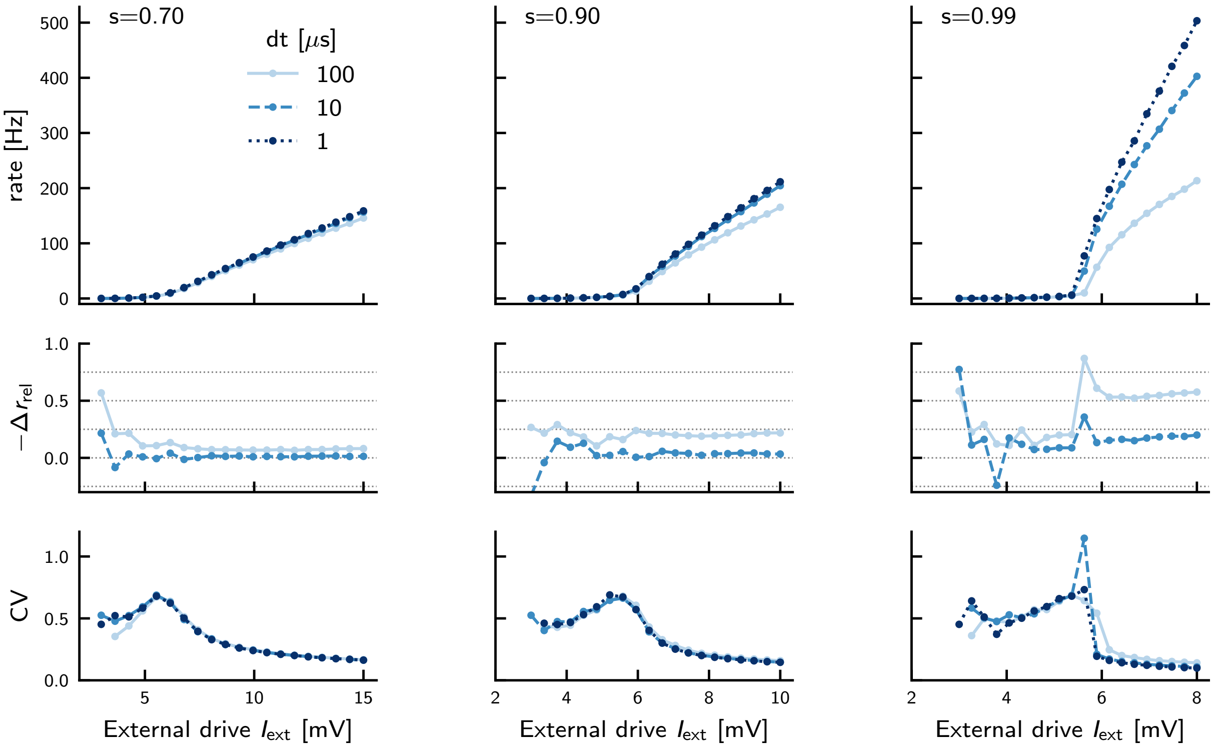

Figure F: **Sensitive dependence of simulation results on the numerical integration accuracy in a single population of neurons close to instability.** Recurrent excitatory coupling strength increases from left to right column as $J_{\rm EE} = sJ_{\rm EE}^{\rm max}$. Each column shows simulation results for three different time steps dt and a range of feedforward drives. Close to the instability (right hand side column), simulations with different time step sizes yield very different stationary rates. Top: stationary population firing rate measured by averaging the spiking activity between 1–3 seconds after stimulus onset. We note that for $s = 0.99$, intermediate input levels around rheobase and small time step size, we sometimes observe sudden activity changes reminiscent of metastable switches, rendering the notion of stationary activity problematic. Middle: negative relative difference $-\Delta r_{\rm rel}$ between the firing rate measured at time step dt and the firing rate measured at the finest time step dt $= 1\mu$s: $-\Delta r_{\rm rel} = (r_{{\rm dt}=1\mu s} - r_{\rm dt})/r_{{\rm dt}=1\mu s}$. Values for small drive are unreliable due to the small rates. Bottom: Coefficient of variation of interspike intervals averaged across all neurons. Peaks of CV around rheobase reflect presumable metastable switches.

Tuning of recurrent synaptic weights. In order to observe wide RFs in the spiking network, we had to numerically fine-tune the synaptic coupling strength. For such RFs already small deviations in the synaptic weights induce large changes in the RF size; the cooperatively coding network becomes very sensitive to changes in mean synaptic weight (Fig G).

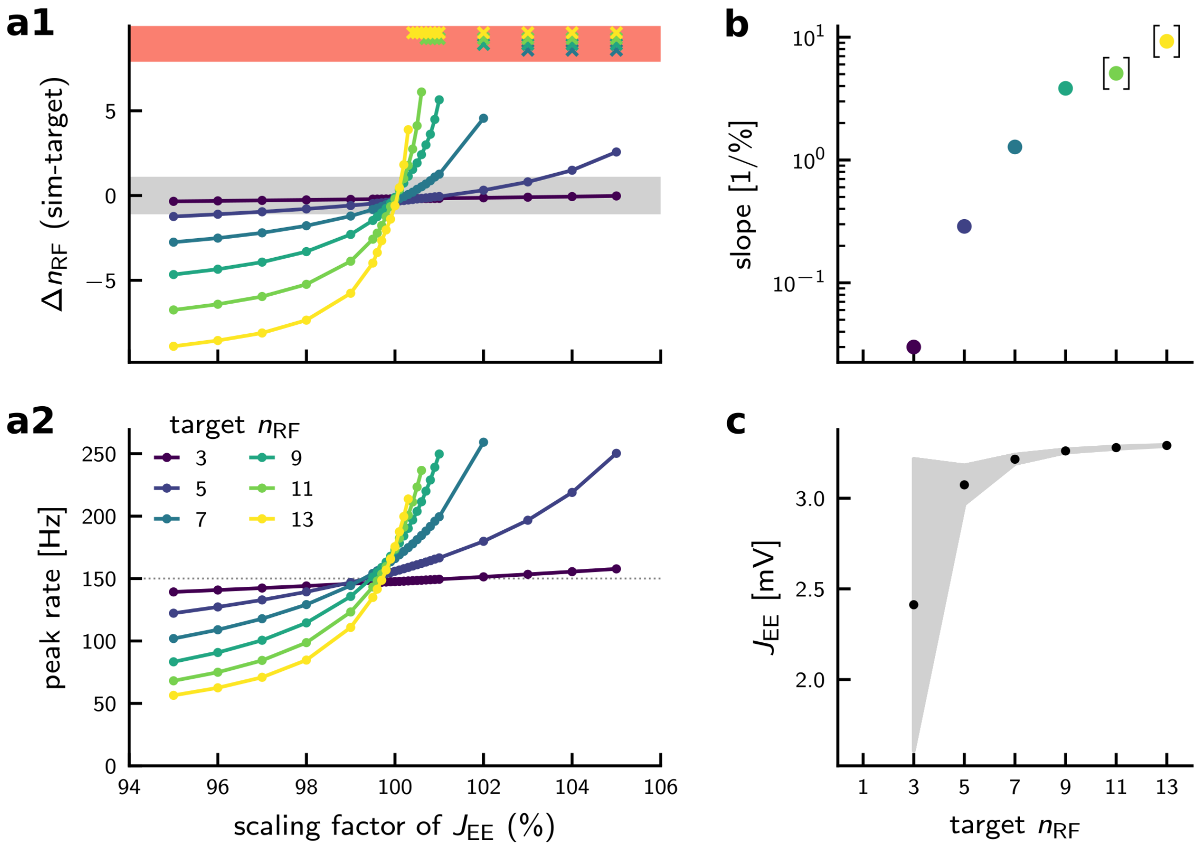

Figure G: **Sensitivity analysis of cooperative coding.** **(a)** Spiking network simulations with excitatory coupling strength J_{EE} varied by a factor $s \in [95, 105]\%$. Every dot marks one simulation. **(a1)** Deviation of simulated RF size from target; colors specify different RF target sizes (see legend in (a2)). Gray area: target $n_{RF} \pm 1$. Red area: simulations with unstable rates: activity grows in all populations and no longer matches the target profile. Simulations are aborted once the baseline firing rates exceed 40 Hz. **(a2)** Peak firing rate measured in stimulated population. Dashed horizontal line marks analytical target $x_{max} = 150$Hz. **(b)** Slope of RF deviation in (a1) around $s = 100\%$, approximated as the average of the slopes of the two neighboring line segments. Slopes in brackets are affected by the inaccuracy of numerical integration with time step 0.01 ms for large field sizes (see Fig Ec) and should be interpreted with caution. **(c)** Optimized weight J_{EE} (black dots, cf. Fig 8C) and range of weights (gray area) that yield an RF deviating less than ±1 from the target value.

Fluctuations induced by finite size. For the spiking networks shown in the main text, we used large feature populations, fixed recurrent indegree and a constant mean FF drive, to reduce finite size fluctuations in the rate dynamics. Here we show how the dynamics of the purely excitatory networks of Fig 8 change when relaxing these assumptions: (1) feature populations are smaller ($N_E = 500$); (2) the feedforward input is given as Poisson spike trains that are drawn randomly from input pools of size $N_x = 500$ with probability $p_x = 0.1$, superposed and scaled by synaptic weights $w_{\rm FF} = 0.05$ mV; (3) all indegrees (FF and recurrent) are variable due to random Erdős-Rényi connectivity. For each target RF we simulate 20 random realizations of the network, i.e., with different connectivity, voltage initialization, and noise realizations, Fig Ha. As expected, the fluctuations in the population rates are larger, Fig Hb, top. Nevertheless, the network maintains a stable rate response close to the target profile. For increasing RF size we observe a widening of the simulated RFs beyond the target (Fig Ha, $\sim n_{\rm RF} = 9, 11$), and for $n_{\rm RF} \geq 13$ rate instabilities can occur.

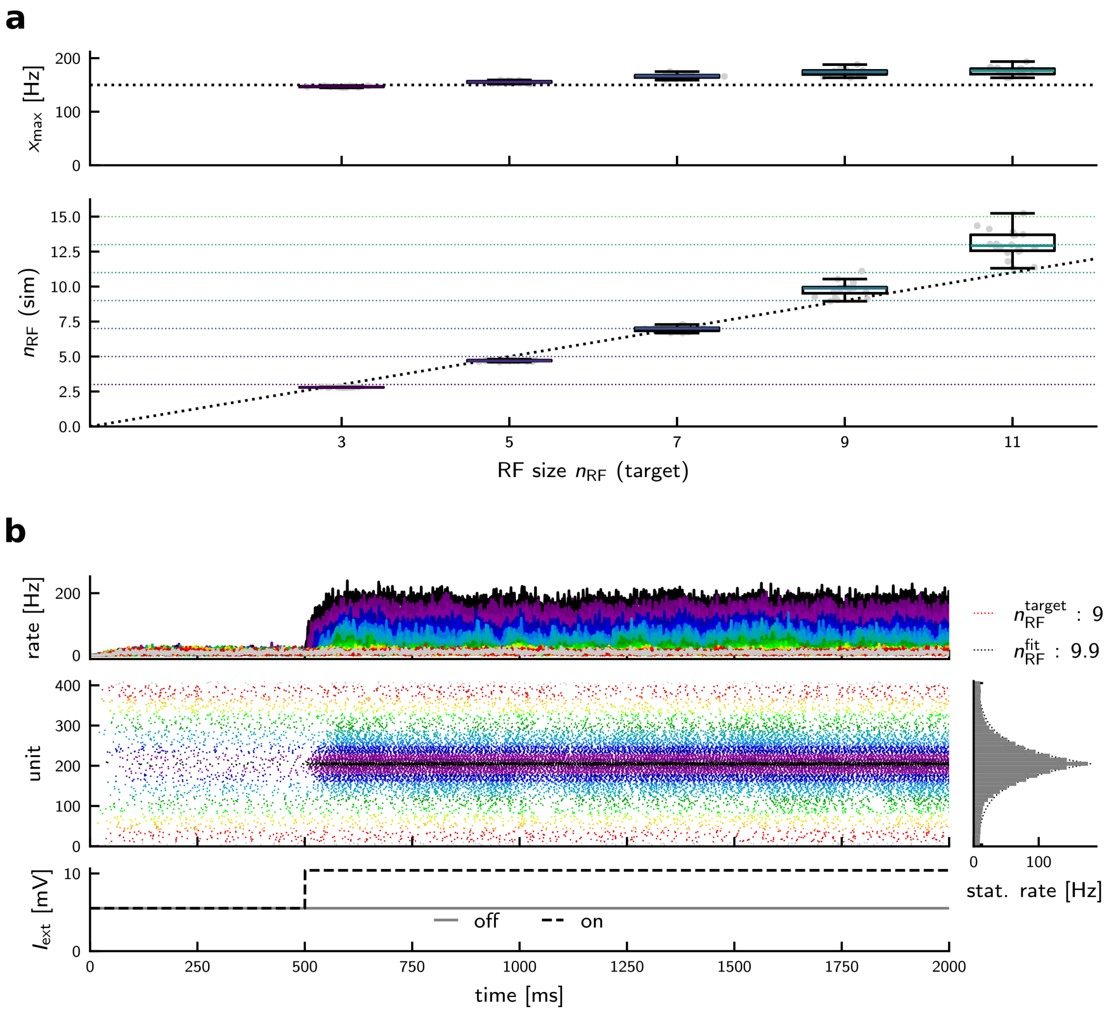

Figure H: **Cooperative coding with fluctuations. (a)**. The numerically optimized networks shown in Fig 8E are resimulated with reduced population size ($N_E = 500$), random Erdős-Rényi connectivity, and Poisson feedforward input as described above. For each target RF size $n_{\rm RF} \in \{3, 5, 7, 9, 11\}$ 20 random realizations are shown. Top: stationary firing rate of the stimulated population (peak of the RF). Bottom: RF size in simulation. Box marks Q1-Q3. Horizontal lines indicate median. Gray dots mark individual simulation results. **(b)** Example network simulation for target $n_{\rm RF} = 9$. Shown is the random seed that resulted in an RF closest to the median shown in (a). Same layout as Fig 8E.

M Cooperative coding in spiking networks with homogeneous E/I populations

Here we describe a second set of spiking network models (E-only and EI); their dynamics are shown in Fig I. The main differences to the models of the main text are that also inhibitory neurons receive tuned feedforward input, and all within-feature population coupling strengths remain fixed independent of the target RF. Furthermore, we explore a different noise model, by scaling the strength of the Gaussian white noise as the square root of the mean feedforward drive.

Excitatory network model

In the purely excitatory network (Fig Ia1) the membrane potential of a neuron k in feature population i is given by

$$\begin{aligned}\tau_m \dot{v}_{ik}^E &= -v_{ik}^E + I_{\text{ext}}^i + \sqrt{2\tau_m}\sigma_{\text{ext}}^i \xi_{ik}(t) \\ &\quad + w_{\text{EE}}^{\text{in}} \tau_m \sum_{\Gamma_{ik}^{\text{EE,in}}} \sum_{\hat{t}} \delta\left(t - \hat{t} - \Delta^E\right) + w_{\text{EE}}^{\text{out}} \tau_m \sum_{\Gamma_{ik}^{\text{EE,out}}} \sum_{\hat{t}} \delta\left(t - \hat{t} - \Delta^E\right). \end{aligned} \tag{S85}$$

The strength of the Gaussian white noise depends on the mean feedforward input, as would be expected from a diffusion approximation of (independent) Poisson spiking inputs arriving via feedforward synapses of strength w_{FF} at some rate λ:

$$\tau_m \dot{v}_{ik}^E = -v_{ik}^E + w_{\text{FF}} \tau_m \sum_{\hat{t}_\lambda} \delta(t - \hat{t}_\lambda) \approx -v_{ik}^E + \underbrace{w_{\text{FF}} \tau_m \lambda}_{I_{\text{ext}}^i} + \underbrace{w_{\text{FF}} \tau_m \sqrt{\lambda}}_{\sqrt{2\tau_m}\sigma_{\text{ext}}^i} \xi_{ik}(t) \tag{S86}$$

The noise strength thus scales as the square root of the mean feedforward input

$$\sigma_{\text{ext}}^i = \sqrt{\frac{w_{\text{FF}}}{2} I_{\text{ext}}^i}. \tag{S87}$$

As before, neurons receive synaptic inputs from their own feature population ($\sim \Gamma_{ik}^{\text{EE,in}}$), as well as from their two neighboring feature populations ($\sim \Gamma_{ik}^{\text{EE,out}}$). Synaptic connections within feature populations now have a fixed strength $w_{\text{EE}}^{\text{in}}$. Only synaptic connection weights $w_{\text{EE}}^{\text{out}}$ across feature populations are tuned to obtain the desired RF size. Table 1 summarizes all parameter values.

RF size in the excitatory network

We tune the network to obtain an RF of size n_{RF} and peak rate $x_{\max}$ using the same scheme as for our first set of spiking networks and hence keep its description short: A diffusion approximation of the synaptic inputs in Eq (S85) yields

$$\tau_m \dot{v}_{ik} = -v_{ik} + I_{\text{ext}}^i + J_{\text{EE}}^{\text{in}} \tau_m x_i + J_{\text{EE}}^{\text{out}} \tau_m \left[x_{i-1} + x_{i+1}\right] + \sqrt{2\tau_m}\sigma_{\text{ext}}^i \xi_{ik}(t). \tag{S88}$$

For simplicity we assume that the single neuron transfer function is given by

$$f_{\text{LIF}}(\mu) = \left(\tau_{\text{ref}} + \sqrt{\pi}\tau_m \int_{\frac{\mu - V_{\text{thr}}}{\sqrt{2}\sigma(\mu)}}^{\frac{\mu - V_{\text{reset}}}{\sqrt{2}\sigma(\mu)}} \mathrm{e}^{y^2} \operatorname{erfc}(y) \, \mathrm{d}y \right)^{-1} \tag{S89}$$

with $\sigma(\mu) = \sqrt{w_{\text{FF}}\mu/2}$ [13]. If the neuron is driven predominantly by feedforward input ($\mu \approx I_{\text{ext}}^i$, $x_i \approx 0$, i.e., before stimulus onset), $\sigma(\mu)$ is an accurate approximation of the noise level as it would be expected from Poisson inputs (cf. Eq (S87)). If there is a prominent contribution of recurrent input, the scaling of the noise with the feedforward weight w_{FF} is not exact, but a useful approximation for determining the

recurrent weights self-consistently. This holds, in particular, because for large mean input, the mean firing rate does not depend strongly on the the exact noise level. Again, we approximate the transfer function by a threshold-linear function

$$f(\mu) \coloneqq g(\mu - \mu_0)\Theta(\mu - \mu_0) \tag{S90}$$

(Fig. Ib). In the stationary state, all rates need to satisfy the self-consistency condition

$$x_i = f\left(I_{\text{ext}}^i + J_{\text{EE}}^{\text{in}}\tau_m x_i + J_{\text{EE}}^{\text{out}}\tau_m \left[x_{i-1} + x_{i+1}\right]\right). \tag{S91}$$

For unstimulated populations ($i \neq 0$) to fulfill the self-consistency condition Eq (S91) given a target profile $x_i = x_{\max}\gamma^{|i|}$, we need an across-population coupling strength

$$J_{\text{EE}}^{\text{out}} = \frac{\left(1 - \tau_m g J_{\text{EE}}^{\text{in}}\right) x_i}{\tau_m g \left[x_{i-1} + x_{i+1}\right]} = \frac{1 - \tau_m g J_{\text{EE}}^{\text{in}}}{\tau_m g \left[\gamma^{-1} + \gamma\right]}. \tag{S92}$$

To have positive $J_{\text{EE}}^{\text{out}} > 0$ we need $J_{\text{EE}}^{\text{in}} < 1/(\tau_m g)$. We choose

$$J_{\text{EE}}^{\text{in}} = \frac{1}{3\tau_m g}, \tag{S93}$$

such that for large RFs the across-coupling strength converges toward the within-coupling strength from below:

$$J_{\text{EE}}^{\text{out}} = \frac{1 - \tau_m g J_{\text{EE}}^{\text{in}}}{\tau_m g \left[\gamma^{-1} + \gamma\right]} = \frac{2}{3\tau_m g \left[\gamma^{-1} + \gamma\right]} \overset{\gamma \to 1}{\longrightarrow} \frac{1}{3\tau_m g} = J_{\text{EE}}^{\text{in}}. \tag{S94}$$

Again we choose the required stimulation strength $I_{\text{ext}}^{\text{on}}$ such that the self-consistency condition Eq (S91) is also fulfilled for the stimulated population $i = 0$:

$$x_{\max} \overset{!}{=} x_0 = f\left(I_{\text{ext}}^{\text{on}} + J_{\text{EE}}^{\text{in}}\tau_m x_{\max} + 2J_{\text{EE}}^{\text{out}}\tau_m x_{\max}\gamma\right) \tag{S95}$$

$$I_{\text{ext}}^{\text{on}} = f^{-1}\left(x_{\max}\right) - J_{\text{EE}}^{\text{in}}\tau_m x_{\max} - 2J_{\text{EE}}^{\text{out}}\tau_m x_{\max}\gamma \tag{S96}$$

$$\overset{\substack{\text{(S90)}\\ \text{(S92)}}}{=} \mu_0 + \frac{x_{\max}}{g} - J_{\text{EE}}^{\text{in}}\tau_m x_{\max} - 2\frac{1 - \tau_m g J_{\text{EE}}^{\text{in}}}{g\left[\gamma^{-1} + \gamma\right]} x_{\max}\gamma \tag{S97}$$

$$= \mu_0 + \frac{x_{\max}}{g}\left[1 - J_{\text{EE}}^{\text{in}}\tau_m g\right]\left[1 - \frac{2\gamma}{\left[\gamma^{-1} + \gamma\right]}\right] \tag{S98}$$

$$= \mu_0 + \frac{x_{\max}}{g}\left[1 - J_{\text{EE}}^{\text{in}}\tau_m g\right]\frac{1 - \gamma^2}{1 + \gamma^2} \overset{\text{(S93)}}{=} \mu_0 + \frac{2x_{\max}}{3g}\frac{1 - \gamma^2}{1 + \gamma^2}. \tag{S99}$$

As for the excitatory network model presented in the main text (Fig 8), the analytical tuning works well for small RF sizes up to $n_{\text{RF}} \sim 5$. To achieve larger RF sizes we additionally numerically optimize $J_{\text{EE}}^{\text{out}}$.

Excitatory-inhibitory network model

To obtain the excitatory-inhibitory network model we augment each feature neuron population of the model Eq (S85) by an inhibitory population of size N_I with the same input, noise and output rate characteristics as

the excitatory population. Specifically, the network dynamics are given by

$$\begin{aligned}\tau_m \dot{v}^E_{ik} = & -v^E_{ik} + I^i_{\text{ext}} + \sqrt{2\tau_m}\sigma^i_{\text{ext}}\xi^E_{ik}(t) \\ & + w^{\text{in}}_{\text{EE}}\tau_m \sum_{\Gamma^{\text{EE,in}}_{ik}} \sum_{\hat{t}} \delta\left(t - \hat{t} - \Delta^E\right) + w^{\text{out}}_{\text{EE}}\tau_m \sum_{\Gamma^{\text{EE,out}}_{ik}} \sum_{\hat{t}} \delta\left(t - \hat{t} - \Delta^E\right) \\ & + w^{\text{in}}_{\text{EI}}\tau_m \sum_{\Gamma^{\text{EI,in}}_{ik}} \sum_{\hat{t}} \delta\left(t - \hat{t} - \Delta^I\right) + w^{\text{out}}_{\text{EI}}\tau_m \sum_{\Gamma^{\text{EI,out}}_{ik}} \sum_{\hat{t}} \delta\left(t - \hat{t} - \Delta^I\right), \end{aligned} \tag{S100}$$

$$\begin{aligned}\tau_m \dot{v}^I_{ik} = & -v^I_{ik} + I^i_{\text{ext}} + \sqrt{2\tau_m}\sigma^i_{\text{ext}}\xi^I_{ik}(t) \\ & + w^{\text{in}}_{\text{IE}}\tau_m \sum_{\Gamma^{\text{IE,in}}_{ik}} \sum_{\hat{t}} \delta\left(t - \hat{t} - \Delta^E\right) + w^{\text{out}}_{\text{IE}}\tau_m \sum_{\Gamma^{\text{IE,out}}_{ik}} \sum_{\hat{t}} \delta\left(t - \hat{t} - \Delta^E\right) \\ & + w^{\text{in}}_{\text{II}}\tau_m \sum_{\Gamma^{\text{II,in}}_{ik}} \sum_{\hat{t}} \delta\left(t - \hat{t} - \Delta^I\right) + w^{\text{out}}_{\text{II}}\tau_m \sum_{\Gamma^{\text{II,out}}_{ik}} \sum_{\hat{t}} \delta\left(t - \hat{t} - \Delta^I\right). \end{aligned} \tag{S101}$$

Every EI feature population is designed to be balanced and inhibition-dominated in itself, with equal excitatory and inhibitory rates (cf. "Model A" of [14]): Excitatory and inhibitory neurons have the same biophysical parameters and receive the same feedforward inputs and the same synaptic inputs from neighboring populations (on average). The coupling strengths within the EI feature populations are fixed with excitatory weights $w^{\text{in}}_{\text{EE}} = w^{\text{in}}_{\text{IE}}$ and inhibitory weights $w^{\text{in}}_{\text{EI}} = w^{\text{in}}_{\text{II}} = -cw^{\text{in}}_{\text{EE}}$ (inhibition-dominated: $c = 5 > N_E/N_I$, [14]). The across population coupling is tuned to obtain the target RF. We note that, while we include in the model a potential inhibitory coupling across populations ($\sim w^{\text{out}}_{\text{EI}}, w^{\text{out}}_{\text{II}}$), for simplicity we generally leave it out in our simulations (cf. Fig Ia2). Table 1 summarizes all parameter values used in the simulations.

RF size in the excitatory-inhibitory network

When tuning the EI spiking network presented in the Main Text (see section K), we derived a self-consistency condition based on the transfer function of a single, uncoupled neuron. In the current model the within-population coupling is fixed. Thus, we can (numerically) determine the transfer function of a neuron embedded in its EI-node (Fig Ib, red dots). Note that by construction (Model A of [14]) the firing rates of the inhibitory and excitatory subpopulations are the same. We fit a threshold linear function to this transfer function,

$$f(\mu) \coloneqq g\,(\mu - \mu_0)\,\Theta(\mu - \mu_0). \tag{S102}$$

Since excitatory and inhibitory populations have the same stationary rates ($x^E_i = x^I_i =: x_i$) and incoming excitatory/inhibitory weights ($w^{\text{out}}_{\text{EE}} = w^{\text{out}}_{\text{IE}} =: w^{\text{out}}_{\text{E}} \geq 0$, $w^{\text{out}}_{\text{EI}} = w^{\text{out}}_{\text{II}} =: w^{\text{out}}_{\text{I}} \leq 0$), the self-consistency conditions for the excitatory and inhibitory stationary rates are equivalent:

$$x_i \overset{!}{=} f\left(I^i_{\text{ext}} + \tau_m\left[K_E w^{\text{out}}_{\text{E}}\left[x_{i-1} + x_{i+1}\right] + K_I w^{\text{out}}_{\text{I}}\left[x_{i-1} + x_{i+1}\right]\right]\right). \tag{S103}$$

Note that within-population coupling is already accounted for in the transfer function f. Given the same connection probability for all pathways, the inhibitory indegree satisfies $K_I = \alpha K_E$, where $\alpha = N_I/N_E = 0.25$ denotes the ratio of inhibitory to excitatory population sizes. Eq (S103) thus reduces to

$$x_i = f\left(I^i_{\text{ext}} + \tau_m K_E w^{\text{out}}_{\text{E}}\left[1 - \alpha c\right]\left[x_{i-1} + x_{i+1}\right]\right), \tag{S104}$$

where $c = |w^{\text{out}}_{\text{I}}|/w^{\text{out}}_{\text{E}} \geq 0$ is the ratio of the inhibitory and the excitatory across-population coupling strength. In our coding scheme the net input from neighboring populations is positive, which implies

$$c \overset{!}{<} 1/\alpha = 4. \tag{S105}$$

For simplicity, we omit inhibitory cross-population coupling in our simulations ($c = 0 \Rightarrow w^{\text{out}}_{\text{I}} = 0$ mV). We checked exemplarily that inhibitory cross-coupling with $0 < c < 4$ did not change our results qualitatively.

For purely excitatory across-population coupling ($c = 0$), the self-consistency condition in unstimulated populations ($i \neq 0, I_{\text{ext}}^{i} = I_{\text{ext}}^{\text{off}}$) yields an excitatory weight strength

$$w_{\text{E}}^{\text{out}} = \frac{f^{-1}(x_i) - I_{\text{ext}}^{\text{off}}}{\tau_m K_E \left[x_{i-1} + x_{i+1}\right]} = \frac{x_i + g(\mu_0 - I_{\text{ext}}^{\text{off}})}{g\tau_m K_E \left[x_{i-1} + x_{i+1}\right]} \tag{S106}$$

$$= \frac{x_i}{g\tau_m K_E \left[x_{i-1} + x_{i+1}\right]} \quad \forall\, |i| > 0 \tag{S107}$$

$$= \frac{\gamma}{g\tau_m K_E \left[1 + \gamma^2\right]}, \tag{S108}$$

where we used Eq (S104) and Eq (S102) in the first, and $I_{\text{ext}}^{\text{off}} = \mu_0$ in the second line. For the stimulated population ($i = 0, I_{\text{ext}}^{i} = I_{\text{ext}}^{\text{on}}$) the self-consistency condition and Eq (S108) yield

$$I_{\text{ext}}^{\text{on}} = f^{-1}(x_{\max}) - 2\tau_m K_E w_{\text{E}}^{\text{out}} x_{\max} \gamma \approx \mu_0 + \frac{x_{\max}}{g}\left[1 - 2g\tau_m K_E w_{\text{E}}^{\text{out}} \gamma\right] \tag{S109}$$

$$= \mu_0 + \frac{x_{\max}}{g} \frac{1 - \gamma^2}{1 + \gamma^2}. \tag{S110}$$

Again we optimize $w_{\text{E}}^{\text{out}}$ numerically to achieve a good fit of RF sizes $n_{\text{RF}} > 5$. We note that also in this model the numerically integrated dynamics depends sensitively on the discretization time step when the network is close to the instability point (cf. Fig E). Thus, quantitative results for large RFs should be interpreted with caution.

Parameter	Value	Definition
N_F	41	Number of feature populations
N_E	4000	Excitatory neurons per feature population
p_{EE}	0.1	Connection probability from E to E
$w_{\mathrm{EE}}^{\mathrm{in}}$	$\sim 8\ \mu$V	Synaptic weight within excitatory populations
$w_{\mathrm{EE}}^{\mathrm{out}}$	Eq (S108)	Synaptic weight across excitatory populations
V_{thr}	10 mV	Spike threshold
V_{rest}	0 mV	Resting voltage
V_{reset}	0 mV	Reset voltage
τ_m	20 ms	Membrane time constant
τ_{ref}	0 ms	Refractory period
Δ^E	$\sim \mathcal{U}(0,2)$ ms	Delay of excitatory synapses
w_{FF}	0.5 mV	Synaptic feedforward weight from input to feature layer
dt	0.01 ms	Simulation time step
N_I	1000	Inhibitory neurons per feature population
p_{EI}	0.1	Connection probability from I to E
p_{IE}	0.1	Connection probability from E to I
$w_{\mathrm{IE}}^{\mathrm{in}}$	$w_{\mathrm{EE}}^{\mathrm{in}}$	Synaptic weight E to I within populations
$w_{\mathrm{EI}}^{\mathrm{in}}$	$-5w_{\mathrm{EE}}^{\mathrm{in}}$	Synaptic weight I to E within populations
$w_{\mathrm{II}}^{\mathrm{in}}$	$-5w_{\mathrm{EE}}^{\mathrm{in}}$	Synaptic weight I to I within populations
$w_{\mathrm{IE}}^{\mathrm{out}}$	$w_{\mathrm{EE}}^{\mathrm{out}}$	Synaptic weight E to I across populations
Δ^I	$\sim \mathcal{U}(0,1)$ ms	Delay of inhibitory synapses

Table 1: **Parameters of homogeneous spiking network model** Top: excitatory-only network (Fig Ia1). Bottom: additional parameters for balanced network (Fig Ia2). Inhibitory neurons have the same biophysical parameters as excitatory neurons (top part of the table).

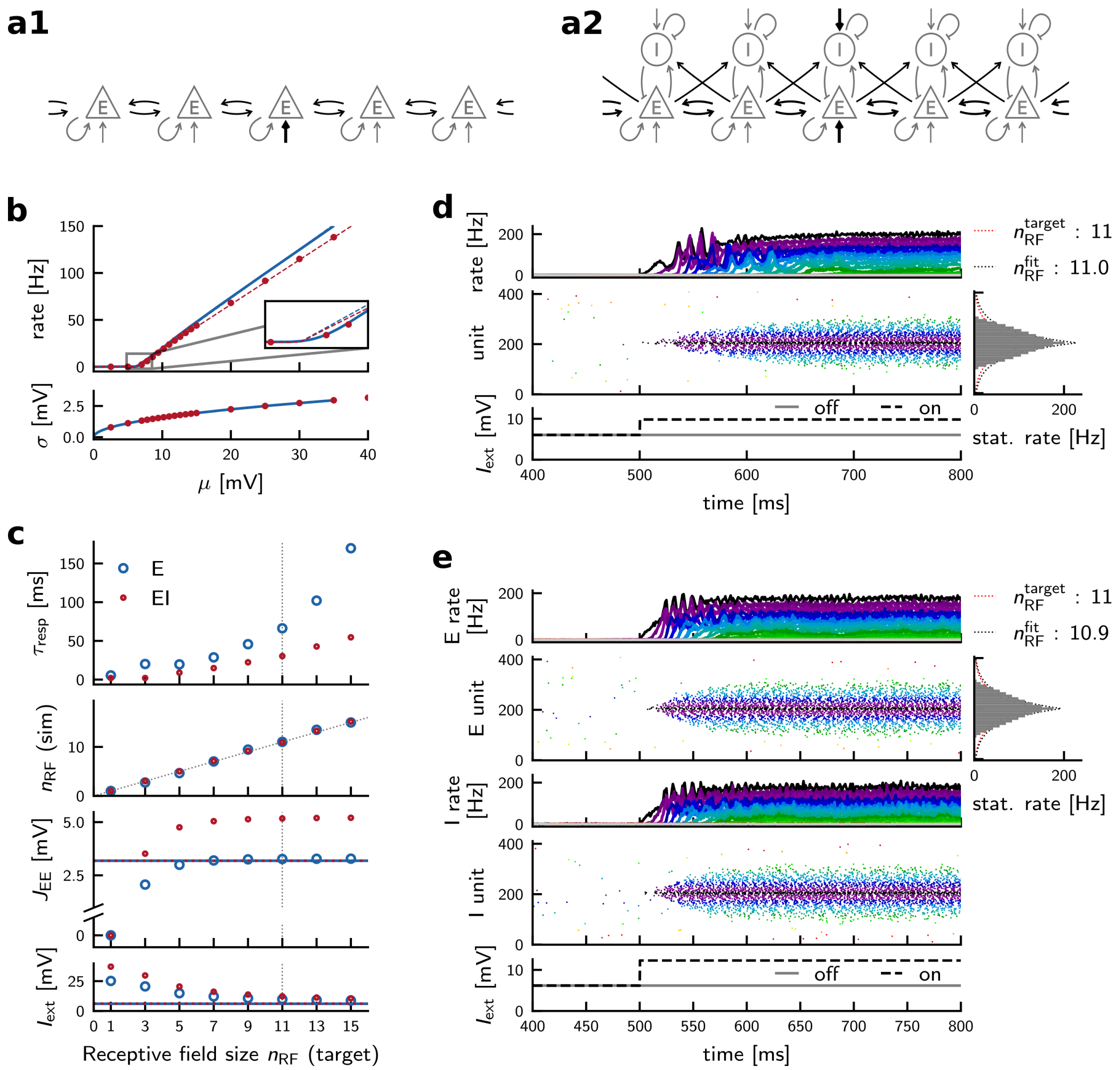

Figure I: **Faster response times in a homogeneous balanced network.** **(a1)** Wiring diagram of the purely excitatory network with fixed within-population coupling (grey). Only the across-population coupling depends on RF size (black). **(a2)** Wiring diagram of the excitatory-inhibitory network. Every EI feature population is an inhibition-dominated, balanced network in itself. Both excitatory and inhibitory feature populations receive tuned feedforward (and lateral) input of the same strength. **(b)** Blue: Transfer function of a single excitatory neuron in the E-only network. Red: Transfer function of a single excitatory neuron embedded in one of the EI-nodes shown in (a2); the firing rate is given as a function of the input that is provided to both E- and I-subpopulations. Inhibitory neurons have an identical transfer function. Dashed lines: threshold-linear fit (E/blue: $\mu_0 = 6.05$ mV, $g = 5.23$ Hz/mV; EI/red: $\mu_0 = 6.18$ mV, $g = 4.85$ Hz/mV). **(c)** From top to bottom: (i) Response time of E-only vs EI-network. (ii) Simulated vs. target RF size. (iii) Excitatory synaptic strength within (horizontal lines) and across (markers) populations, in the E-network (blue) and EI-network (red), respectively. Both the E-only and the EI-network have the same within-population excitatory coupling strength. Shown here are only the numerically optimized (across-population) coupling strengths. The analytical approximations have been omitted for clarity. (iv) Feedforward input depending on RF size. Horizontal line: background input (gray arrows in (a1),(a2)). Markers: Input to stimulated population(s) (black arrows in (a1),(a2)). **(d)** Example network dynamics for RF size 11, E-only network. **(e)** Example network dynamics for RF size 11, EI-network. The spread of activity from the stimulated population to the neighboring populations happens faster than in the E-only network.

References

1. Dayan P, Abbott L. Theoretical Neuroscience: Computational and Mathematical Modeling of Neural Systems. Cambridge: MIT Press; 2001.

2. Gray RM. Toeplitz and Circulant Matrices: A Review. Foundations and Trends in Communications and Information Theory. 2006;2(3):155–239.

3. Attwell D, Laughlin SB. An Energy Budget for Signaling in the Grey Matter of the Brain. Journal of Cerebral Blood Flow & Metabolism. 2001;21(10):1133–1145. doi:10.1097/00004647-200110000-00001.

4. Howarth C, Gleeson P, Attwell D. Updated Energy Budgets for Neural Computation in the Neocortex and Cerebellum. Journal of Cerebral Blood Flow & Metabolism. 2012;32(7):1222–1232. doi:10.1038/jcbfm.2012.35.

5. Sacramento J, Wichert A, Van Rossum MCW. Energy Efficient Sparse Connectivity from Imbalanced Synaptic Plasticity Rules. PLOS Computational Biology. 2015;11(6):e1004265. doi:10.1371/journal.pcbi.1004265.

6. Welzel O, Henkel AW, Stroebel AM, Jung J, Tischbirek CH, Ebert K, et al. Systematic Heterogeneity of Fractional Vesicle Pool Sizes and Release Rates of Hippocampal Synapses. Biophysical Journal. 2011;100(3):593–601. doi:10.1016/j.bpj.2010.12.3706.

7. Holler S, Köstinger G, Martin KAC, Schuhknecht GFP, Stratford KJ. Structure and Function of a Neocortical Synapse. Nature. 2021;591(7848):111–116. doi:10.1038/s41586-020-03134-2.

8. Kirischuk S, Grantyn R. Inter-Bouton Variability of Synaptic Strength Correlates With Heterogeneity of Presynaptic Ca $^{2+}$ Signals. Journal of Neurophysiology. 2002;88(4):2172–2176. doi:10.1152/jn.2002.88.4.2172.

9. Loebel A. Multiquantal Release Underlies the Distribution of Synaptic Efficacies in the Neocortex. Frontiers in Computational Neuroscience. 2009;3. doi:10.3389/neuro.10.027.2009.

10. Silver RA, Lübke J, Sakmann B, Feldmeyer D. High-Probability Uniquantal Transmission at Excitatory Synapses in Barrel Cortex. Science. 2003;302(5652):1981–1984. doi:10.1126/science.1087160.

11. Corless RM, Gonnet GH, Hare DEG, Jeffrey DJ, Knuth DE. On the LambertW Function. Advances in Computational Mathematics. 1996;5(1):329–359. doi:10.1007/BF02124750.

12. Derezinski J. Department of Mathematical Methods in Physics Faculty of Physics University of Warsaw Pasteura 5, 02-093 Warszawa, Poland; 2024. Available from: `https://www.fuw.edu.pl/~derezins/bessel.pdf`.

13. Brunel N, Hakim V. Fast Global Oscillations in Networks of Integrate-and-Fire Neurons with Low Firing Rates. Neural Computation. 1999;11(7):1621–1671. doi:10.1162/089976699300016179.

14. Sanzeni A, Histed MH, Brunel N. Response Nonlinearities in Networks of Spiking Neurons. PLOS Computational Biology. 2020;16(9):e1008165. doi:10.1371/journal.pcbi.1008165.
